# Sensing of mycobacterial arabinogalactan by galectin‐9 exacerbates mycobacterial infection

**DOI:** 10.15252/embr.202051678

**Published:** 2021-05-13

**Authors:** Xiangyang Wu, Yong Wu, Ruijuan Zheng, Fen Tang, Lianhua Qin, Detian Lai, Lu Zhang, Lingming Chen, Bo Yan, Hua Yang, Yang Wang, Feifei Li, Jinyu Zhang, Fei Wang, Lin Wang, Yajuan Cao, Mingtong Ma, Zhonghua Liu, Jianxia Chen, Xiaochen Huang, Jie Wang, Ruiliang Jin, Peng Wang, Qin Sun, Wei Sha, Liangdong Lyu, Pedro Moura‐Alves, Anca Dorhoi, Gang Pei, Peng Zhang, Jiayu Chen, Shaorong Gao, Felix Randow, Gucheng Zeng, Chang Chen, Xin‐Shan Ye, Stefan H E Kaufmann, Haipeng Liu, Baoxue Ge

**Affiliations:** ^1^ Shanghai Key Lab of Tuberculosis Shanghai Pulmonary Hospital Tongji University School of Medicine Shanghai China; ^2^ State Key Laboratory of Natural and Biomimetic Drugs School of Pharmaceutical Sciences Peking University Beijing China; ^3^ State Key Laboratory of Genetic Engineering Institute of Genetics School of Life Science Fudan University Shanghai China; ^4^ Department of Microbiology Key Laboratory for Tropical Diseases Control of the Ministry of Education Zhongshan School of Medicine Sun Yat‐sen University Guangzhou China; ^5^ Shanghai Public Health Clinical Center Fudan University Shanghai China; ^6^ Department of TB Shanghai Pulmonary Hospital Tongji University School of Medicine Shanghai China; ^7^ Key Laboratory of Medical Molecular Virology of the Ministry of Education/Ministry of Health School of Basic Medical Sciences Fudan University Shanghai China; ^8^ Department of Immunology Max Planck Institute for Infection Biology Berlin Germany; ^9^ Ludwig Institute for Cancer Research Nuffield Department of Medicine University of Oxford Oxford UK; ^10^ Institute of Immunology Friedrich‐Loeffler‐Institut Greifswald–Insel Riems Germany; ^11^ Department of Thoracic Surgery Shanghai Pulmonary Hospital Tongji University School of Medicine Shanghai China; ^12^ Clinical and Translational Research Center of Shanghai First Maternity and Infant Hospital Shanghai Key Laboratory of Signaling and Disease Research School of Life Sciences and Technology Tongji University Shanghai China; ^13^ Division of Protein and Nucleic Acid Chemistry MRC Laboratory of Molecular Biology Cambridge UK; ^14^ Hagler Institute for Advanced Study at Texas A&M University College Station TX USA; ^15^ Max Planck Institute for Biophysical Chemistry Göttingen Germany; ^16^ Clinical and Translational Research Center Shanghai Pulmonary Hospital Tongji University School of Medicine Shanghai China; ^17^ Central Laboratory Shanghai Pulmonary Hospital Tongji University School of Medicine Shanghai China

**Keywords:** galectin‐9, matrix metalloproteinases, mycobacterial arabinogalactan, transforming growth factor β‐activated kinase 1, virulence factor, Microbiology, Virology & Host Pathogen Interaction, Signal Transduction

## Abstract

Mycobacterial arabinogalactan (AG) is an essential cell wall component of mycobacteria and a frequent structural and bio‐synthetical target for anti‐tuberculosis (TB) drug development. Here, we report that mycobacterial AG is recognized by galectin‐9 and exacerbates mycobacterial infection. Administration of AG‐specific aptamers inhibits cellular infiltration caused by *Mycobacterium tuberculosis* (*Mtb*) or *Mycobacterium bovis* BCG, and moderately increases survival of *Mtb*‐infected mice or *Mycobacterium marinum‐*infected zebrafish. AG interacts with carbohydrate recognition domain (CRD) 2 of galectin‐9 with high affinity, and galectin‐9 associates with transforming growth factor β‐activated kinase 1 (TAK1) via CRD2 to trigger subsequent activation of extracellular signal‐regulated kinase (ERK) as well as induction of the expression of matrix metalloproteinases (MMPs). Moreover, deletion of galectin‐9 or inhibition of MMPs blocks AG‐induced pathological impairments in the lung, and the AG‐galectin‐9 axis aggravates the process of *Mtb* infection in mice. These results demonstrate that AG is an important virulence factor of mycobacteria and galectin‐9 is a novel receptor for *Mtb* and other mycobacteria, paving the way for the development of novel effective TB immune modulators.

## Graphical abstract

Arabinogalactan is a virulence factor of mycobacteria. Binding of galectin‐9 to mycobacterial arabinogalactan triggers matrix metalloproteinases via TAK1‐ERK signaling, which promotes mycobacterial infection and increases lung injury.

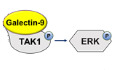

## Introduction

*Mycobacterium tuberculosis* (*Mtb*) infection leads to active tuberculosis (TB) in millions of people annually (Kaufmann *et al,* 2018). In 2019, 10 million new TB cases and 1.4 million deaths were reported (WHO Global Tuberculosis Report, 2020). An estimated quarter of the world's population is infected with *Mtb*, but only 5–10% of infected individuals succumb to active TB disease (WHO Global Tuberculosis Report, 2020). The pathogenesis of TB is shaped by an intricate balance between host immunity and *Mtb* infection (Orme *et al,* 2015). Innate immune cells respond to *Mtb* infection to trigger a cascade of cellular events including phagocytosis, apoptosis, autophagy, inflammasome activation, and nitric oxide production to curb the intracellular survival of *Mtb*. Moreover, dendritic cells (DCs) migrate from the site of infection to the regional lymph node, where they prime naïve T cells to mount an *Mtb*‐specific acquired immune response including enduring memory which restricts bacterial growth (Ernst, 2012; O'Garra *et al,* 2013; Robinson *et al,* 2015). On the other hand, *Mtb* has developed numerous strategies to evade or inhibit host innate immunity (Hmama *et al,* 2015; Liu *et al,* 2017a; Chai *et al,* 2020).

Pattern recognition receptors (PRRs) of innate immune cells sense *Mtb*‐derived conserved pathogen‐associated molecular patterns (PAMPs) which trigger a cascade of innate immune responses (Mortaz *et al,* 2015; Stamm *et al,* 2015). Surface receptors such as Toll‐like receptor (TLRs), C‐type lectin receptors (CLRs), and scavenger receptors (SRs) (Stamm *et al,* 2015) sense cell wall‐associated glycolipids or glycoproteins and secreted proteins to initiate phagocytosis, apoptosis, or production of immune‐regulatory cytokines (Wang *et al,* 2017; Wang *et al,* 2019). Endosomal PRRs including TLR9 and TLR3 sense *Mtb*‐derived DNA and RNA, respectively, to induce the production of cytokines such as interleukin (IL)‐10 (Ito *et al,* 2009; Bai *et al,* 2014). Cytosolic receptors such as absent in melanoma 2 (AIM2) and NOD‐, LRR‐, and pyrin domain containing 3 (NLRP3) sense cytosolic DNA or Early secretory antigenic target‐6 (ESAT‐6) to activate the inflammasome for processing of IL‐1β (Mishra *et al,* 2010; Saiga *et al,* 2012). Activation of cytosolic nucleotide‐binding oligomerization domain 2 (NOD2) by muramyl dipeptide (MDP) induces the production of antimicrobial peptide LL37 and autophagy (Juarez *et al,* 2012). *Mtb‐*derived extracellular DNA (eDNA) and cyclic dinucleotide c‐di‐AMP are sensed by cytosolic DNA sensor cyclic GMP‐AMP synthase (cGAS) and adaptor stimulator of interferon genes (STING), respectively (Dey *et al,* 2015; Watson *et al,* 2015; Dey *et al,* 2017) to induce production of type I interferons. Moreover, *Mtb* escapes into the cytosol (van der Wel *et al,* 2007), where ubiquitin‐coated *Mtb* was recognized by autophagy receptors such as p62 and NDP52 to induce xenophagy (Perrin *et al,* 2004). On the other hand, several *Mtb*‐secreted proteins such as ESAT‐6 and PtpA interact with PRRs to suppress host immunity (Pathak *et al,* 2007; Wang *et al,* 2015). Collectively, the identification of novel receptors involved in dynamic sensing of *Mtb*‐derived components during infection is critical for understanding host–pathogen interactions and pathogenesis of TB.

The *Mtb* cell wall comprises various components that interact with host immune molecules to regulate the pathogenesis of *Mtb* infection. Mannosylated lipoarabinomannan (ManLAM), a major glycan of *Mtb*, is recognized by dendritic cell (DC)‐associated C‐type lectin‐2 (Dectin‐2, also known as CLEC6A) which induces the production of both pro‐ and anti‐inflammatory cytokines in DCs (Yonekawa *et al,* 2014). Mycobacterial cell wall glycolipid trehalose‐6,6´ dimycolate (TDM or cord factor) is sensed by Dectin‐3 which induces expression of the Mincle gene. The interaction of Mincle with TDM further activates the NF‐κB signaling pathway to produce pro‐inflammatory cytokines (Ishikawa *et al,* 2009; Werninghaus *et al,* 2009; Zhao *et al,* 2014). Arabinogalactan (AG), together with peptidoglycan and mycolic acid, forms the cell wall core of *Mtb* (Jankute *et al,* 2015; Grzegorzewicz *et al,* 2016). Remarkably, the arabinosyltransferases EmbA, EmbB, and EmbC, which are critical for AG synthesis, serve as targets for anti‐TB drug action (Escuyer *et al,* 2001; Goude *et al,* 2009; Cui *et al,* 2014; Zhang *et al,* 2020a; Zhang *et al,* 2020b). Recently, the structures of arabinosyltransferases targeted by the anti‐TB drug ethambutol have been elucidated (Zhang *et al,* 2020a; Zhang *et al,* 2020b). However, it remains unclear whether mycobacterial AG qualifies as a PAMP or a virulence factor of *Mtb* and its PRR remains unidentified due to the lack of purified mycobacterial AG and the unavailability of an AG‐deficient mycobacteria (Toyonaga *et al,* 2016).

Based on our report of the first complete synthesis of an AG composed of 92 mono‐saccharide units (Wu *et al,* 2017), we harnessed chemically synthesized AG for the evaluation of its biological functions. By knockdown of genes involved in AG synthesis and generation of AG‐specific aptamers, we demonstrate that AG is a virulence factor of *Mtb* and aggravates mycobacterial infection. Moreover, galectin‐9, a member of the β‐galactoside binding gene family, was identified as a receptor for AG which signals through the TAK1‐ERK‐MMP axis to trigger pathological impairments in the lung. Our findings form the basis for novel intervention strategies against TB which target this axis.

## Results

### AG exacerbates mycobacterial infection

**Figure 1:**
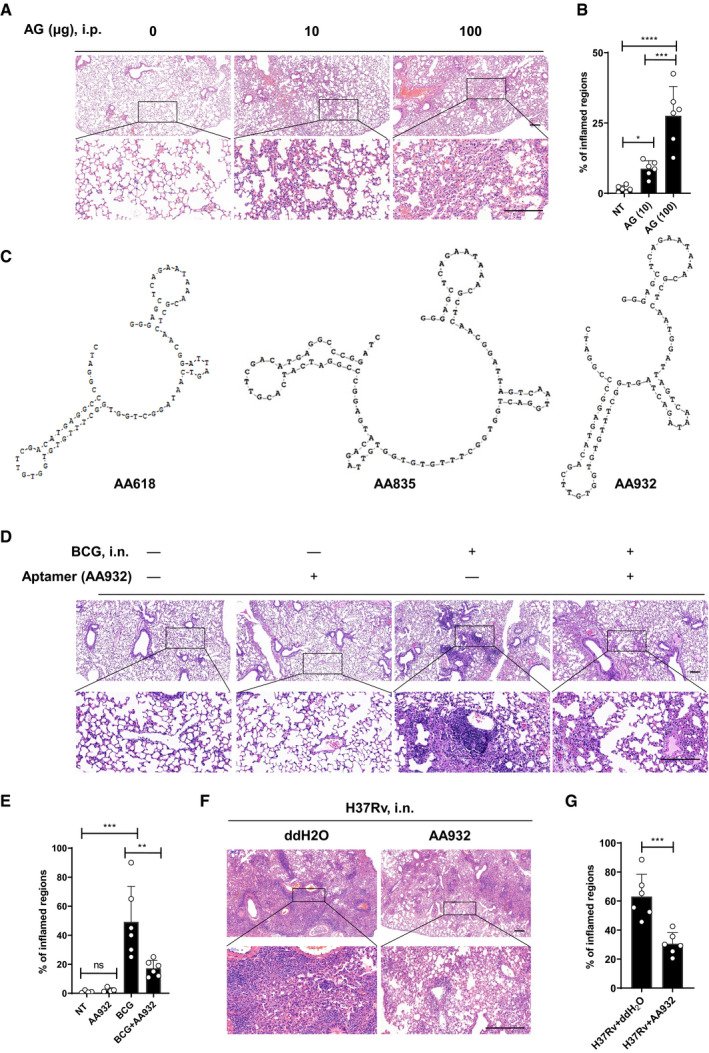
AG is a virulence factor of mycobacteria A, BC57BL/6 mice were left untreated (NT) or were intraperitoneally treated with indicated amounts of AG for 3 days. Lung sections stained with hematoxylin and eosin(H&E) (A) and quantification of lung lesion burden from H&E‐stained sections (B).CSequence and secondary structure of identified aptamers against AG as predicted with DNAMAN version 6.0.D–GH&E staining of lung sections from mice 4 weeks after intranasal infection with *M. bovis* BCG (D and E) or *Mtb* H37Rv (F and G) in the absence or presence of intranasally administrated AG aptamers (1 μg) once at a 1‐week interval. Quantification of lung inflamed regions shown in (E and G). Data information: Data in (B, E, G) are means ± SD of indicated numbers of mice from 1 of *n* = 3 independent experiments with similar results and each symbol represents 1 mouse. Data in (A, D, F) are representative of *n* = 3 independent experiments. One‐way ANOVA followed by Dunnett's *post hoc* test (B and E) and Student’s *t‐*test (G) were used for statistical analysis, respectively. ns, not significant; *P < 0.05; **P < 0.01; ***P < 0.001; ****P < 0.0001. Scale bar, 200 μm.

Given the lung is a main port of entry for *Mtb* and the primary organ of pulmonary TB (Kaufmann *et al,* 2014), we interrogated whether AG affects the lung by employing the chemically synthesized AG composed of 92 mono‐saccharide units (Wu *et al,* 2017). In an experimental mouse model, intraperitoneal administration of AG caused profound cellular infiltrations in the lung as demonstrated by H&E staining in a dose‐dependent manner (Fig 1A and B). Similarly, intravenous injection of AG caused pathological impairments of pulmonary tissue (Appendix Fig S1A–C). Lung tissue damage was further exacerbated when AG was emulsified in Freund's incomplete adjuvant (FIA) (Bekierkunst, 1968; Bekierkunst *et al,* 1969; Yarkoni & Rapp, 1977; Lee *et al,* 2012) (Appendix Fig S1A–C).

To further verify the function of AG, we performed an *in vitro* selection process called Systematic Evolution of Ligands by Exponential Enrichment (SELEX) of aptamers (Tuerk & Gold, 1990; Bock *et al,* 1992) against AG (Appendix Fig S2A), which has been successfully applied for the screening of aptamers targeting *Mtb* previously (Qin *et al,* 2014; Aimaiti *et al,* 2015; Sun *et al,* 2016; Tang *et al,* 2016; Zhang *et al,* 2017; Golichenari *et al,* 2018). Enzyme‐linked immunosorbent assay (ELISA) was employed to determine respective affinities of aptamers against AG. We systematically monitored the selection process to obtain additional aptamers against AG, thereby improving the dynamic evolution of aptamers against AG. After nine rounds of selection, the maximum level of affinity was enriched and three aptamers (AA618, AA835, AA932) with high affinity but different structures were selected (Fig 1C). Among them, the frequency of aptamer AA932 sequence was highest, which was deemed as the preponderant aptamer. In an intranasal mycobacterial infection animal model (Zheng *et al,* 2018b; Wang *et al,* 2019), AG‐specific aptamers markedly inhibited the pathological impairments in the lung caused by infection with either Bacillus Calmette–Guérin (BCG) (Fig 1D and E) or *Mtb* H37Rv (Fig 1F and G). Moreover, intranasal administration of AG‐specific aptamers moderately enhanced the survival rate of severe combined immunodeficiency (SCID) mice infected with a lethal dose of *Mtb* H37Rv (Appendix Fig S2B). Consistently, in a zebrafish larvae infection model (Takaki *et al,* 2013), preincubation of AG‐specific aptamers significantly increased the survival rate of zebrafish larvae infected with *Mycobacterium marinum* (Appendix Fig S2C), while the AG‐specific aptamers alone did not show any effect on the survival of zebrafish larvae (Appendix Fig S2C). Taken together, these results point to AG as an important virulence factor of *Mtb* and other mycobacteria.

### AG induces the expression of MMPs

**Figure 2:**
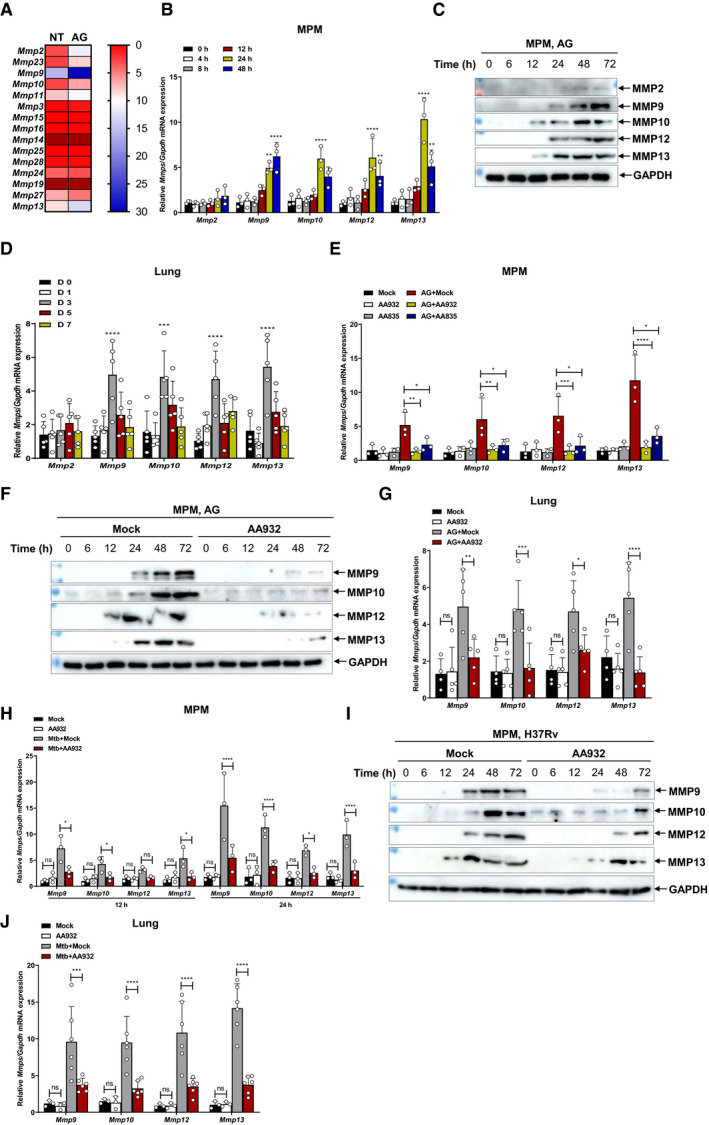
AG induces expression of MMPs Heat map showing RPKM (Reads Per Kilobase per million of mapped reads) mean values of *Mmps* from mouse peritoneal macrophages (MPM) stimulated with AG (1 μg/ml) for 24 h or left untreated (NT).Quantitative polymerase chain reaction (qPCR) analysis of *Mmps* including *Mmp2*, *Mmp9*, *Mmp10*, *Mmp12*, and *Mmp13* mRNA from mouse peritoneal macrophages stimulated with AG (1 μg/ml) for indicated times.Immunoblots of cell supernatants to analyze secreted MMP2, MMP9, MMP10, MMP12, and MMP13 by mouse peritoneal macrophages stimulated with AG (1 μg/ml) for indicated times; GADPH of cell lysates served as a loading control.qPCR analysis of *Mmps* including *Mmp2*, *Mmp9*, *Mmp10*, *Mmp12*, and *Mmp13* from the lungs of mice at indicated days post‐intraperitoneal administration of AG (100 μg).qPCR analysis of *Mmps* including *Mmp9*, *Mmp10*, *Mmp12*, and *Mmp13* from mouse peritoneal macrophages stimulated with AG (1 μg/ml) for 24 h in the absence or presence of AG aptamers (0.5 μg/ml).Immunoblots of cell supernatants to analyze secreted MMP9, MMP10, MMP12, and MMP13 by mouse peritoneal macrophages stimulated with AG (1 μg/ml) for indicated times in the absence or presence of AG aptamers (0.5 μg/ml); GADPH of cell lysates served as the loading control.qPCR analysis of *Mmps* including *Mmp9*, *Mmp10*, *Mmp12*, and *Mmp13* from the lungs of mice at 3 days post‐intraperitoneal administration of AG (100 μg) in the absence or presence of AG aptamers. AG aptamers (1 μg/mouse) were intranasally administrated per day.qPCR analysis of *Mmps* including *Mmp9*, *Mmp10*, *Mmp12*, and *Mmp13* from mouse peritoneal macrophages infected with H37Rv for 24 h (MOI = 5) in the absence or presence of AG aptamers (1 μg/ml).Immunoblots of cell supernatants to analyze secreted MMP9, MMP10, MMP12, and MMP13 by mouse peritoneal macrophages infected with H37Rv for indicated times (MOI = 5) in the absence or presence of AG aptamers (0.5 μg/ml); GADPH of cell lysates served as the loading control.qPCR analysis of *Mmps* including *Mmp9*, *Mmp10*, *Mmp12*, and *Mmp13* from lungs of mice intranasally infected with H37Rv (2 × 10^6^ cfu/mouse) for 4 weeks in the absence or presence of AG aptamers. AG aptamers (1 μg/mouse) were intranasally administrated once at a 1‐week interval. Data information: Data in (B, E, H) are means ± SD averaged from 3 independent experiments performed with technical triplicates and each symbol represents the mean of technical triplicates. Data in (D, G, J) are means ± SD of indicated numbers of mice from 1 of at least *n* = 2 independent experiments, and each symbol represents data from 1 mouse. Data (D, G, J) shown are representative of *n* = 2 (D) or *n* = 3 (G, J) independent experiments. Two‐way ANOVA followed by Tukey's *post hoc* test (B, D, E, G, H, J) was used for statistical analysis. *P < 0.05; **P < 0.01; ***P < 0.001; ****P < 0.0001. Source data are available online for this figure.

**Figure EV1:**
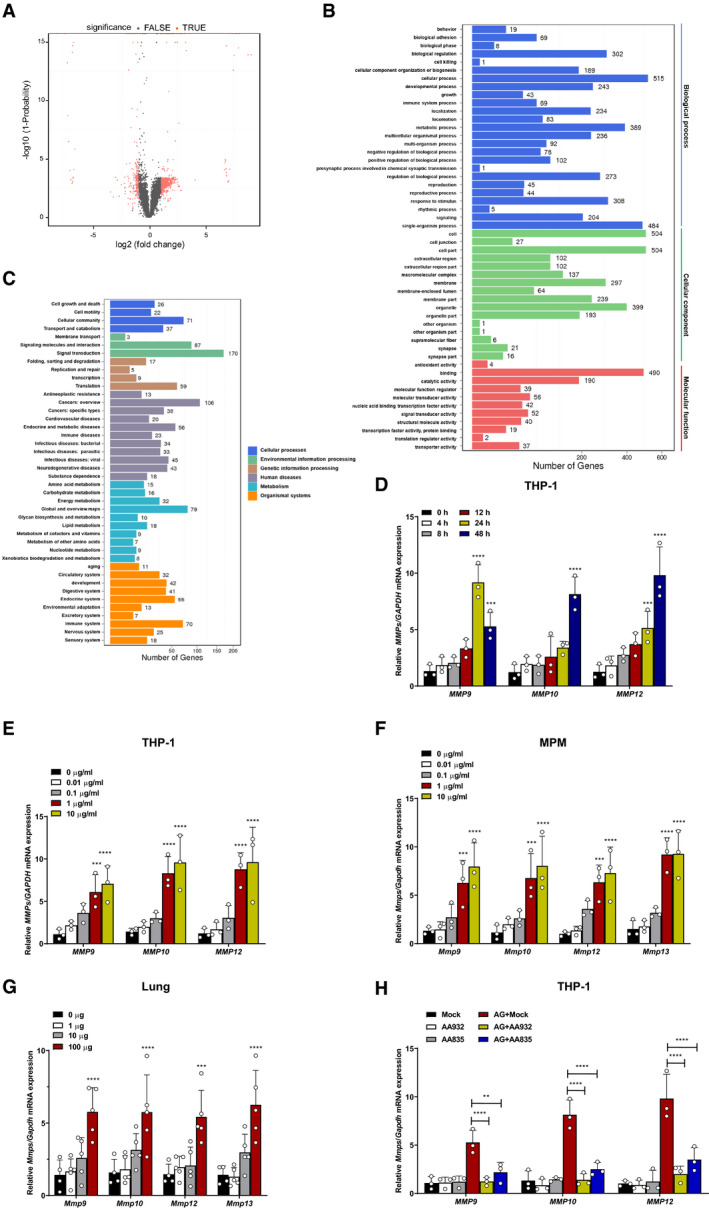
AG induces expression of MMPs AScatter plots of differentially expressed genes in the mouse peritoneal macrophages stimulated with AG (1 μg/ml) for 24 h as identified by RNA‐seq analysis. The RNA from the peritoneal macrophages was pooled and subjected to RNA‐seq.BGO class of gene expressions in mouse peritoneal macrophages stimulated with AG (1 μg/ml) for 24 h as identified by RNA‐seq analysis.CKEGG class of gene expressions in mouse peritoneal macrophages stimulated with AG (1 μg/ml) for 24 h as identified by RNA‐seq analysis.D, EqPCR analysis of *Mmps* including *Mmp9*, *Mmp10*, and *Mmp12* mRNA from THP‐1 cells stimulated with AG (1 μg/ml) for indicated times (D) or at indicated concentrations (μg/ml) for 48 h (E).FqPCR analysis of *Mmps* including *Mmp9*, *Mmp10*, *Mmp12*, and *Mmp13* mRNA from mouse peritoneal macrophages stimulated with AG at indicated concentrations (μg/ml) for 24 h.GqPCR analysis of *Mmps* including *Mmp9*, *Mmp10*, *Mmp12*, and *Mmp13* from the lungs of mice at indicated concentrations (μg) for 3 days post‐intraperitoneal administration of AG.HqPCR analysis of *Mmps* including *Mmp9*, *Mmp10*, and *Mmp12* from THP‐1 cells stimulated with AG (1 μg/ml) for 48 h left untreated or pretreated with AG aptamers AA932 or AA835 (0.5 μg/ml). Data information: Data in (D–F, H) are means ± SD averaged from 3 independent experiments performed with technical triplicates, and each symbol represents the mean of technical triplicates. Data in (G) are means ± SD of indicated mice from 1 of *n* = 3 independent experiments, and each symbol represents data from 1 mouse. One‐way ANOVA followed by Dunnett's *post hoc* test were used for statistical analysis, respectively. ns, not significant; **P < 0.01; ***P < 0.001; ****P < 0.0001.

Macrophages serve as both habitat and the first line of defense against *Mtb* (Pieters, 2008). To examine the effect of AG on macrophages, we treated macrophages with AG and analyzed the induced gene expression patterns by RNA sequencing. The GO pathway analysis revealed an enrichment of multiple matrix metalloproteinases (MMPs) (Figs 2A and EV1A–C). Considering the important role of MMPs in the pathogenesis of TB and the correlation of their levels with clinical and radiological markers of lung tissue destruction (Volkman *et al,* 2010; Ong *et al,* 2014; Parasa *et al,* 2017; Sabir *et al,* 2019; Kathamuthu *et al,* 2020), we therefore specifically focused on these genes in our study. Profound induction of MMPs including MMP9, MMP10, and MMP12 in response to AG stimulation was validated by quantitative RT–PCR in both murine and human macrophages (Figs 2B and EV1D–F). The secretion of MMPs including MMP9, MMP10, and MMP12 into the supernatants of mouse macrophages was further validated by Western blot (Fig 2C). Moreover, in lung tissues of mice treated with AG intraperitoneally, abundances of MMP transcripts were highly upregulated (Figs 2D and EV1G). Reciprocally, pretreatment of AG‐specific aptamers including AA932 and AA835 markedly reduced AG‐induced MMP gene expression in both murine and human macrophages (Figs 2E and EV1H). Consistently, treatment of AG aptamer AA932 abrogated AG‐stimulated secretion of MMPs in supernatants (Fig 2F). Finally, intranasal administration of AG‐specific aptamers profoundly decreased abundances of MMPs in pulmonary tissue of mice treated intraperitoneally with AG (Fig 2G). For in‐depth analysis of the function of AG in mycobacteria, we generated *M. marinum* mutants in which AG was diminished by CRISPR/Cas9‐mediated conditional knockdown of *MMAR_5356* and *MMAR_5357* (Appendix Fig S3A), that are homologs of *embA* and *embB* in *Mtb*, respectively, and are important for the biosynthesis of AG (Alderwick *et al,* 2015; Jankute *et al,* 2015; Dulberger *et al,* 2020; Zhang, *et al,* 2020a). Tetracycline (Tet) treatment led to significantly reduced expression of corresponding genes (Appendix Fig S3B), which subsequently diminished the expression level of MMPs in macrophages infected with *M. marinum* (Appendix Fig S3C). To further clarify whether these mutants regulate MMP expression by affecting mycobacterial growth, we measured the growth curves of the wild‐type and knockdown mutants of *M. marinum*. The data demonstrate that tetracycline treatment of wild‐type and *MMAR_5356* and *MMAR_5357* did not significantly affect the growth of *M. marinum* in 7H10 culture medium (Appendix Fig S3D–F). Moreover, treatment with AG‐specific aptamers markedly reduced the expression and secretion of MMPs in *Mtb*‐infected macrophages (Fig 2H and I). Consistent with these *in vitro* findings, intranasal administration of AG‐specific aptamers markedly reduced abundances of MMPs in the lung of mice infected with *Mtb* or BCG (Fig 2J, Appendix Fig S3G). Our data, therefore, identify AG as a mycobacterial MMP inducer in macrophages.

### AG activates ERK to induce MMPs

**Figure 3:**
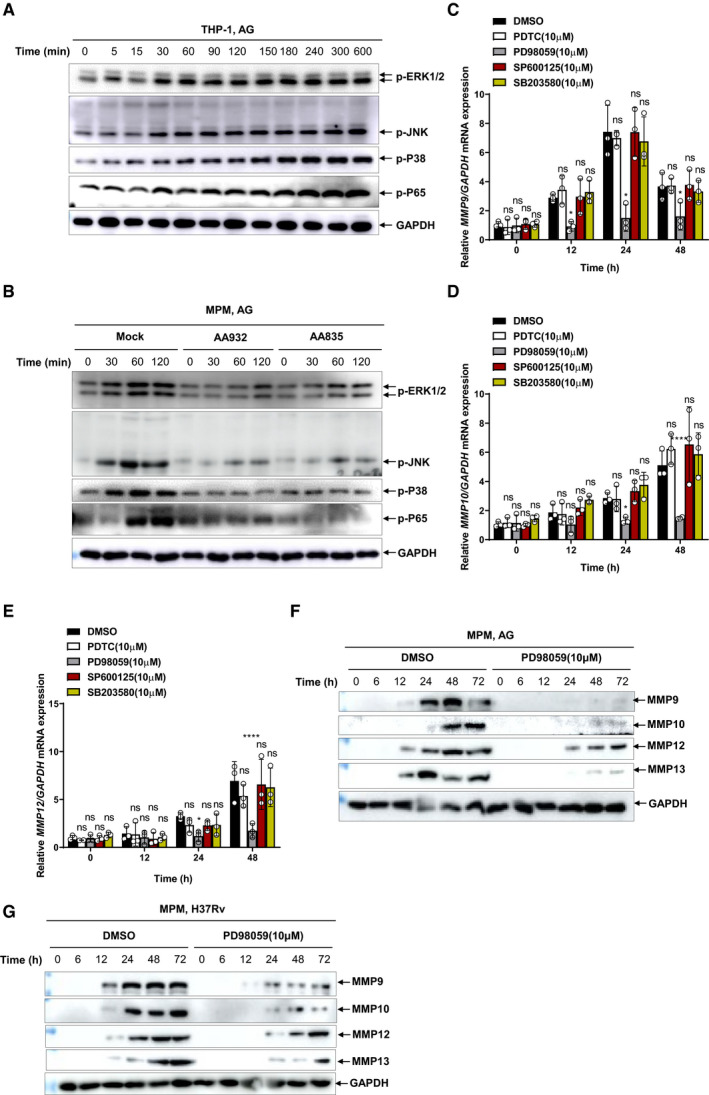
AG activates ERK to induce MMPs AImmunoblots of cell lysates were performed to analyze p‐ERK1/2, p‐JNK, p‐P38, and p‐P65 by THP‐1 cells stimulated with AG (1 μg/ml) for indicated times, and GADPH as a loading control.BImmunoblots of cell lysates were performed to analyze p‐ERK1/2, p‐JNK, p‐P38, and p‐P65 by mouse peritoneal macrophages stimulated with AG (1 μg/ml) for indicated times left untreated or pretreated with AG aptamer (1 μg/ml), and GADPH as a loading control.C–EqPCR analysis of *Mmps* including *Mmp9* (C), *Mmp10* (D), and *Mmp12* (E) from THP‐1 cells stimulated with AG (1 μg/ml) for 24 h in the absence or presence of different inhibitors targeting NF‐κB (PDTC), ERK (PD98059), JNK (SP600125), and p38 (SB203580) at the concentration of 10 μM.FImmunoblots of cell supernatants to analyze secreted MMP9, MMP10, MMP12, and MMP13 by mouse peritoneal macrophages stimulated with AG (1 μg/ml) for indicated times in the absence or presence of inhibitor targeting ERK (PD98059) at the concentration of 10 μM; GADPH of cell lysates served as the loading control.GImmunoblots of cell supernatants to analyze secreted MMP9, MMP10, MMP12, and MMP13 by mouse peritoneal macrophages infected with H37Rv for indicated times (MOI = 5) in the absence or presence of inhibitor targeting ERK (PD98059) at the concentration of 10 μM; GADPH of cell lysates served as the loading control. Data information: Data in (A and B) are representative of *n* = 3 independent experiments. Data in (C–E) are means ± SD averaged from *n* = 3 independent experiments performed with technical triplicates, and each symbol represents the mean of technical triplicates. Two‐way ANOVA followed by Dunnett's *post hoc* test (C–E) were used for statistical analysis. ns, not significant; *P < 0.05; ****P < 0.0001. Source data are available online for this figure.

We next investigated the signaling events involved in MMP induction by AG. Treatment with AG activated MAPKs including ERK, p38, and JNK, as well as NF‐κB in both human and murine macrophages (Fig 3A and B). Moreover, administration of AG‐specific aptamers impaired AG‐induced activation of MAPKs and NF‐κB signaling pathways (Fig 3B). To further define the downstream pathway involved in the AG‐induced expression of MMPs, macrophages were treated with AG in the presence of specific inhibitors targeting NF‐κB (PDTC), ERK (PD98059), JNK (SP600125), and p38 (SB203580), respectively. Specific inhibition of ERK by PD98059 significantly reduced AG‐induced expression of MMPs (Fig 3C–E). Consistently, PD98059‐mediated inhibition of ERK markedly reduced AG‐stimulated secretion of MMPs as well (Fig 3F). Moreover, the secretion of MMPs in mouse macrophages in response to *Mtb* infection was dramatically attenuated in the presence of ERK inhibitor (Fig 3G). We, therefore, conclude that AG activated the ERK signaling pathway to induce MMPs expression.

### Lung injury induced by AG depends on MMPs

**Figure 4:**
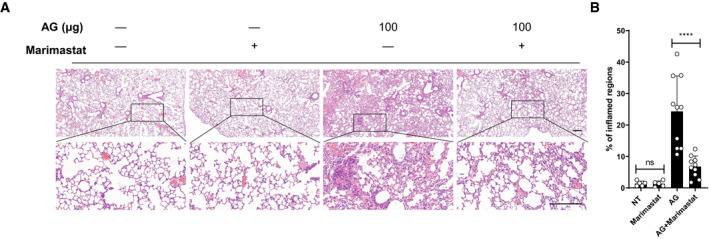
Lung injury induced by AG depends on MMPs A, BC57BL/6 mice were left untreated (NT) or were intraperitoneally treated with indicated amounts of AG for 3 days in the absence or presence of the MMP inhibitor marimastat (10 mg/kg) given intraperitoneally prior to AG stimulation. Lung sections stained with H&E (A) and quantification of lung lesion burden from H&E‐stained sections (B). Data information: Data in (A) are representative of *n* = 3 independent experiments. Data in (B) are means ± SD of indicated numbers of mice from one of *n* = 3 independent experiments and each symbol represents data from 1 mouse. One‐way ANOVA followed by Dunnett's *post hoc* test (B) was used for statistical analysis. ns, not significant; ****P < 0.0001. Scale bar, 200 μm.

Considering the correlation of MMPs with the severity of TB (Hrabec *et al,* 2002; Ong *et al,* 2014; Kubler *et al,* 2015; Parasa *et al,* 2017; Sabir *et al,* 2019; Kumar *et al,* 2020), and the association of MMPs abundance in the lung with the severity of pulmonary pathology (Figs 1F–G, 2E and EV1G, Appendix Fig S1A–C), we further clarified whether MMPs contribute to the pathogenic effect of AG *in vivo*. We treated mice with the MMPs inhibitor marimastat (Skipper *et al,* 2009) and examined AG‐induced pathological changes in the lung. Inhibition of MMPs significantly reduced lung pathology of mice challenged with AG (Fig 4A and B). We conclude that AG caused lung injury through MMPs induction.

### AG interacts with galectin‐9

**Figure EV2:**
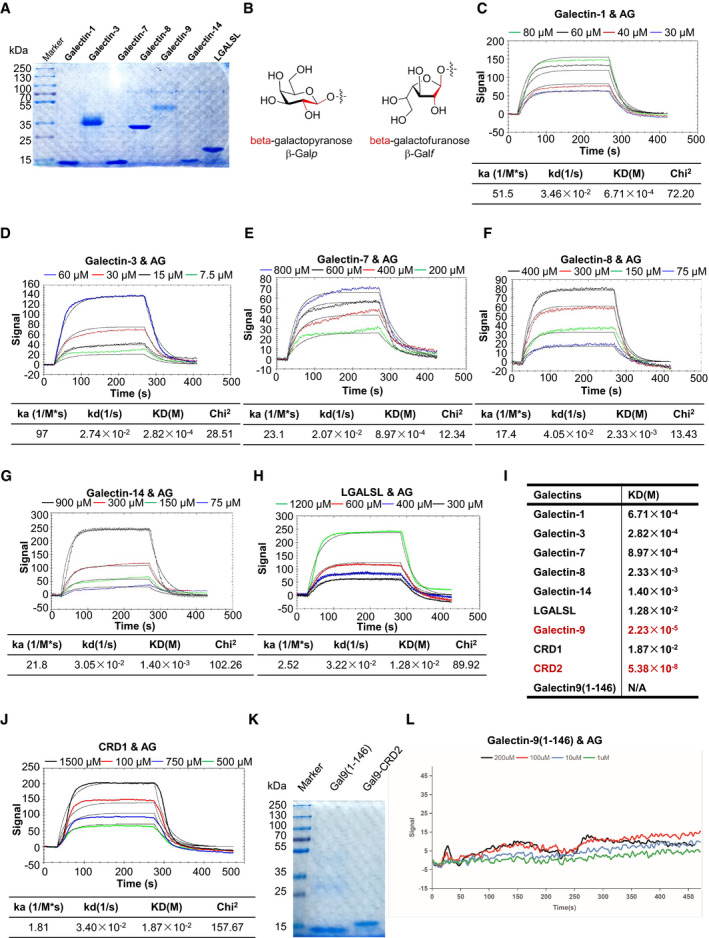
Interaction of AG with galectins ACoomassie blue staining of galectin‐1, galectin‐3, galectin‐7, galectin‐8, galectin‐9, galectin‐14, and galectin‐related protein (LGALSL) post–SDS–PAGE analysis.BChemical structures and conformations of β‐galactofuranoside and β‐galactopyranoside.C–ISPR assay of interactions of AG with indicated galectins including galectin‐1 (C), galectin‐3 (D), galectin‐7 (E), galectin‐8 (F), galectin‐14 (G), LGASL (H), and a summary table of KD (I). Curve fittings to a 1:1 Langmuir‐binding model calculated with TraceDrawer are shown as smooth black lines. The binding affinity of galectin‐9 and CRD2 to AG is highlighted in (I) in red.JSPR assay of interactions of AG with CRD1 of galectin‐9.KCoomassie blue staining of galectin‐9(1–146) and CRD2 of galectin‐9 post–SDS–PAGE analysis.LSPR assay of interactions of AG with galectin‐9(1–146).

**Figure 5:**
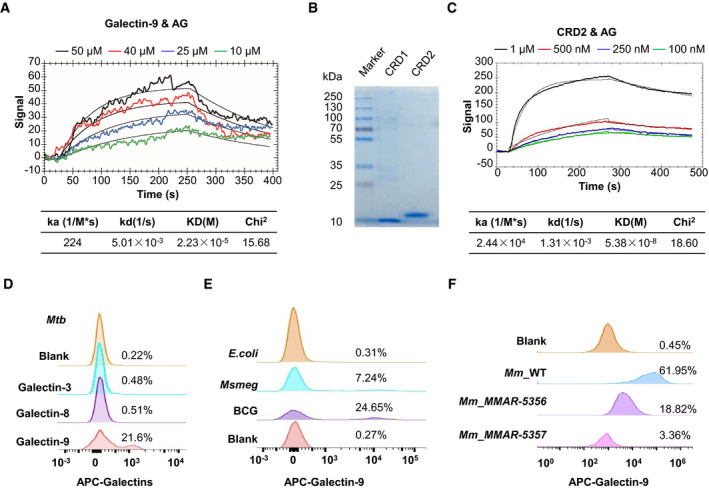
AG interacts with galectin‐9 Surface plasmon resonance (SPR) assay of the direct interaction of AG with galectin‐9. Curve fittings to a 1:1 Langmuir‐binding model calculated with TraceDrawer are shown as smooth black lines.Coomassie blue‐stained SDS–PAGE of carbohydrate recognition domain CRD1 and CRD2 of galectin‐9. Data are representative of *n* = 3 independent experiments.SPR assay of the interaction of AG with CRD2 of galectin‐9. Curve fittings to a 1:1 Langmuir‐binding model calculated with TraceDrawer are shown as smooth black lines.FACS assay showing interactions of *Mtb* H37Rv with different galectins including galectin‐3, galectin‐8, and galectin‐9. The blank control was H37Rv staining with APC‐anti‐rabbit antibody alone.FACS assay of interactions of galectin‐9 with mycobacteria including *M. bovis* BCG and *M. smegmatis* mc^2^155 as well as *E. coli*. The blank control was BCG staining with APC‐anti‐rabbit antibody alone. Data shown are representative of *n* = 3 independent experiments.FACS assay of interactions of galectin‐9 with wild‐type *M. marinum* (Mm_WT) and MMAR‐5356 and MMAR‐5357 mutants of *M. marinum* treated with Tet. The blank control was Mm_WT staining with APC‐anti‐rabbit antibody alone. Data shown are representative of *n* = 3 independent experiments.

**Figure EV3:**
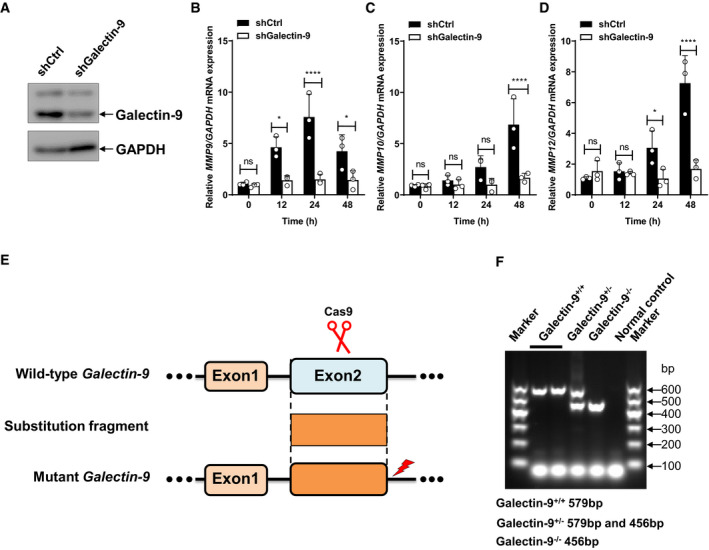
Galectin‐9 is essential for AG‐induced expression of MMPs AImmunoblots of cell lysates of THP‐1 cells stably transfected with scrambled shRNA or shRNA targeting galectin‐9.B–DqPCR analysis of *Mmps* including *Mmp9* (B), *Mmp10* (C), and *Mmp12* (D) mRNA from control or Galectin‐9 knockdown THP‐1 cells stimulated with AG (1 μg/ml) for indicated times.EDiagram showing the gRNA‐targeting genome sites.FIdentification of galectin‐9 KO mice with PCR. Data information: Data in (B–D) are means ± SD averaged from *n* = 3 independent experiments performed with technical triplicates, and each symbol represents the mean of technical triplicates. Data in (A, F) are representative of at least *n* = 2 independent experiments. Two‐way ANOVA followed by Tukey's *post hoc* test (B–D) was used for statistical analysis, respectively. ns, not significant; *P < 0.05; ****P < 0.0001. Source data are available online for this figure.

**Figure EV4:**
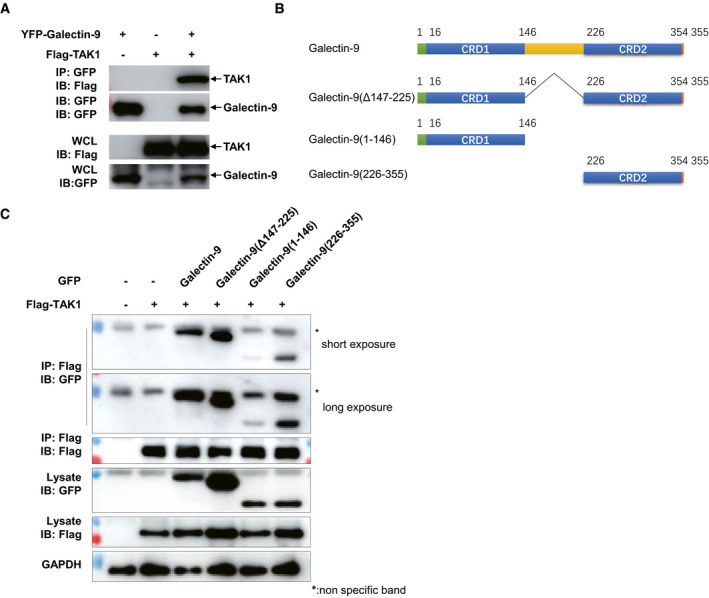
Interaction of Galectin‐9 with TAK1 Immunoblots and immunoprecipitation analysis of lysates of HEK293T cells transfected with various plasmids as indicated.Diagram showing various constructs of plasmids including Galectin‐9, Galectin‐9(Δ147–225), Galectin‐9(1–146), and Galectin‐9(226–355).Immunoblots and immunoprecipitation of lysates from HEK293T cells transfected with plasmids as indicated. Source data are available online for this figure.

**Figure EV5:**
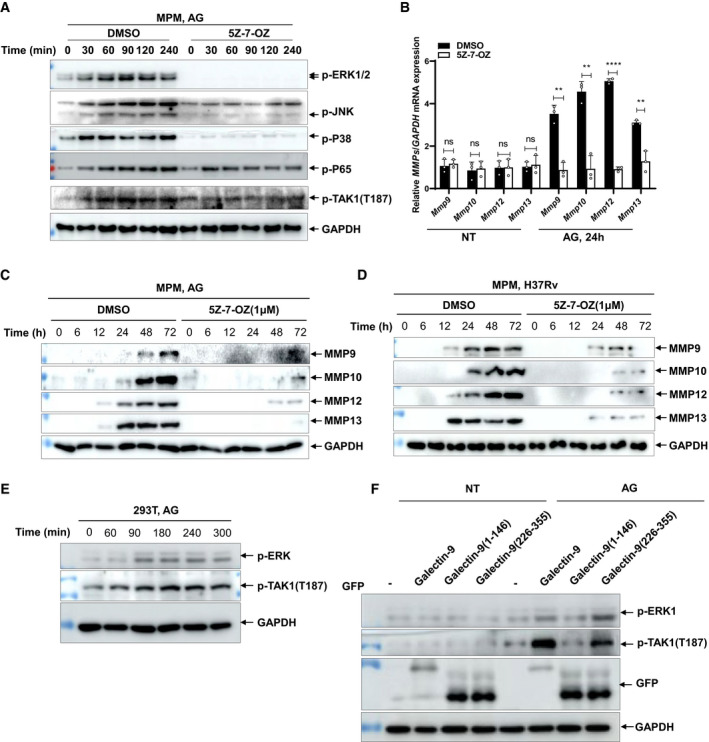
AG induces MMPs via TAK1 activation Immunoblots of cell lysates of peritoneal macrophages stimulated with AG (1 μg/ml) in the absence or presence of TAK1 inhibitor 5Z‐7‐OZ (1 μM) for indicated times. Data are representative of *n* = 3 independent experiments.qPCR analysis of *Mmps* including *Mmp9*, *Mmp10*, *Mmp12*, and *Mmp13* from peritoneal macrophages left unstimulated (NT) or stimulated with AG (1 μg/ml) in the absence or presence of TAK1 inhibitor 5Z‐7‐OZ (1 μM) for 24 h.Immunoblots of cell supernatants to analyze secreted MMP9, MMP10, MMP12, and MMP13 by mouse peritoneal macrophages stimulated with AG (1 μg/ml) for indicated times in the absence or presence of TAK1 inhibitor 5Z‐7‐OZ (1 μM); GADPH of cell lysates served as the loading control.Immunoblots of cell supernatants to analyze secreted MMP9, MMP10, MMP12, and MMP13 by mouse peritoneal macrophages infected with H37Rv for indicated times (MOI = 5) in the absence or presence of TAK1 inhibitor 5Z‐7‐OZ (1 μM); GADPH of cell lysates served as the loading control.Immunoblots of cell lysates of HEK293T cells stimulated with AG (1 μg/ml) for the indicated time to analyze p‐ERK1/2 and p‐TAK1(T187). GADPH of cell lysates is shown as loading control.Immunoblots of lysates of HEK293T cells stimulated with AG (1 μg/ml) for 3 h after transfection of the indicated plasmids for 48 h. Data information: Data in (B) are means ± SD averaged from 3 independent experiments performed with technical triplicates and each symbol represents the mean of technical triplicates. Two‐way ANOVA followed by Dunnett's *post hoc* test were used for statistical analysis. ns, not significant; **P < 0.01; ****P < 0.0001. Source data are available online for this figure.

Given that AG causes lung damage through MMPs and hence acts as a detrimental virulence factor, we next set out to identify its receptor. To this end, we performed a surface plasmon resonance (SPR) assay (Olaru *et al,* 2015) to examine interactions of AG with galectins, which are cognates of the mammalian lectin family and bind to the β‐galactopyranoside‐containing carbohydrate moieties of glycoconjugates (Laaf *et al,* 2018). We obtained various galectins with high purity as demonstrated by Coomassie Brilliant Blue staining (Fig EV2A). Although the chemical structure and conformation of β‐galactofuranoside (five‐member ring) differ markedly from those of β‐galactopyranoside (six‐member ring) (Fig EV2B), the SPR assay demonstrated the strongest binding affinity of AG to tandem‐repeat galectin‐9 (*K*_D_ = 22.3 μM) (Fig 5A) as compared to other galectins including galectin‐1, −3, −7, −8, −14, and LGALSL (Fig EV2, EV3, EV4, EV5). Galectin‐9 contains two carbohydrate recognition domains (CRDs) (Tureci *et al,* 1997). By using purified CRDs (Fig 5B), SPR assays revealed that CRD2, but not CRD1 of galectin‐9, strongly interacted with AG (*K*_D_ = 53.8 nM) (Figs 5C and EV2J). Although the N terminus of CRD1 starts at amino acid 16, the β‐sandwich fold of CRD1 originally starts at Ser6 in the crystal structure (Nagae *et al,* 2008). We therefore further purified the N terminus of galectin‐9 including amino acid 1–146 to avoid the destruction of the β‐sandwich fold of CRD1 (Fig EV2K). As demonstrated by the SPR assay, galectin‐9 (1–146) did not bind AG (Fig EV2L), indicating that the weak sugar affinity of CRD1 was not due to misfolding because of invalid construct design. Intriguingly, binding of CRD2 with AG was ca. 3 orders of magnitude higher than that of full‐length galectin‐9 (Fig 5A and C), suggesting that AG‐galectin‐9 binding may involve autoinhibition. To further verify whether galectin‐9 binds to *Mtb* directly, we harvested *Mtb* from a log phase culture in the presence of Tween‐80 and incubated these bacteria with galectins followed by FACS analysis. Galectin‐9, but not galectin‐3 or galectin‐8, was shown to specifically bind to *Mtb* H37Rv (Fig 5D). Galectin‐9 is also bound to other AG‐containing mycobacteria including *M. bovis* BCG and *Mycobacterium smegmatis*, but not AG‐deficient bacteria such as *E. coli* (Fig 5E). The binding of galectin‐9 with wild‐type and MMAR_5356 or MMAR_5357 knockdown mutants of *M. marinum* was further detected by FACS. The data revealed that tetracycline‐mediated knockdown of both genes led to profound attenuation of galectin‐9 binding (Fig 5F). Therefore, our results suggest that galectin‐9 plays an important role in AG sensing.

### Galectin‐9 is essential for AG‐induced ERK‐mediated MMPs production

**Figure 6:**
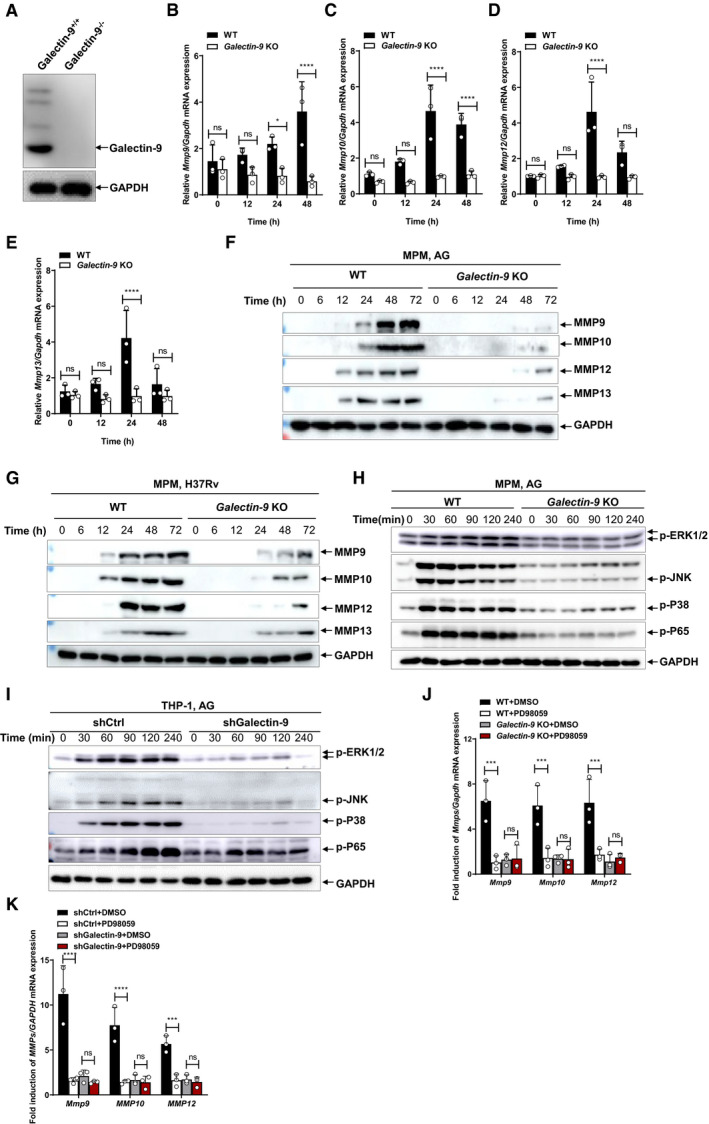
Galectin‐9 is essential for AG‐induced production of MMPs AImmunoblots of cell lysates were performed to analyze galectin‐9 by mouse peritoneal macrophages isolated from WT or *Galectin‐9* KO mice, and GADPH as a loading control.B–EqPCR analysis of *Mmps* including *Mmp9* (B), *Mmp10* (C), *Mmp12* (D), and *Mmp13* (E) from mouse peritoneal macrophages isolated from either wild‐type or *Galectin‐9* KO mice stimulated with AG (1 μg/ml) for 24 h.FImmunoblots of cell supernatants to analyze secreted MMP9, MMP10, MMP12, and MMP13 by mouse peritoneal macrophages isolated from WT or *Galectin‐9* KO mice stimulated with AG (1 μg/ml) for indicated times; GADPH of cell lysates served as the loading control.GImmunoblots of cell supernatants to analyze secreted MMP9, MMP10, MMP12, and MMP13 by mouse peritoneal macrophages isolated from WT or *Galectin‐9* KO mice infected with H37Rv for indicated times (MOI = 5); GADPH of cell lysates served as the loading control.HImmunoblot of lysates of peritoneal macrophages isolated from wild‐type and *Galectin‐9* KO mice stimulated with AG (1 μg/ml) for indicated times. Data are representative of *n* = 3 independent experiments.IImmunoblot of lysates of shCtrl and shGalectin‐9 THP‐1 cells stimulated with AG (1 μg/ml) for indicated times. Data are representative of *n* = 3 independent experiments.JqRT–PCR detection of *Mmp* transcripts including *Mmp9*, *Mmp10*, and *Mmp12* in wild‐type and *Galectin‐9* KO peritoneal macrophages stimulated with AG (1 μg/ml) for 24 h in the absence or presence of ERK inhibitor PD98059 (10 μM).KqRT–PCR detection of *MMP* transcripts including *Mmp9*, *Mmp10*, and *Mmp12* in shCtrl and shGalectin‐9 THP‐1 cells stimulated with AG (1 μg/ml) for 24 h in the absence or presence of ERK inhibitor PD98059 (10 μM). Data information: Data in (B to E, J, and K) are means ± SD averaged from 3 independent experiments performed with technical triplicates and each symbol represents the mean of technical triplicates. Two‐way ANOVA followed by Dunnett's *post hoc* test were used for statistical analysis. ns, not significant; *P < 0.05; ***P < 0.001 ****P < 0.0001. Source data are available online for this figure.

To determine the role of galectin‐9 in AG‐related functions, THP‐1 cells were stably transfected with shRNA specifically targeting *Galectin‐9* for knockdown of its expression (Fig EV3A). Specific knockdown of galectin‐9 by shRNA markedly reduced the mRNA levels of MMPs in AG‐treated THP‐1 cells (Fig EV3, EV4, EV5). To characterize the function of galectin‐9 in more depth, we generated *galectin‐9* knockout (KO) mice by CRISPR/Cas9‐mediated genome editing (Wang *et al,* 2013; Liu *et al,* 2017b) (Figs 6A and EV3E and F). The expression of MMPs in response to AG was markedly lower in macrophages isolated from *Galectin‐9* KO mice as compared to wild‐type (Fig 6B–E). Moreover, deletion of galectin‐9 blocked secretion of MMPs including MMP9, MMP10, MMP12, and MMP13 in mouse macrophages in response to AG stimulation (Fig 6F). Furthermore, the secretion of MMPs in galectin‐9 KO macrophages was reduced as compared to that in wild‐type macrophages infected with *Mtb* H37Rv (Fig 6G).

We next interrogated whether AG activates ERK to induce MMPs through galectin‐9. Knockdown or KO of galectin‐9 significantly reduced AG‐induced activation of corresponding signaling cascades such as MAPKs including ERK, p38, and JNK, as well as NF‐κB in both murine and human macrophages (Fig 6H and I), suggesting that AG activates MAPKs and NF‐κB signaling pathways through galectin‐9. Finally, AG‐induced MMP expression did not differ in either galectin‐9 knockdown or KO cells when ERK was inhibited by the specific inhibitor PD98059 (Fig 6J and K). Collectively, these data suggest an essential role of galectin‐9 in the activation of ERK signaling by AG to induce the expression of MMPs.

### Association of galectin‐9 with TAK1 is essential for activation of downstream events by AG

**Figure 7:**
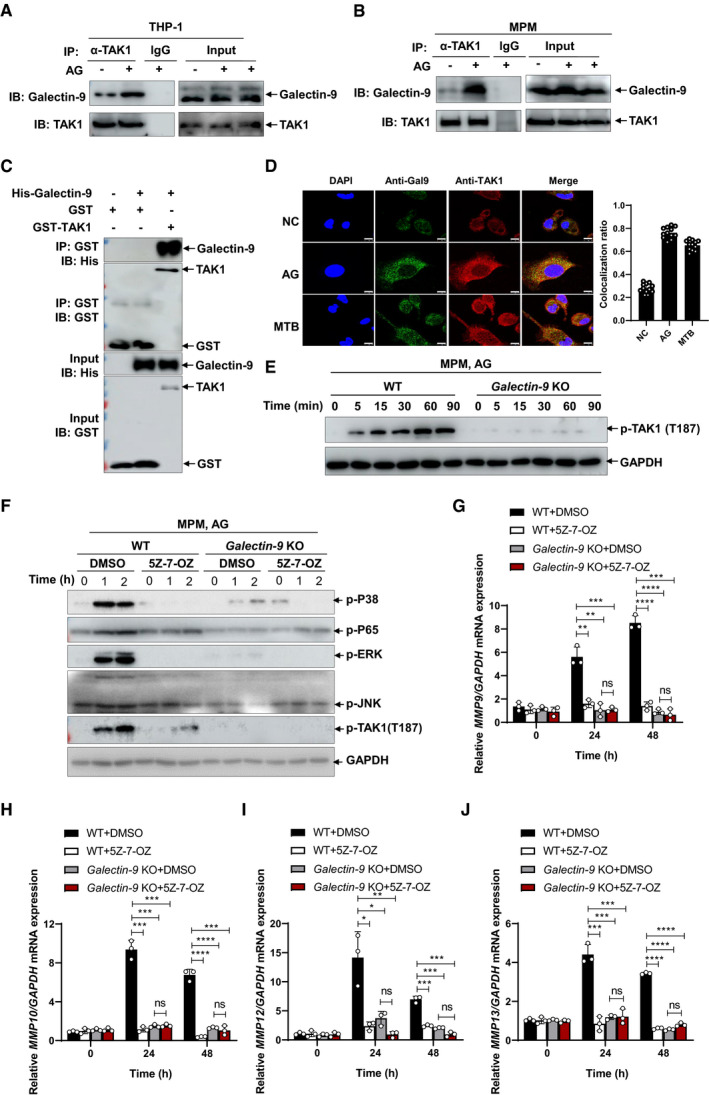
Galectin‐9 mediates TAK1 recruitment to induce production of MMPs A, BImmunoblots and immunoprecipitation of cell lysates to analyze endogenous interaction of galectin‐9 with TAK1 by human THP‐1 cells (A) or mouse peritoneal macrophages (B) left unstimulated or stimulated with AG (1 μg/ml) for 1 h.C
*In vitro* glutathione S‐transferase (GST) precipitation assay purified histidine (His)‐tagged Galectin‐9 (+) with GST alone or GST‐tagged TAK1.DConfocal microscopy of mouse peritoneal macrophages left untreated (NC) (upper row) or stimulated with AG (1 μg/ml) for 2 h (middle row) or infected with H37Rv for 3 h (MOI = 5) (bottom row), staining with anti‐Galectin‐9 and anti‐TAK1 antibody. DAPI, nuclei, blue. Scale bar, 5 μm. Data in the right graph show mean ± SD of *n* = 12 fields from three independent experiments. The symbols indicate the colocalization ratio of at least 10 cells in each field.EImmunoblots of cell lysates to analyze phosphorylated TAK1 by mouse peritoneal macrophages isolated from WT or *Galectin‐9* KO mice stimulated with AG (1 μg/ml) for indicated times; GADPH of cell lysates served as the loading control. Data are representative of at least *n* = 3 independent experiments.FImmunoblots of cell lysates of peritoneal macrophages isolated from WT or *Galectin‐9* KO mice stimulated with AG (1 μg/ml) in the absence or presence of TAK1 inhibitor 5Z‐7‐OZ (1 μM) for indicated times. Data are representative of *n* = 3 independent experiments.G–JqPCR analysis of *Mmps* including *Mmp9* (G), *Mmp10* (H), *Mmp12* (I), and *Mmp13* (J) from WT or *Galectin‐9* KO mouse peritoneal macrophages stimulated with AG (1 μg/ml) for 24 h in the absence or presence of TAK1 inhibitor 5Z‐7‐OZ (1 μM). Data information: Data in (G to J) are means ± SD averaged from 3 independent experiments performed with technical triplicates, and each symbol represents the mean of technical triplicates. Two‐way ANOVA followed by Dunnett's *post hoc* test were used for statistical analysis. ns, not significant; *P < 0.05; **P < 0.01; ***P < 0.001 ****P < 0.0001. Source data are available online for this figure.

To gain deeper insights into the mechanism underlying the nature of galectin‐9 activation of MAPK signaling and MMP expression, we assessed the interaction between galectin‐9 and MAPK signaling molecules. Galectin‐9 was found to interact with transforming growth factor β‐activated kinase 1 (TAK1) in HEK293T cells (Fig EV4A), which is consistent with recent reports that galectin‐9 associates with TAK1 in response to endomembrane damage (Jia *et al,* 2018; Jia *et al,* 2020). Upon treatment of AG, the interaction of endogenous TAK1 to galectin‐9 was increased in both THP‐1 cells and primary peritoneal macrophages (Fig 7A and B), indicative of a stimulus‐dependent interaction between galectin‐9 and TAK1. Moreover, an *in vitro* GST pull‐down assay revealed direct interaction of galectin‐9 with TAK1 (Fig 7C). Confocal microscopy revealed increased colocalization of galectin‐9 with TAK1 in response to AG stimulation or *Mtb* infection (Fig 7D). To further map the region of galectin‐9 which mediates its interaction with TAK1, we generated different galectin‐9 truncated constructs (Fig EV4B). Co‐IP experiments demonstrated that the CRD2 domain, but not the linker region or CRD1 of galectin‐9 is critical for its interaction with TAK1 (Fig EV4C). In combination with our finding that AG stimulation or *Mtb* infection enhances the interaction of galectin‐9 with TAK1, we propose that binding of AG to galectin‐9 CRD2 domain leads to conformational changes, which in turn enhanced recruitment of TAK1.

Since galectin‐9 interacts with TAK1, we next examined whether galectin‐9 regulates the activation of TAK1. Knockout of galectin‐9 abrogated AG‐induced phosphorylation of TAK1 on Thr 187 (Fig 7E), which is critical for its activation (Singhirunnusorn *et al,* 2005). Furthermore, inhibition of TAK1 by a selective inhibitor, 5Z‐7‐oxozeaenol (5Z‐7‐OZ) (Ninomiya‐Tsuji *et al,* 2003), impaired AG‐induced activation of MAPKs including ERK, JNK, and p38 as well as NF‐κB (Fig EV5A). However, impaired AG‐induced downstream signaling by TAK1 inhibitor was not observed in *galectin‐9* KO macrophages (Fig 7F). Our results suggest that galectin‐9 activates TAK1, triggering the downstream MAPK signaling. To clarify the relationship between the binding with AG and phosphorylation of TAK1, we analyzed whether the CRD2 of galectin‐9, which is critical for its interaction with AG and TAK1 (Figs 5C and EV4C), is sufficient for TAK1 activation. AG stimulation caused the phosphorylation of TAK1 and EKR in HEK23T cells (Fig EV5E). Overexpression of galectin‐9 enhanced AG‐induced TAK1 and ERK activation in HEK293T cells (Fig EV5F). Of note, overexpression of CRD2 alone, but not of CRD1 alone, induced the activation of TAK1 and ERK in HEK293T cells (Fig EV5F). This shows that the formation of the AG‐galectin‐9‐TAK1 complex is critical for the activation of TAK1 and downstream signaling.

We next examined the effect of galectin‐9/TAK1 signaling on MMPs expression. Inhibition of TAK1 by 5Z‐7‐OZ abrogated AG‐induced expression and secretion of MMPs in primary peritoneal macrophages (Fig EV5B and C). Moreover, the secretion of MMPs was also profoundly reduced in macrophages infected with *Mtb* H37Rv when the cells were treated with TAK1 inhibitor 5Z‐7‐OZ (Fig EV5D). However, the blockade of MMPs expression by TAK1 inhibition was not detected in those *galectin‐9* KO counterparts (Fig 7G–J). We conclude that galectin‐9 activates TAK1 to induce the expression of MMPs through MAPK signaling.

### Galectin‐9 is essential for the *in vivo* activity of AG

**Figure 8:**
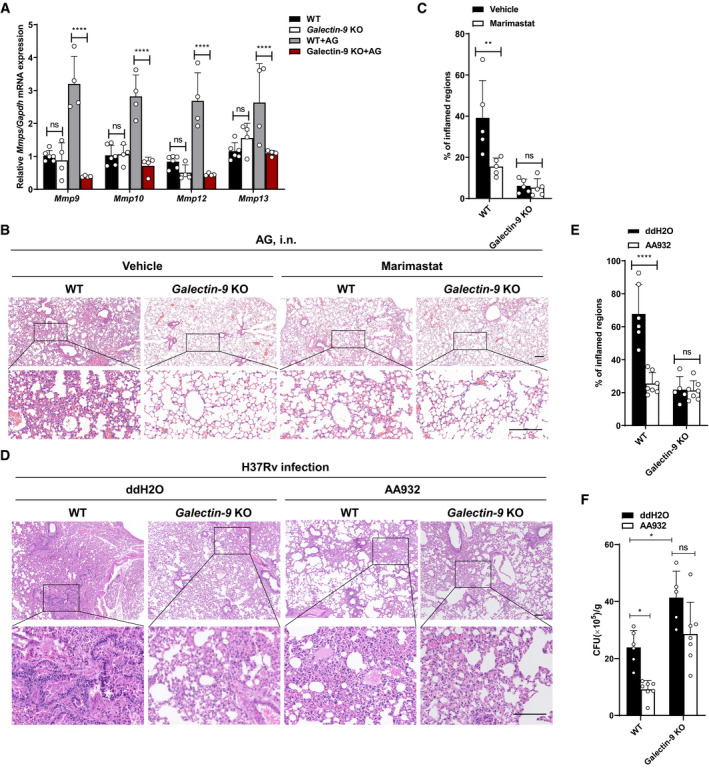
Galectin‐9 is essential for the *in vivo* effects of AG AqPCR analysis of *Mmps* including *Mmp9*, *Mmp10*, *Mmp12*, and *Mmp13* from the lungs of WT or *Galectin‐9* KO mice at 3 days post‐intraperitoneal administration of AG (100 μg).B, CWT or *Galectin‐9* KO mice were intraperitoneally treated with AG for 3 days in the absence or presence of the MMP inhibitor marimastat (10 mg/kg) given intraperitoneally prior to AG stimulation. Lung sections stained with H&E (B) and quantification of lung lesion burden from H&E‐stained sections (C).D, EWT or *Galectin‐9* KO mice were intranasally infected with H37Rv for 4 weeks in absence or presence of intranasally administrated AG aptamers (1 μg) once at a 1‐week interval. Lung sections stained with H&E (D) and quantification of lung lesion burden from H&E‐stained sections (E).FCFU quantification of the bacterial titers of lung tissue homogenates from WT or *Galectin‐9* KO mice intranasally infected with H37Rv for 4 weeks in the absence or presence of intranasally administrated AG aptamers (1 μg) once at a 1‐week interval. Data information: Data in (B and D) are representative of *n* = 3 independent experiments. Data in (A, C, E, F) are means ± SD of the indicated number of mice from 1 of *n* = 3 independent experiments and each symbol represents data from 1 mouse. Two‐way ANOVA followed by Dunnett's *post hoc* test were used for statistical analysis. ns, not significant; *P < 0.05; **P < 0.01; ****P < 0.0001. Scale bar, 200 μm.

To characterize the functional consequences of AG sensing by galectin‐9 *in vivo*, we treated wild‐type or *Galectin‐9* KO mice with AG. The deletion of galectin‐9 dramatically reduced the abundances of MMPs transcripts in the lungs of mice receiving intraperitoneal administration of AG (Fig 8A) and consistently, in the lungs of *Galectin‐9* KO mice treated with AG‐ameliorated pathological damage, as compared to controls (Fig 8B and C). Furthermore, inhibition of MMPs by marimastat ameliorated pulmonary damage of wild‐type mice but not of galectin‐9 KO mice (Fig 8B and C). We conclude that AG causes pulmonary injury through galectin‐9‐mediated MMPs expression.

To further clarify the physiological relevance of the AG‐galectin‐9 axis in natural infection, we infected wild‐type or *galectin‐9* KO mice with *Mtb* in the absence or presence of AG aptamer, and analyzed tissue damage and mycobacterial growth in the lung. Deletion of *galectin‐9* markedly reduced pathological impairments in the lung of *Mtb*‐infected mice, and treatment with the AG aptamer alleviated the pathologic impairments in the lung of *Mtb*‐infected WT mice, but not of *Mtb*‐infected *galectin‐9* KO mice (Fig 8D and E). The findings indicate that AG induces lung tissue damage through galectin‐9. Consistent with previous reports on anti‐TB effects of galectin‐9 (Jayaraman *et al,* 2010; Sada‐Ovalle *et al,* 2012; Chavez‐Galan *et al,* 2017; Jia *et al,* 2018), we observed that the KO of galectin‐9 moderately enhanced mycobacterial growth in the lung (Fig 8F). However, administration of the AG aptamer significantly reduced the bacterial load in the lung of *Mtb*‐infected WT mice, but not of *Mtb*‐infected *galectin‐9* KO mice (Fig 8F), indicating that AG promotes the growth of *Mtb* through galectin‐9. Together, our results suggest that the AG‐galectin‐9 axis aggravates the pathogenesis of murine *M. tuberculosis* infection.

## Discussion

Sensing of bacterial molecular components is critical for the host to generate protective or pathogenic immune responses (Orme *et al,* 2015; Dorhoi & Kaufmann, 2016). One striking characteristic feature of *Mtb* is its unusual AG‐containing cell wall which is a frequent structural and bio‐synthetical target for anti‐TB drug development (Jankute *et al,* 2015; Grzegorzewicz *et al,* 2016). Intriguingly, we demonstrate that mycobacterial AG interacts with galectin‐9. Interaction of AG with galectin‐9 activated TAK1‐ERK MAP kinase to induce the expression of MMPs. Moreover, KO of *galectin‐9* or inhibition of MMPs blocked AG‐induced lung injury. Thus, AG qualifies as a virulence factor of *Mtb* that is recognized by galectin‐9, providing a molecular mechanism for the functions of AG and galectin‐9 in TB (Appendix Fig S4).

*Mtb* possesses an unusual cell wall: Its inner layer is comprised of peptidoglycan, the middle layer of a highly branched AG, and the outer layer of long‐chain mycolic acids (Jankute *et al,* 2015). Both peptidoglycans and mycolic acids are well known as important PAMPs that trigger a cascade of innate immune responses (Korf *et al,* 2005; Verschoor *et al,* 2012; Wolf *et al,* 2016; Wolf & Underhill, 2018; Lu *et al,* 2019; Tahiri *et al,* 2020; Bastos *et al,* 2021). AG is an essential component of the hydrophobic and highly impermeable cell wall of *Mtb*; however, its biological function remains elusive. It has been previously reported that AG binds to NKp44 on the surface of natural killer (NK) cells, but fails to induce their activation (Esin *et al,* 2013). Moreover, the presence of a galactosamine substituent in the AG of *Mtb* has been found to abrogate the full maturation of human peripheral blood monocyte‐derived DCs (Wheat *et al,* 2015). Here, we demonstrate that *in vivo* administration of AG causes pathological impairments in the lung, while AG‐specific aptamers alleviated *Mtb*‐induced lung injury and moderately extended survival of *Mtb*‐infected SCID mice or *M. marinum*‐infected zebrafish. Given that TB is primarily a pulmonary disease, and that cavitation of the lung promotes the efficient spread of the pathogen, our findings identify AG as a previously unrecognized molecular component of the pathogenic signature of *Mtb*. Considering that the blockade of AG by aptamers only showed minor effects on *Mtb* survival in the lung of infected mice, AG may aggravate the pathology of *Mtb* infection largely by modulation of lung lesions.

*Mtb* infection leads to increased secretion of matrix‐degrading enzymes, MMPs, which are closely associated with the severity of lung injury in pulmonary TB (Walker *et al,* 2012; Ordonez *et al,* 2016). Deep sequencing indicates that AG strongly induces the expression of MMPs, and, reciprocally, that AG‐specific aptamers or genetic knockdown of AG synthesis genes markedly reduce MMP induction both *in vitro* and *in vivo* by *Mtb*, BCG, or *M. marinum*. These findings point to AG as a major mycobacterial inducer of MMPs. Furthermore, treatment with the MMP inhibitor marimastat (Skipper *et al,* 2009) profoundly reduced the pulmonary pathology of mice challenged with AG. Therefore, AG qualifies as a critical virulence factor of *Mtb* which contributes to lung injury through MMPs production in the pathogenesis of TB. It remains to be determined whether AG regulates the formation of granulomatous cavities (Walker *et al,* 2012; Ordonez *et al,* 2016).

It is well established that *Mtb* infection causes upregulation of MMPs in monocytic cells and mouse lungs (Rivera‐Marrero *et al,* 2002; Rand *et al,* 2009; Lou *et al,* 2017; Sabir *et al,* 2019). The cell wall glycolipid lipoarabinomannan (LAM) induces expression of MMP9 in THP‐1 cells through mannose receptor‐mediated activation of p38, and transcriptional activation by activator protein‐1 (AP‐1) (Rivera‐Marrero *et al,* 2002). ESAT‐6, a virulence factor of *Mtb*, drives ERK and p38 MAPK‐dependent expression of MMP‐10 (Brilha *et al,* 2017). Here, we demonstrate that AG upregulates the expression and secretion of MMPs through activation of the TAK1‐ERK signaling pathway. TAK1 plays a key role in cellular responses to a variety of stimuli by triggering the activation of downstream effectors (Ajibade *et al,* 2013). Our recent work demonstrated that TAK1 serves as an important trigger for protective immunity against *Mtb* infection by inducing both Il6 and Il12b (Zheng, *et al,* 2018a). Moreover, we demonstrated previously that TAK1 is activated by *Mtb* infection through oxidation by *Mtb*‐secreted protein MPT53 (Wang *et al,* 2019). Our present work demonstrates that the AG‐galectin‐9 axis is critical for the activation of TAK1, adding a novel layer to the complexity in the regulation of TAK1. Moreover, we demonstrate that the galectin‐9‐TAK1 pathway is exploited by AG as an *Mtb* virulence factor that causes lung injury by inducing MMPs, emphasizing a detrimental role of TAK1 in TB.

Accumulating evidence has revealed an immunomodulatory role of galectin‐9 in multiple immune cell types including B cells, T cells, NK cells, eosinophils, mast cells, and mononuclear phagocytes (Rabinovich & Toscano, 2009; Wiersma *et al,* 2013; Cao *et al,* 2018; Giovannone *et al,* 2018). Galectin‐9 has been shown to be secreted or to exert its function in the nucleus or cytoplasm (Kanwar *et al,* 1997; Heusschen *et al,* 2013). Increasing evidence suggests that galectins are important for the formation of signalosomes and subsequent signaling activation (Laderach *et al,* 2010). Recently, galectin‐9 has also been found to sense lysosomal damage signaling and to form a complex with TAK1(Jia *et al,* 2018; Jia *et al,* 2019; Jia *et al,* 2020). By confocal microscopy assay, we observed colocalization of galectin‐9 with TAK1 in the cytosol in response to AG stimulation or *Mtb* infection. We conclude that galectin‐9 is a cytosolic receptor of AG. Moreover, coimmunoprecipitation assays with truncated mutants of galectin‐9 demonstrated that CRD2, but not CRD1 or the linker region of galectin‐9 mediated its interaction with TAK1. In combination with the finding that AG stimulation or *Mtb* infection enhanced the interaction of galectin‐9 with TAK1, we propose that binding of AG to galectin‐9 CRD2 domain causes conformational changes of CRD2, which in turn enhances recruitment of TAK1 and formation of the galectin‐9/TAK1 signaling complex. Hence, we consider TAK1 as a central kinase of signal transduction from galectin‐9 to downstream MAPK activation and MMP production.

Our data reveal a direct interaction of galectin‐9 with AG, and binding of galectin‐9 to AG‐containing but not to AG‐deficient mycobacteria. Importantly, the knockdown of genes involved in AG biosynthesis including *MMAR_5356* and *MMAR_5357* markedly reduced galectin‐9 binding. Hence, galectin‐9 plays a critical role in mycobacterial AG sensing. Of note, AG is hidden and surrounded by the outermost mycomembrane layer in mycobacteria. Hence, the detergent Tween‐80 present in the culture medium could disrupt the outer layer of the mycobacterial cell wall and expose AG to the galectin‐9 binding. During natural infection, the harsh environment in the phagosome and lysosome could lead to AG shedding from the *Mtb* cell wall and leakage of AG into the cytosol through the damaged membrane. This process could facilitate the engagement of galectin‐9 and activation of TAK1 in the cytosol. Moreover, it has been reported that during replication, bacteria can expose the newly synthesized AG (Meniche *et al,* 2014), which in turn could be recognized by galectin‐9. Therefore, we consider galectin‐9 as a cytosolic receptor for AG released from invading mycobacteria in the phagosome/lysosome or from replicating mycobacteria in the cytosol during intracellular *Mtb* infection (van der Wel *et al,* 2007; Houben *et al,* 2012; Simeone *et al,* 2015; Chai *et al,* 2020). Mycolic acids have been found to form an integral component of the mycolyl‐arabinogalactan‐peptidoglycan (mAGP) complex which is bound galectin‐3 (Liu *et al,* 1996; Barboni *et al,* 2005). Yet, evidence for the direct interaction of galectin with AG is missing. In our study, we took advantage of the first complete synthesis of a mycobacterial AG composed of 92 mono‐saccharide units (Wu *et al,* 2017), and harnessed the chemically synthesized AG for in‐depth evaluation of its biological functions. Our work demonstrated that AG interacted with galectin‐9 directly, which for the first time provides solid evidence for the direct interaction of AG with a host lectin galectin‐9. Our finding, therefore, emphasizes the impressively broad range of sensors for mycobacteria (Killick *et al,* 2013; Moura‐Alves *et al,* 2014; Liu *et al,* 2017a).

Of note, galectins interact with the β‐galactopyranoside‐containing carbohydrate moieties of glycoconjugates (Laaf *et al,* 2018), and the OH4 and OH6 of β‐galactopyranoside are critical for its interaction with galectins (Chan *et al,* 2018). However, AG harbors β‐galactofuranoside (five‐member ring), the chemical structure and conformation of which differ markedly from those of β‐galactopyranoside (six‐member ring). β‐galactofuranose lacks both OH4 and OH6 at the equivalent positions, indicating that β‐galactofuranoside cannot mediate the interaction of AG with galectin‐9. Of note, chemically synthesized AG used in this study is a highly complex polysaccharide composed of 92 sugar units, which not only contains β‐galactofuranose residues, but also numerous α‐ and β‐arabinofuranose residues. Moreover, the tertiary structure of AG could be responsible for its interaction with galectin‐9. Hence, the precise mechanisms underlying interactions of AG with galectin‐9 await the crystal structure analysis of the AG‐galectin‐9 complex.

Consistent with a previous report that galectin‐9 KO slightly enhances mycobacterial survival in macrophages (Jayaraman *et al,* 2010), our data demonstrate that deletion of *galectin‐9* resulted in a moderate increase in bacterial burden in the lung. However, we also found that deletion of galectin‐9 ameliorated inflammation as shown by H&E staining. Therefore, it is reasonable to speculate that ameliorated inflammation in galectin‐9 KO mice results in a failure to control mycobacterial replication. Consistent with this, multiple pro‐inflammatory cytokines such as IL‐6 and IL‐1β have been shown to control mycobacterial growth (Ladel *et al,* 1997; Mayer‐Barber *et al,* 2014; Sousa *et al,* 2020; Wang *et al,* 2020). Alternatively, galectin‐9 may regulate both inflammation and bactericidal growth directly in an uncoupled manner. Sensing of AG by galectin‐9 as a prerequisite for lung injury may separate this pathway from galectin‐9‐mediated antimicrobial immunity (Jayaraman *et al,* 2010; Sada‐Ovalle *et al,* 2012; Chavez‐Galan *et al,* 2017). Accordingly, although galectin‐9 moderately inhibited *Mtb* survival in the lung, engagement of galectin‐9 by AG slightly increased pulmonary *Mtb* burden as demonstrated by AG aptamer treatment in WT and *galectin‐9* KO mice infected with *Mtb*. Galectin‐9 has been shown to control phagosome biology (Jia *et al,* 2018; Jia *et al,* 2020) and also to control *Mtb* through caspase‐1‐dependent IL‐1β production by ligation of T cell Ig and mucin domain 3 (Tim3) (Jayaraman *et al,* 2010; Sada‐Ovalle *et al,* 2012; Chavez‐Galan *et al,* 2017). Although galectin‐9‐mediated TAK1 activation apparently represents the converging signaling event in response to both endomembrane damage and AG stimulation, our finding that sensing of AG by galectin‐9 is a prerequisite for lung injury separates this pathway from galectin‐9‐mediated antimicrobial immunity (Jayaraman *et al,* 2010; Sada‐Ovalle *et al,* 2012; Chavez‐Galan *et al,* 2017; Jia *et al,* 2018). These findings emphasize the versatile role of galectin‐9 in TB. Hence, our study can form the basis for the rational development of a novel type of host‐directed therapy targeting AG‐galectin‐9 interactions in adjunct to canonical drug therapy in TB.

## Materials and Methods

### Reagents

p38 MAPK inhibitor SB203580, MEK/ERK inhibitor PD98059, NF‐κB inhibitor PDTC, JNK inhibitor SP600125, and pan‐MMP inhibitor Marimastat were obtained from MedChem Express. TAK1 inhibitor 5Z‐7‐oxozeaenol was purchased from Sigma‐Aldrich. The following antibodies were used: phospho‐p44/42 MAPK (Erk1/2) (Thr202/Tyr204) (#4370), Phospho‐p38 MAPK (Thr180/Tyr182) (D3F9) (#4511), Phospho‐SAPK/JNK (Thr183/Tyr185) (81E11) (#4668), phospho‐NF‐κB p65 (Ser536)(93H1) (#3033), Phospho‐TAK1 (Thr187) Antibody(#4536), anti‐galectin‐9 antibody (54330), anti–β‐actin (#4970), Anti‐rabbit IgG (H + L), F(ab')_2_ Fragment (Alexa Fluor^®^ 488 Conjugate) (#4412), Anti‐mouse IgG (H + L), F(ab')_2_ Fragment (Alexa Fluor^®^ 555 Conjugate) (#4409), horseradish peroxidase (HRP)‐conjugated anti‐rabbit or anti‐mouse IgG (all from Cell Signaling Technology, Danvers, MA). Anti‐galectin‐3 antibody [EP2775Y] (ab76245), anti‐galectin‐8 antibody [EPR4857] (ab109519) and anti‐galectin‐9 antibody [EPR22214] (ab227046) were purchased from Abcam for FACS analysis. APC‐conjugated F(ab')2‐Goat anti‐Rabbit IgG (H + L) Cross‐Adsorbed Secondary Antibody (Invitrogen™) was purchased from Thermo Fisher Scientific. Anti‐MMP2 (GB11130), Anti‐MMP9 (GB12132‐1), Anti‐MMP13 (GB11247‐1) was purchased from Servicebio (Wuhan, China), Anti‐MMP10 (A3033) and Anti‐MMP12 (A1709) was purchased from Abclonal (Wuhan, China), and Anti‐TAK1 (sc‐166562) was purchased from Santa Cruz Biotechnology (Texas, USA). Recombinant galectin‐1 (C285), galectin‐3 (C846), galectin‐7 (C069), galectin‐8 (C081), galectin‐9 (C808), galectin‐14 (C803), and LGALSL (Ce89) were purchased from Novoprotein (Shanghai, China).

### Cell culture

THP‐1 cells (human monocytic cell line, ATCC TIB‐202) were cultured in RPMI 1640 (GIBCO) supplemented with 10% (v/v) heat‐inactivated fetal bovine serum (Sigma‐Aldrich, F0804), 1 mM sodium pyruvate (Gibco, 11360070), 2 mM l‐glutamine (Gibco, 25030081), 10 mM HEPES buffer (Gibco, 15630080), pH 7.2‐7.5, and 50 µM 2‐mercaptoethanol (Gibco, 31350010). THP‐1 cells were differentiated into macrophages by treatment with 200 nM PMA (Sigma‐Aldrich) for 24 h and then left rested for another 48 h for differentiation followed by subsequent experiments. Cells were maintained at 37°C in 5% CO_2_. A LookOut Mycoplasma PCR Detection Kit (MP0035, Sigma‐Aldrich) was applied for screening of mycoplasma contamination. All cells included in the study were confirmed to be free of mycoplasma.

### Bacterial strains and culturing conditions

The mycobacterial strains, *Mtb* H37Rv, *M. bovis BCG*, and *M. smegmatis* mc^2^155, were grown in Middlebrook 7H9 broth (Becton Dickinson, Cockeysville, MD) with 0.05% Tween‐80 and 10% oleic acid‐albumin‐dextrose‐catalase (OADC) (Becton Dickinson, Sparks, MD). *Mycobacterium* *marinum* M strain was cultured in Middlebrook 7H9 broth containing 0.2% glycerol, 10% ADC (Becton Dickinson, Sparks, MD) or on 7H10 agar supplemented with 0.5% glycerol and 10% OADC (Becton Dickinson, Sparks, MD) at 30°C under aerobic conditions. *E. coli* DH5α was grown in LB medium.

### Animal model

*Galectin‐9* KO mice were generated by CRISPR/Cas9 method (Wang *et al,* 2013; Liu *et al,* 2017b) following the procedures described previously (Liu *et al,* 2018a; Zheng *et al,* 2018b). The *Galectin‐9* sgRNA were designed by targeting the exon 2 of galectin‐9 and the sequences were sgRNA‐1: GAACTTAGGGTCCCTCGTAG, sgRNA‐2: CCCTTTACTGGACCAATCCA, and sgRNA‐3: GTCAACGGTGCTAAGATGGC. C57BL/6n female mice (7–8 weeks old) were used as embryo donors. C57BL/6n female mice were superovulated by intraperitoneal injection of pregnant mare serum gonadotropin (PMSG) and human chorionic gonadotrophin (hCG) and then mated to C57BL/6n male mice. The fertilized embryos (zygotes) were collected from oviducts. Cas9 mRNA (100 ng/µl) and sgRNA (25 ng/µl) targeting *Galectin‐9* were mixed and injected into the cytoplasm of fertilized eggs with both pronuclei visible in CZB (Chatot‐Ziomek‐Bavister) medium. The injected zygotes were then cultured in Quinn’s Advantage cleavage medium (In‐Vitro Fertilization, Inc.) for about 24 h, and every 18–20 2‐cell stage embryos were transferred into the oviduct of a pseudopregnant ICR female mouse at 0.5 days post‐coitus. To determine the nucleotide sequences of mutated alleles, DNA sequencing of F0 mice was performed after TA cloning into plasmid pMD19T (TAKARA). To obtain F1 *Galectin‐9* KO mice, F0 mice were crossed with C57BL/6n, and newborn generations were genotyped by Sanger sequencing. Genomic DNA was extracted from tail tips, and the PCR‐based genotyping was performed. The genotyping primer sequences for genotyping are *Galectin‐9*_F: TCACCTCCTACAGACTGGGG and *Galectin‐9*_R: GGACCTTCTCTGCAACACCA.

Female C57BL/6n mice (6–8 weeks old) were purchased from Shanghai Laboratory Animal Center (Shanghai, China). All mice were kept under specific pathogen‐free (SPF) conditions at the Laboratory Animal Center of Tongji University. All experiments were approved by Tongji University School of Medicine Animal Care and Use Committee and were conducted following the National Institutes of Health U.S.A (NIH) Guidelines for the Care and Use of Laboratory Animals.

### Mouse macrophage isolation and infection

Peritoneal macrophages were isolated as described (Kong *et al,* 2009). Briefly, mice received intraperitoneally 2.0 ml of 4% Brewer’s thioglycollate medium (B2551, Sigma‐Aldrich). After 3 days, peritoneal exudate cells were collected from euthanized animals using 10 ml cold PBS. Subsequently, cells were plated in 12‐well plates at 10^6^ cells/well in RPMI 1640 supplemented with 10% FBS plus penicillin/streptomycin and were incubated at 37°C in 5% CO_2_ for 2 h. Cultures were washed three times with PBS to remove non‐adherent cells, and the remaining adherent monolayer cells were used as primary peritoneal macrophages. Before infection, *Mtb* organisms were suspended in a complete medium without antibiotics. Peritoneal macrophages were infected with *Mtb* at an MOI of 5.

### Generation of stable cell lines

Lentivirus‐mediated knockdown of specific genes was performed as described previously (Liu *et al,* 2018b). HEK293T cells were transfected using Lipofectamine 2000 (Invitrogen, Carlsbad, CA) with pSPAX2, pMD2.G, and pLKO.1 harboring shRNA targeting human *LGALS9* (GTACCGGCCCTCCTCTCTGACCTTTAACCTCGAGGTTAAAGGTCAGAGAGGAGGGTTTTTTG, TRCN0000381715, Sigma‐Aldrich) or scrambled shRNA. Viral supernatants were harvested 48 h after transfection and were concentrated using an SW‐28 rotor. THP‐1 cells were infected with concentrated virus in the presence of polybrene (10 μg/ml). THP‐1 cells stably transfected with shRNA targeting *LGALS9* (shGalectin‐9) or with scramble shRNA (shCtrl) were isolated by puromycin selection (5 μg/ml).

### Chemical synthesis of AG

Full‐length AG composed of 92 mono‐saccharide units was synthesized following the reported procedure (Wu *et al,* 2017) and was used throughout the functional study.

### Purification of recombinant His‐tagged CRDs of galectin‐9

Truncated human galectin‐9 including galectin‐9 (1–146), CRD1 (16–146), and CRD2 (207–355) cDNA was subcloned into a pET28a vector and BL21 (DE3)‐competent *E. coli* bacteria were then transfected with these constructs. Bacteria were grown in Luria‐Bertani (LB) liquid medium to an optical density (OD) at 600 nm (OD_600_) of approximately 0.8. Subsequently, cells were induced with isopropyl β‐D‐1‐thiogalactopyranoside (IPTG, 0.1 mM) overnight at 16°C. Recombinant CRD1 and CRD2 were purified from bacterial lysates using a (Ni)‐chelating Sepharose Fast Flow (SFF) column (GE Healthcare, Little Chalfont, UK). The concentration of galectin‐9 (1–146), CRD1, and CRD2 protein were measured with a Pierce BCA Protein Assay Kit (Thermo Fisher Scientific).

### Surface plasmon resonance (SPR)

The interaction of galectins with AG was detected by OpenSPRTM (Nicoya Lifesciences, Waterloo, Canada). Briefly, galectins proteins including galectin‐1, −3, −7, −8, −9, −14, and LGALSL as well as galectin‐9 (1–146), CRD1 and CRD2 of galectin‐9 were fixed on the COOH sensor chip by capture‐coupling, then AG at indicated concentrations was injected sequentially into the chamber in PBS at 25°C. The binding time and disassociation time were both 240 s with the flow rate of 20 μl/min. The chip was regenerated with 0.02% SDS. A one‐to‐one diffusion corrected model was fitted to the wavelength shifts corresponding to the varied glycan concentration. The kinetic constants, including the association constant (ka), dissociation constant (kd), and affinity (KD, *K*_D_ = kd/ka), were analyzed with TraceDrawer software (Ridgeview Instruments AB, Sweden).

### *MMAR_5356* and *MMAR_5357* knockdown *M. marinum*

*MMAR_5356* and *MMAR_5357* knockdown strains are derivatives of *M. marinum* Aronson (ATCC: BAA‐535; M strain) generated following published protocols (Rock *et al,* 2017). The recombinant plasmids encompassed *MMAR_5356* and *MMAR_5357* sgRNA (5′‐gggctgttcgtccgcgctga‐3′ or 5′‐ggcgccccgctacgcagtgg‐3, respectively) with plasmids PLJR962 in *Bsm*B1 restriction site was constructed by homologous recombination. Primers for fragments of *MMAR_5356* are MMAR_5536‐1‐F: 5′‐gggagggctgttcgtccgcgctgacgtctcggtttttgtactcgaaag‐3′ and MMAR_5536‐1‐R: 5′‐accaggtccgacatatcgatac‐3′; MMAR_5536‐2‐F: 5′‐ tcagcgcggacgaacagccctcccagattatatctatcactgata‐3′ and MMAR_5536‐2‐R: 5′‐gtatcgatatgtcggacctggt‐3′. Primers for fragments of *MMAR_5357* are MMAR_5537‐F: 5′‐gggaggcgccccgctacgcagtggcgtctcggtttttgtactcgaaag‐3′ and MMAR_5537‐R: 5′‐accaggtccgacatatcgatac‐3′. Fragments of genes were amplified by polymerase chain reaction (PCR) using corresponding primers and linked by homologous recombinase (Vazyme Biotech). The ligated products were transformed into competent *E. coli* DH5α and screened with kanamycin to obtain recombinants. Recombinant plasmids of the correct sequence were then electroporated into *M. marinum* Aronson competent cells. After resuscitation in 7H9 broth (Difco) for 7 h, recombinant mycobacterial strains were further screened with 7H10 broth (Difco) containing 25 μg/ml Kanamycin.

Positive clones were grown to log phase in 7H9 broth containing 25 μg/ml Kanamycin, followed by dilution in 7H9 broth with 100 ng/ml ATc (anhydrotetracycline) to 0.1 OD_600_ (optical density at 600 nm). ATc binds to TetR operon to induce CRISPRi. 0.8–1.0 OD_600_ equivalents of mycobacteria were harvested for total RNA isolation by TRIzol (Thermo Fisher). After removing the residual genomic DNA with DNase, RNA was reversely transcribed into cDNA (TAKARA) for quantitative real‐time PCR (qRT–PCR) using SYBR Green Supermix (Toyobo). The primers are sigA‐F: 5′‐tgatcgtgcgaaaaaccacc‐3′, sigA‐R: 5′‐aacttttcgaccgcacggat‐3′; MMAR_5356‐F: 5′‐gtttctggtgttggggcttt‐3′, MMAR_5356‐R: 5′‐ttggctcagcttgaacatcg‐3′; MMAR_5357‐F: 5′‐ggaggataggctcgggaatc‐3′, MMAR_5357‐R: 5′‐actggacgcaatcattacgc‐3′. Quantification Cycle Method (CT) was normalized to the housekeeping sigA transcript and quantified by the ΔΔCt method. All samples were three technical replicates.

### FACS for analyzing interactions of galectin with bacteria

Bacteria including *Mtb* H37Rv, *M. bovis* BCG, *M. smegmatis* mc^2^155, wide‐type *M. marinum*, *MMAR‐5356*, and *MMAR‐5357* knockdown *M. marinum* as well as *E. coli* DH5α at the order of 10^8^ were fixed with 4% PFA for 30 min followed by incubation with indicated galectin protein for 30 min at RT. The bacteria were washed with PBS 3 times followed by incubation with anti‐galectin antibodies for 1 h at RT. Subsequently, the bacteria were further washed and incubated with APC‐anti‐rabbit antibodies for 30 min at RT. The washed bacteria were then analyzed by FACS analysis (BD accuri C6, BD). FlowJo 10.5.3 (Ashland, OR) was applied for the FACS data analysis.

### System evolution of aptamers against AG by SELEX selection

Aptamers with an affinity for AG were selected by the SELEX method (Tuerk & Gold, 1990; Bock *et al,* 1992). The DNA library included a 35‐nucleotide random region as well as a 23‐nucleotide forward primer, which was identical to the 5′ flanking sequence of the library template (5ʹ‐GGGAGCTCAGAATAAACGCTCAA‐3ʹ), and a 20 nucleotide reverse primer, which was complementary to the 3′ flanking sequence of the library template (5ʹ‐GATCCGGGCCTCATGTCGAA‐3ʹ). The random single‐stranded DNA library and all primers were synthesized and purified by high‐performance liquid chromatography at the Shanghai Sangon Biological Engineering Technology & Services Co., Ltd. (Shanghai, China). ssDNA aptamers with high affinity against AG were screened as previously described (Qin *et al,* 2009; Qin *et al,* 2014; Aimaiti *et al,* 2015). 96‐well enzyme‐linked immunosorbent assay (ELISA) plates were coated with AG (in 0.1 M NaHCO_3_ buffer, pH 9.4) by overnight incubation at 4°C. Control wells were included by leaving wells uncoated (blank). The wells were then rinsed 4 times each with washing buffer (PBS containing 0.05% Tween 20, pH 7.4; PBST) and incubated for 1 h at RT with 200 μl of blocking buffer (PBS containing 3% bovine serum albumin (BSA) and 0.05% Tween 20, pH 7.2). The ssDNA pools were denatured by heating at 94°C for 5 min in SHCMK‐binding buffer (20 mM Hepes, pH 7.35, 120 mM NaCl, 5 mM KCl, 1 mM CaCl_2_, and 1 mM MgCl_2_) and then cooled to room temperature for 15 min. The ssDNA library was first added to control wells and incubated at 37°C, in order to screen out ssDNA‐targeting BSA. The unbound ssDNA was then removed and placed in the AG‐coated wells for incubation at 37°C. Unbound ssDNA sequences were removed by six rinses with washing buffer (SHCMK supplemented with 0.05% Tween 20; SHCMKT). Then, the AG‐bound ssDNA was recovered by incubation in elution buffer (20 mM Tris–HCl, 4 M guanidinium isothiocyanate, and 1 mM DTT, pH 8.3) at 80°C for 10 min. The eluates were mixed with phenol‐chloroform and centrifuged at 12,000 *g* for 5 min at 4°C. The resulting supernatants were mixed with dehydrated alcohol and NaAc (3 M, pH 5.2) overnight at −20°C, and followed by centrifugation at 12,000g for 20 min at 4°C. The supernatants were discarded, and the pellets were resuspended in 75% alcohol and centrifuged for 10 min. The precipitate was then dissolved in 30 μL TE buffer (pH 8.0) and applied as the DNA library for the next round of screening.

After 10 rounds of aptamer selection, PCR products from the last five rounds were purified using the TIANgel Midi Purification Kit (TIANgen Biotech Co., Ltd, Beijing, China) and cloned into a pMD18‐T vector by using a TA cloning kit (TaKaRa, Dalian, China). After transformation into *E. coli* DH5α cells and growth of the bacteria, random individual bacterial clones were picked from each of the five aptamer selection rounds for sequencing (Sangon, Shanghai, China). Individual aptamer sequence and secondary structure were identified using DNAMAN version 6.0 (Lynnon, Quebec, Canada).

### Mouse challenge with AG

Female mice (6–8 weeks old) were divided randomly into cages upon arrival and were challenged by intraperitoneal or intravenous administration of AG. For intraperitoneal administration, indicated amounts of AG were directly injected into the peritoneum. For intravenous administration, indicated amounts of AG or AG water/oil/water (w/o/w) emulsion were injected intravenously. The w/o/w emulsion containing AG was prepared as described for the preparation of w/o/w emulsion containing trehalose‐6,6′‐dimycolate (TDM) in previous reports (Bekierkunst *et al,* 1969; Yarkoni & Rapp, 1977; Sakai *et al,* 2012). Briefly, 100 μg of chemically synthesized AG was dissolved in 3.2 μl of Freund’s incomplete adjuvant (Chemicon, Temecula, CA), and then, 3.2 μl of 0.1 M PBS was added. The mixture was then homogenized with a homogenizer. Finally, 93.6 μl of saline containing 0.2% Tween 20 were added to the homogenized w/o emulsion and homogenized again. The mice were injected intravenously with a 100 μl of w/o/w emulsion once at a 1‐week interval and were then sacrificed at 14 or 28 days post‐treatment for further analysis. To minimize the effects of subjective bias, blinding of the investigator was performed during group allocation and result analysis.

### Zebrafish embryos infection with *M. marinum*

Wild‐type (AB) zebrafish embryos were infected via Duct of Cuvier injection. Each embryo was infected with 500 cfu of green fluorescent bacteria at 48 h post‐fertilization in the absence or presence of 2 ng aptamer(Takaki *et al,* 2013). Embryos were cultured in egg water containing AG aptamer (50 nM) in optical bottom 48‐well plates with a single embryo per well. The fish water was changed every 2 days, and the survival of embryos was monitored every day.

### Mycobacterial infection of mice

The mouse infection experiments were performed as previously described (Bai *et al,* 2014; Liu *et al,* 2018a; Zheng *et al,* 2018a). For BCG or *Mtb* H37Rv infection, female C57BL/6 mice (6–8 weeks old) or *Galectin‐9* KO mice were intranasally infected with 2 × 10^6^ cfu *Mycobacterium bovis* BCG or *Mtb* H37Rv, with mycobacteria preincubated with 1 μg AG aptamer at 37°C for 40 min or with AG aptamer alone (Chen *et al,* 2007). AG aptamer was further administrated intranasally once at a 1‐week interval for 4 weeks. For mouse survival experiments, 6‐week‐old severe combined immunodeficient (SCID) mice were intranasally infected with 4 × 10^6^ cfu *Mtb* H37Rv or with *Mtb* H37Rv preincubated with 1 μg AG aptamer at 37°C for 40 min. AG aptamer was further administrated intranasally once at a 1‐week interval for 12 weeks. The survival of mice was monitored once at a 1‐week interval. The *Mtb* infection experiments were performed in the Biosafety Level‐3 (BSL‐3) Laboratory. All mouse experiments were performed following the University Health Guide for the Care and Use of Laboratory Animals and were approved by the Biological Research Ethics Committee of Tongji University.

### Histological analysis

Lung tissues from *Mtb*‐infected or AG‐treated mice were fixed in 4% phosphate‐buffered formalin for 24 h and embedded in paraffin wax. The paraffin‐embedded lungs were cut into serial sections with a thickness of 2–3 μm. Hematoxylin and eosin (H&E) staining was applied to detect the infiltration in the lungs. The stained slides were visualized by light microscopy and proceeded with CaseViewer version 2.0 which is a digital microscopy application designed for supporting the histopathological diagnostic workflow and the microscope examination process in bioscience (https://www.3dhistech.com/products‐and‐software/software/digital‐microscopes‐viewers/caseviewer‐old/, 3DHISTECH Ltd.).

### CFU assay

*Mtb* H37Rv*‐*infected mice were killed at 4 weeks after infection. Lungs were collected and homogenized in 1 ml of PBS. Mycobacterial burden was determined by plating tenfold serial dilutions of each tissue homogenate on Middlebrook 7H10 agar plates. Colonies were counted after 3–4 weeks of incubation at 37°C.

### Immunoblot analysis

The cell lysates were extracted using RIPA Lysis Buffer (Beyotime, China) according to the manufacturer’s instructions. The protein sample lysate was separated using 12% sodium dodecyl sulfate (SDS)‐polyacrylamide gel, transferred onto Polyvinylidene Fluoride (PVDF) membrane, and incubated with the appropriate antibodies.

### Immunoprecipitation and GST precipitation

In brief, HEK293T cells were transiently transfected using Lipofectamine2000 (11668; Invitrogen), After 48 h, cells were washed with phosphate‐buffered saline (PBS) and then lysed in cell‐lysis buffer for Western blotting and immunoprecipitation (Beyotime). Cellular debris was cleared by centrifugation at ~12,000 *g* for 15 min. For immunoprecipitation, cell lysates were incubated with anti‐Flag M2 Affinity Gel (A2220, Sigma‐Aldrich) and anti‐GFP agarose beads antibody (AE074, ABclonal) at 4°C overnight. For endogenous immunoprecipitation, primary peritoneal macrophages or THP‐1 cells were stimulated with AG for the indicated time periods. The cells were lysed, and the lysates were incubated with an anti‐TAK1 antibody and protein A/G (sepharose) at 4°C overnight. The sepharose samples were centrifuged, washed five times with cell‐lysis buffer, and denatured at 95°C with SDS loading buffer for 10 min. After separation by SDS–PAGE, equivalent amounts of protein were electroblotted onto nitrocellulose membranes or polyvinylidene difluoride membranes. The membranes were blocked, incubated with primary antibodies at the indicated dilutions, and washed three times before incubation with secondary antibody. After a final wash, the analysis was conducted using an enhanced chemiluminescence reagent (Thermo Fisher Scientific). Immunoprecipitation and GST precipitation were also performed as previously described (Wang *et al,* 2020).

### Confocal microscopy

Confocal microscopy was performed as described (Liu *et al,* 2018b).

### Real‐time quantitative reverse‐transcription PCR

Total RNA was extracted with 1 ml of TRIzol reagent according to the manufacturer’s instructions (Invitrogen). Next, 1 μg of total RNA was reverse transcribed using the ReverTra Ace^®^ qPCR RT Kit (Toyobo, FSQ‐101) according to the manufacturer’s instructions. A SYBR RT‐PCR kit (Toyobo, QPK‐212) was used for quantitative real‐time RT–PCR analysis. The relative mRNA expression of different genes was calculated by comparison with the control gene *Gapdh* (encoding GAPDH) using the 2^−△△Ct^ method. Gene expression was normalized to that of *gapdh*. Real‐time quantitative reverse‐transcription PCR (qRT–PCR) data were representative of at least 3 independent experiments, with 2 technical replicates per experiment. Primer sequences are listed in Appendix Table S1.

### RNA‐seq analysis

Total RNA was isolated and used for RNA‐seq analysis. cDNA library construction and sequencing were performed by Beijing Genomics Institute using BGISEQ‐500 platform. High‐quality reads were aligned to the *Mus musculus* reference genome (UCSC_mm10) using Bowtie2. The expression levels for each of the genes were normalized to fragments per kilobase of exon model per million mapped reads (FPKM) using RNA‐seq by Expectation Maximization (RSEM).

### Statistical analysis

Statistical analysis was performed by two‐tailed Student’s *t‐*test or one‐way ANOVA followed by Dunnett's *post hoc* test or two‐way ANOVA followed by Tukey's *post hoc* test or Mann–Whitney U‐test using GraphPad Prism 7 (GraphPad Software). All data are expressed as mean + SD of the averages of technical replicates from the indicated number of independent experiments. Differences with values of *P* < 0.05 were considered statistically significant. The number "n" in the figure legends means how many independent experiments (biological replicates) are performed. The bars and error bars and the test are used to calculate *P*‐values in the respective figure legends.

## Author contributions

Project conception, experiment design, and manuscript writing: HL, SHEK, X‐SY, and BG; Most of the experiments and data analysis: XW, RZ, and YW; Aptamer screening assay: LQ and DL; Gene knockdown *M. marinum:* LZ and JZ; Mouse survival experiment: LC and GZ; Zebrafish larvae survival experiment: BY, HY, and YW; Experiments and technical help: FT, FL, FW, LW, MM, ZL, JianC, XH, JW, RJ, PW, QS, WS, LL, AD, GP, YC, and PM‐A; Galectin‐9 knockout mice: JiayC and SG; Helpful comments: PZ, FR, and CC.

## Conflict of interest

The authors declare that they have no conflict of interest.

## Supporting information



Appendix

Expanded View Figures PDF

Source Data for Expanded View

Source Data for Figure 2

Source Data for Figure 3

Source Data for Figure 6

Source Data for Figure 7

## Data Availability

The RNA‐Seq data have been deposited to the Gene Expression Ominibus (GEO) database (accession number: GSE166850, https://www.ncbi.nlm.nih.gov/geo/query/acc.cgi?acc=GSE166850). All other data are available in the manuscript text and supporting information.

## References

[embr202051678-bib-0001] Aimaiti R , Qin L , Cao T , Yang H , Wang J , Lu J , Huang X , Hu Z (2015) Identification and application of ssDNA aptamers against H(3)(7)Rv in the detection of *Mycobacterium tuberculosis* . Appl Microbiol Biotechnol 99: 9073–9083 26194558 10.1007/s00253-015-6815-7

[embr202051678-bib-0002] Ajibade AA , Wang HY , Wang RF (2013) Cell type‐specific function of TAK1 in innate immune signaling. Trends Immunol 34: 307–316 23664135 10.1016/j.it.2013.03.007

[embr202051678-bib-0003] Alderwick LJ , Harrison J , Lloyd GS , Birch HL (2015) The mycobacterial cell wall‐peptidoglycan and arabinogalactan. Cold Spring Harb Perspect Med 5: a021113 25818664 10.1101/cshperspect.a021113 PMC4526729

[embr202051678-bib-0004] Bai W , Liu H , Ji Q , Zhou Y , Liang Le , Zheng R , Chen J , Liu Z , Yang H , Zhang P *et al* (2014) TLR3 regulates mycobacterial RNA‐induced IL‐10 production through the PI3K/AKT signaling pathway. Cell Signal 26: 942–950 24462705 10.1016/j.cellsig.2014.01.015

[embr202051678-bib-0005] Barboni E , Coade S , Fiori A (2005) The binding of mycolic acids to galectin‐3: a novel interaction between a host soluble lectin and trafficking mycobacterial lipids? FEBS Lett 579: 6749–6755 16310777 10.1016/j.febslet.2005.11.005

[embr202051678-bib-0006] Bastos PAD , Wheeler R , Boneca IG (2021) Uptake, recognition and responses to peptidoglycan in the mammalian host. FEMS Microbiol Rev 45: fuaa044 32897324 10.1093/femsre/fuaa044 PMC7794044

[embr202051678-bib-0007] Bekierkunst A (1968) Acute granulomatous response produced in mice by trehalose‐6,6‐dimycolate. J Bacteriol 96: 958–961 4971895 10.1128/jb.96.4.958-961.1968 PMC252404

[embr202051678-bib-0008] Bekierkunst A , Levij IS , Yarkoni E , Vilkas E , Adam A , Lederer E (1969) Granuloma formation induced in mice by chemically defined mycobacterial fractions. J Bacteriol 100: 95–102 4981067 10.1128/jb.100.1.95-102.1969 PMC315363

[embr202051678-bib-0009] Bock LC , Griffin LC , Latham JA , Vermaas EH , Toole JJ (1992) Selection of single‐stranded DNA molecules that bind and inhibit human thrombin. Nature 355: 564–566 1741036 10.1038/355564a0

[embr202051678-bib-0010] Brilha S , Sathyamoorthy T , Stuttaford LH , Walker NF , Wilkinson RJ , Singh S , Moores RC , Elkington PT , Friedland JS (2017) Early secretory antigenic target‐6 drives matrix metalloproteinase‐10 gene expression and secretion in tuberculosis. Am J Respir Cell Mol Biol 56: 223–232 27654284 10.1165/rcmb.2016-0162OC PMC5359650

[embr202051678-bib-0011] Cao A , Alluqmani N , Buhari FHM , Wasim L , Smith LK , Quaile AT , Shannon M , Hakim Z , Furmli H , Owen DM *et al* (2018) Galectin‐9 binds IgM‐BCR to regulate B cell signaling. Nat Commun 9: 3288 30120235 10.1038/s41467-018-05771-8 PMC6098130

[embr202051678-bib-0012] Chai Q , Wang L , Liu CH , Ge B (2020) New insights into the evasion of host innate immunity by *Mycobacterium tuberculosis* . Cell Mol Immunol 17: 901–913 32728204 10.1038/s41423-020-0502-z PMC7608469

[embr202051678-bib-0013] Chan YC , Lin HY , Tu Z , Kuo YH , Hsu SD , Lin CH (2018) Dissecting the structure‐activity relationship of galectin‐ligand interactions. Int J Mol Sci 19: 392 10.3390/ijms19020392 PMC5855614 29382172

[embr202051678-bib-0014] Chavez‐Galan L , Ramon‐Luing L , Carranza C , Garcia I , Sada‐Ovalle I (2017) lipoarabinomannan decreases galectin‐9 expression and tumor necrosis factor pathway in macrophages favoring *Mycobacterium tuberculosis* intracellular growth. Front Immunol 8: 1659 29230224 10.3389/fimmu.2017.01659 PMC5711832

[embr202051678-bib-0015] Chen F , Zhou J , Luo F , Mohammed AB , Zhang XL (2007) Aptamer from whole‐bacterium SELEX as new therapeutic reagent against virulent *Mycobacterium tuberculosis* . Biochem Biophys Res Commun 357: 743–748 17442275 10.1016/j.bbrc.2007.04.007

[embr202051678-bib-0016] Cui Z , Li Y , Cheng S , Yang H , Lu J , Hu Z , Ge B (2014) Mutations in the embC‐embA intergenic region contribute to *Mycobacterium tuberculosis* resistance to ethambutol. Antimicrob Agents Chemother 58: 6837–6843 25182646 10.1128/AAC.03285-14 PMC4249443

[embr202051678-bib-0017] Dey B , Dey RJ , Cheung LS , Pokkali S , Guo H , Lee JH , Bishai WR (2015) A bacterial cyclic dinucleotide activates the cytosolic surveillance pathway and mediates innate resistance to tuberculosis. Nat Med 21: 401–406 25730264 10.1038/nm.3813 PMC4390473

[embr202051678-bib-0018] Dey RJ , Dey B , Zheng Y , Cheung LS , Zhou J , Sayre D , Kumar P , Guo H , Lamichhane G , Sintim HO *et al* (2017) Inhibition of innate immune cytosolic surveillance by an *M. tuberculosis* phosphodiesterase. Nat Chem Biol 13: 210–217 28106876 10.1038/nchembio.2254

[embr202051678-bib-0019] Dorhoi A , Kaufmann SH (2016) Pathology and immune reactivity: understanding multidimensionality in pulmonary tuberculosis. Semin Immunopathol 38: 153–166 26438324 10.1007/s00281-015-0531-3

[embr202051678-bib-0020] Dulberger CL , Rubin EJ , Boutte CC (2020) The mycobacterial cell envelope ‐ a moving target. Nat Rev Microbiol 18: 47–59 31728063 10.1038/s41579-019-0273-7

[embr202051678-bib-0021] Ernst JD (2012) The immunological life cycle of tuberculosis. Nat Rev Immunol 12: 581–591 22790178 10.1038/nri3259

[embr202051678-bib-0022] Escuyer VE , Lety MA , Torrelles JB , Khoo KH , Tang JB , Rithner CD , Frehel C , McNeil MR , Brennan PJ , Chatterjee D (2001) The role of the embA and embB gene products in the biosynthesis of the terminal hexaarabinofuranosyl motif of *Mycobacterium smegmatis* arabinogalactan. J Biol Chem 276: 48854–48862 11677227 10.1074/jbc.M102272200

[embr202051678-bib-0023] Esin S , Counoupas C , Aulicino A , Brancatisano FL , Maisetta G , Bottai D , Di Luca M , Florio W , Campa M , Batoni G (2013) Interaction of *Mycobacterium tuberculosis* cell wall components with the human natural killer cell receptors NKp44 and Toll‐like receptor 2. Scand J Immunol 77: 460–469 23578092 10.1111/sji.12052

[embr202051678-bib-0024] Giovannone N , Liang J , Antonopoulos A , Geddes Sweeney J , King Sl , Pochebit Sm , Bhattacharyya N , Lee Gs , Dell A , Widlund Hr *et al* (2018) Galectin‐9 suppresses B cell receptor signaling and is regulated by I‐branching of N‐glycans. Nat Commun 9: 3287 30120234 10.1038/s41467-018-05770-9 PMC6098069

[embr202051678-bib-0025] Golichenari B , Nosrati R , Farokhi‐Fard A , Abnous K , Vaziri F , Behravan J (2018) Nano‐biosensing approaches on tuberculosis: defy of aptamers. Biosens Bioelectron 117: 319–331 29933223 10.1016/j.bios.2018.06.025

[embr202051678-bib-0026] Goude R , Amin AG , Chatterjee D , Parish T (2009) The arabinosyltransferase EmbC is inhibited by ethambutol in *Mycobacterium tuberculosis* . Antimicrob Agents Chemother 53: 4138–4146 19596878 10.1128/AAC.00162-09 PMC2764220

[embr202051678-bib-0027] Grzegorzewicz AE , de Sousa‐d'Auria C , McNeil MR , Huc‐Claustre E , Jones V , Petit C , Angala SK , Zemanová J , Wang Q , Belardinelli JM *et al* (2016) Assembling of the *Mycobacterium tuberculosis* cell wall core. J Biol Chem 291: 18867–18879 27417139 10.1074/jbc.M116.739227 PMC5009262

[embr202051678-bib-0028] Heusschen R , Freitag N , Tirado‐Gonzalez I , Barrientos G , Moschansky P , Munoz‐Fernandez R , Leno‐Duran E , Klapp BF , Thijssen VL , Blois SM (2013) Profiling Lgals9 splice variant expression at the fetal‐maternal interface: implications in normal and pathological human pregnancy. Biol Reprod 88: 22 23242525 10.1095/biolreprod.112.105460

[embr202051678-bib-0029] Hmama Z , Pena‐Diaz S , Joseph S , Av‐Gay Y (2015) Immunoevasion and immunosuppression of the macrophage by *Mycobacterium tuberculosis* . Immunol Rev 264: 220–232 25703562 10.1111/imr.12268

[embr202051678-bib-0030] Houben D , Demangel C , van Ingen J , Perez J , Baldeón L , Abdallah AM , Caleechurn L , Bottai D , van Zon M , de Punder K *et al* (2012) ESX‐1‐mediated translocation to the cytosol controls virulence of mycobacteria. Cell Microbiol 14: 1287–1298 22524898 10.1111/j.1462-5822.2012.01799.x

[embr202051678-bib-0031] Hrabec E , Strek M , Zieba M , Kwiatkowska S , Hrabec Z (2002) Circulation level of matrix metalloproteinase‐9 is correlated with disease severity in tuberculosis patients. Int J Tuberc Lung Dis 6: 713–719 12150484

[embr202051678-bib-0032] Ishikawa E , Ishikawa T , Morita YS , Toyonaga K , Yamada H , Takeuchi O , Kinoshita T , Akira S , Yoshikai Y , Yamasaki S (2009) Direct recognition of the mycobacterial glycolipid, trehalose dimycolate, by C‐type lectin Mincle. J Exp Med 206: 2879–2888 20008526 10.1084/jem.20091750 PMC2806462

[embr202051678-bib-0033] Ito T , Schaller M , Hogaboam CM , Standiford TJ , Sandor M , Lukacs NW , Chensue SW , Kunkel SL (2009) TLR9 regulates the mycobacteria‐elicited pulmonary granulomatous immune response in mice through DC‐derived Notch ligand delta‐like 4. J Clin Invest 119: 33–46 19075396 10.1172/JCI35647 PMC2613456

[embr202051678-bib-0034] Jankute M , Cox JA , Harrison J , Besra GS (2015) Assembly of the mycobacterial cell wall. Annu Rev Microbiol 69: 405–423 26488279 10.1146/annurev-micro-091014-104121

[embr202051678-bib-0035] Jayaraman P , Sada‐Ovalle I , Beladi S , Anderson AC , Dardalhon V , Hotta C , Kuchroo VK , Behar SM (2010) Tim3 binding to galectin‐9 stimulates antimicrobial immunity. J Exp Med 207: 2343–2354 20937702 10.1084/jem.20100687 PMC2964580

[embr202051678-bib-0036] Jia J , Abudu YP , Claude‐Taupin A , Gu Y , Kumar S , Choi SW , Peters R , Mudd MH , Allers L , Salemi M *et al* (2018) Galectins control mTOR in response to endomembrane damage. Mol Cell 70: 120–135.e8 29625033 10.1016/j.molcel.2018.03.009 PMC5911935

[embr202051678-bib-0037] Jia J , Abudu YP , Claude‐Taupin A , Gu Y , Kumar S , Choi SW , Peters R , Mudd MH , Allers L , Salemi M *et al* (2019) Galectins control MTOR and AMPK in response to lysosomal damage to induce autophagy. Autophagy 15: 169–171 30081722 10.1080/15548627.2018.1505155 PMC6287685

[embr202051678-bib-0038] Jia J , Bissa B , Brecht L , Allers L , Choi SW , Gu Y , Zbinden M , Burge MR , Timmins G , Hallows K *et al* (2020) AMPK, a regulator of metabolism and autophagy, is activated by lysosomal damage via a novel galectin‐directed ubiquitin signal transduction system. Mol Cell 77: 951–969.e9 31995728 10.1016/j.molcel.2019.12.028 PMC7785494

[embr202051678-bib-0039] Juarez E , Carranza C , Hernandez‐Sanchez F , Leon‐Contreras JC , Hernandez‐Pando R , Escobedo D , Torres M , Sada E (2012) NOD2 enhances the innate response of alveolar macrophages to *Mycobacterium tuberculosis* in humans. Eur J Immunol 42: 880–889 22531915 10.1002/eji.201142105

[embr202051678-bib-0040] Kanwar YS , Carone FA , Kumar A , Wada J , Ota K , Wallner EI (1997) Role of extracellular matrix, growth factors and proto‐oncogenes in metanephric development. Kidney Int 52: 589–606 9291177 10.1038/ki.1997.372

[embr202051678-bib-0041] Kathamuthu GR , Kumar NP , Moideen K , Nair D , Banurekha VV , Sridhar R , Baskaran D , Babu S (2020) Matrix metalloproteinases and tissue inhibitors of metalloproteinases are potential biomarkers of pulmonary and extra‐pulmonary tuberculosis. Front Immunol 11: 419 32218787 10.3389/fimmu.2020.00419 PMC7078103

[embr202051678-bib-0042] Kaufmann SHE , Dorhoi A , Hotchkiss RS , Bartenschlager R (2018) Host‐directed therapies for bacterial and viral infections. Nat Rev Drug Discov 17: 35–56 28935918 10.1038/nrd.2017.162 PMC7097079

[embr202051678-bib-0043] Kaufmann SH , Lange C , Rao M , Balaji KN , Lotze M , Schito M , Zumla AI , Maeurer M (2014) Progress in tuberculosis vaccine development and host‐directed therapies–a state of the art review. Lancet Respir Med 2: 301–320 24717627 10.1016/S2213-2600(14)70033-5

[embr202051678-bib-0044] Killick KE , Ni Cheallaigh C , O'Farrelly C , Hokamp K , MacHugh DE , Harris J (2013) Receptor‐mediated recognition of mycobacterial pathogens. Cell Microbiol 15: 1484–1495 23795683 10.1111/cmi.12161

[embr202051678-bib-0045] Kong L , Sun L , Zhang H , Liu Q , Liu Ye , Qin L , Shi G , Hu J‐H , Xu A , Sun Y‐P *et al* (2009) An essential role for RIG‐I in toll‐like receptor‐stimulated phagocytosis. Cell Host Microbe 6: 150–161 19683681 10.1016/j.chom.2009.06.008

[embr202051678-bib-0046] Korf J , Stoltz A , Verschoor J , De Baetselier P , Grooten J (2005) The *Mycobacterium tuberculosis* cell wall component mycolic acid elicits pathogen‐associated host innate immune responses. Eur J Immunol 35: 890–900 15724242 10.1002/eji.200425332

[embr202051678-bib-0047] Kubler A , Luna B , Larsson C , Ammerman NC , Andrade BB , Orandle M , Bock KW , Xu Z , Bagci U , Mollura DJ *et al* (2015) *Mycobacterium tuberculosis* dysregulates MMP/TIMP balance to drive rapid cavitation and unrestrained bacterial proliferation. J Pathol 235: 431–444 25186281 10.1002/path.4432 PMC4293239

[embr202051678-bib-0048] Kumar NP , Moideen K , Nancy A , Viswanathan V , Thiruvengadam K , Sivakumar S , Hissar S , Nair D , Banurekha VV , Kornfeld H *et al* (2020) Association of plasma matrix metalloproteinase and tissue inhibitors of matrix metalloproteinase levels with adverse treatment outcomes among patients with pulmonary tuberculosis. JAMA Netw Open 3: e2027754 33258908 10.1001/jamanetworkopen.2020.27754 PMC7709089

[embr202051678-bib-0049] Laaf D , Bojarova P , Elling L , Kren V (2018) Galectin‐carbohydrate interactions in biomedicine and biotechnology. Trends Biotechnol 37: 402–415 30413271 10.1016/j.tibtech.2018.10.001

[embr202051678-bib-0050] Ladel CH , Blum C , Dreher A , Reifenberg K , Kopf M , Kaufmann SH (1997) Lethal tuberculosis in interleukin‐6‐deficient mutant mice. Infect Immun 65: 4843–4849 9353074 10.1128/iai.65.11.4843-4849.1997 PMC175695

[embr202051678-bib-0051] Laderach DJ , Compagno D , Toscano MA , Croci DO , Dergan‐Dylon S , Salatino M , Rabinovich GA (2010) Dissecting the signal transduction pathways triggered by galectin‐glycan interactions in physiological and pathological settings. IUBMB Life 62: 1–13 20014236 10.1002/iub.281

[embr202051678-bib-0052] Lee WB , Kang JS , Yan JJ , Lee MS , Jeon BY , Cho SN , Kim YJ (2012) Neutrophils promote mycobacterial trehalose dimycolate‐induced lung inflammation via the mincle pathway. PLoS Pathog 8: e1002614 22496642 10.1371/journal.ppat.1002614 PMC3320589

[embr202051678-bib-0053] Liu CH , Liu H , Ge B (2017) Innate immunity in tuberculosis: host defense vs pathogen evasion. Cell Mol Immunol 14: 963–975 28890547 10.1038/cmi.2017.88 PMC5719146

[embr202051678-bib-0054] Liu F , Chen J , Wang P , Li H , Zhou Y , Liu H , Liu Z , Zheng R , Wang L , Yang H *et al* (2018) MicroRNA‐27a controls the intracellular survival of *Mycobacterium tuberculosis* by regulating calcium‐associated autophagy. Nat Commun 9: 4295 30327467 10.1038/s41467-018-06836-4 PMC6191460

[embr202051678-bib-0055] Liu H , Zhang H , Wu X , Ma D , Wu J , Wang L , Jiang Y , Fei Y , Zhu C , Tan R *et al* (2018) Nuclear cGAS suppresses DNA repair and promotes tumorigenesis. Nature 563: 131–136 30356214 10.1038/s41586-018-0629-6

[embr202051678-bib-0056] Liu J , Barry CE , Besra GS , Nikaido H (1996) Mycolic acid structure determines the fluidity of the mycobacterial cell wall. J Biol Chem 271: 29545–29551 8939881 10.1074/jbc.271.47.29545

[embr202051678-bib-0057] Liu W , Li K , Bai D , Yin J , Tang Y , Chi F , Zhang L , Wang Yu , Pan J , Liang S *et al* (2017) Dosage effects of ZP2 and ZP3 heterozygous mutations cause human infertility. Hum Genet 136: 975–985 28646452 10.1007/s00439-017-1822-7

[embr202051678-bib-0058] Lou J , Wang Y , Zhang Z , Qiu W (2017) Activation of MMPs in macrophages by *Mycobacterium tuberculosis* via the miR‐223‐BMAL1 signaling pathway. J Cell Biochem 118: 4804–4812 28543681 10.1002/jcb.26150

[embr202051678-bib-0059] Lu Y , Zheng Y , Coyaud É , Zhang C , Selvabaskaran A , Yu Y , Xu Z , Weng X , Chen JS , Meng Y *et al* (2019) Palmitoylation of NOD1 and NOD2 is required for bacterial sensing. Science 366: 460–467 31649195 10.1126/science.aau6391

[embr202051678-bib-0060] Mayer‐Barber KD , Andrade BB , Oland SD , Amaral EP , Barber DL , Gonzales J , Derrick SC , Shi R , Kumar NP , Wei W *et al* (2014) Host‐directed therapy of tuberculosis based on interleukin‐1 and type I interferon crosstalk. Nature 511: 99–103 24990750 10.1038/nature13489 PMC4809146

[embr202051678-bib-0061] Meniche X , Otten R , Siegrist MS , Baer CE , Murphy KC , Bertozzi CR , Sassetti CM (2014) Subpolar addition of new cell wall is directed by DivIVA in mycobacteria. Proc Natl Acad Sci USA 111: E3243–3251 25049412 10.1073/pnas.1402158111 PMC4128124

[embr202051678-bib-0062] Mishra BB , Moura‐Alves P , Sonawane A , Hacohen N , Griffiths G , Moita LF , Anes E (2010) *Mycobacterium tuberculosis* protein ESAT‐6 is a potent activator of the NLRP3/ASC inflammasome. Cell Microbiol 12: 1046–1063 20148899 10.1111/j.1462-5822.2010.01450.x

[embr202051678-bib-0063] Mortaz E , Adcock IM , Tabarsi P , Masjedi MR , Mansouri D , Velayati AA , Casanova JL , Barnes PJ (2015) Interaction of pattern recognition receptors with *Mycobacterium tuberculosis* . J Clin Immunol 35: 1–10 10.1007/s10875-014-0103-7 PMC4306732 25312698

[embr202051678-bib-0064] Moura‐Alves P , Faé K , Houthuys E , Dorhoi A , Kreuchwig A , Furkert J , Barison N , Diehl A , Munder A , Constant P *et al* (2014) AhR sensing of bacterial pigments regulates antibacterial defence. Nature 512: 387–392 25119038 10.1038/nature13684

[embr202051678-bib-0065] Nagae M , Nishi N , Nakamura‐Tsuruta S , Hirabayashi J , Wakatsuki S , Kato R (2008) Structural analysis of the human galectin‐9 N‐terminal carbohydrate recognition domain reveals unexpected properties that differ from the mouse orthologue. J Mol Biol 375: 119–135 18005988 10.1016/j.jmb.2007.09.060

[embr202051678-bib-0066] Ninomiya‐Tsuji J , Kajino T , Ono K , Ohtomo T , Matsumoto M , Shiina M , Mihara M , Tsuchiya M , Matsumoto K (2003) A resorcylic acid lactone, 5Z‐7‐oxozeaenol, prevents inflammation by inhibiting the catalytic activity of TAK1 MAPK kinase kinase. J Biol Chem 278: 18485–18490 12624112 10.1074/jbc.M207453200

[embr202051678-bib-0067] O'Garra A , Redford PS , McNab FW , Bloom CI , Wilkinson RJ , Berry MP (2013) The immune response in tuberculosis. Annu Rev Immunol 31: 475–527 23516984 10.1146/annurev-immunol-032712-095939

[embr202051678-bib-0068] Olaru A , Bala C , Jaffrezic‐Renault N , Aboul‐Enein HY (2015) Surface plasmon resonance (SPR) biosensors in pharmaceutical analysis. Crit Rev Anal Chem 45: 97–105 25558771 10.1080/10408347.2014.881250

[embr202051678-bib-0069] Ong CW , Elkington PT , Friedland JS (2014) Tuberculosis, pulmonary cavitation, and matrix metalloproteinases. Am J Respir Crit Care Med 190: 9–18 24713029 10.1164/rccm.201311-2106PP PMC4226026

[embr202051678-bib-0070] Ordonez AA , Tasneen R , Pokkali S , Xu Z , Converse PJ , Klunk MH , Mollura DJ , Nuermberger EL , Jain SK (2016) Mouse model of pulmonary cavitary tuberculosis and expression of matrix metalloproteinase‐9. Dis Model Mech 9: 779–788 27482816 10.1242/dmm.025643 PMC4958312

[embr202051678-bib-0071] Orme IM , Robinson RT , Cooper AM (2015) The balance between protective and pathogenic immune responses in the TB‐infected lung. Nat Immunol 16: 57–63 25521685 10.1038/ni.3048

[embr202051678-bib-0072] Parasa VR , Muvva JR , Rose JF , Braian C , Brighenti S , Lerm M (2017) Inhibition of tissue matrix metalloproteinases interferes with *Mycobacterium tuberculosis*‐induced granuloma formation and reduces bacterial load in a human lung tissue model. Front Microbiol 8: 2370 29259583 10.3389/fmicb.2017.02370 PMC5723394

[embr202051678-bib-0073] Pathak SK , Basu S , Basu KK , Banerjee A , Pathak S , Bhattacharyya A , Kaisho T , Kundu M , Basu J (2007) Direct extracellular interaction between the early secreted antigen ESAT‐6 of *Mycobacterium tuberculosis* and TLR2 inhibits TLR signaling in macrophages. Nat Immunol 8: 610–618 17486091 10.1038/ni1468

[embr202051678-bib-0074] Perrin AJ , Jiang X , Birmingham CL , So NS , Brumell JH (2004) Recognition of bacteria in the cytosol of Mammalian cells by the ubiquitin system. Curr Biol 14: 806–811 15120074 10.1016/j.cub.2004.04.033

[embr202051678-bib-0075] Pieters J (2008) *Mycobacterium tuberculosis* and the macrophage: maintaining a balance. Cell Host Microbe 3: 399–407 18541216 10.1016/j.chom.2008.05.006

[embr202051678-bib-0076] Qin LH , Liu ZH , Yang H , Cai JL , Bai WJ , Wang J , Liu JM , Hu ZY (2014) Dynamic evolution and immunoreactivity of aptamers binding to polyclonal antibodies against MPT64 antigen of *Mycobacterium tuberculosis* . Eur J Clin Microbiol Infect Dis 33: 1199–1209 24500599 10.1007/s10096-014-2056-4

[embr202051678-bib-0077] Qin L , Zheng R , Ma Z , Feng Y , Liu Z , Yang H , Wang J , Jin R , Lu J , Ding Y *et al* (2009) The selection and application of ssDNA aptamers against MPT64 protein in *Mycobacterium tuberculosis* . Clin Chem Lab Med 47: 405–411 19284297 10.1515/CCLM.2009.097

[embr202051678-bib-0078] Rabinovich GA , Toscano MA (2009) Turning 'sweet' on immunity: galectin‐glycan interactions in immune tolerance and inflammation. Nat Rev Immunol 9: 338–352 19365409 10.1038/nri2536

[embr202051678-bib-0079] Rand L , Green JA , Saraiva L , Friedland JS , Elkington PT (2009) Matrix metalloproteinase‐1 is regulated in tuberculosis by a p38 MAPK‐dependent, p‐aminosalicylic acid‐sensitive signaling cascade. J Immunol 182: 5865–5872 19380835 10.4049/jimmunol.0801935

[embr202051678-bib-0080] Rivera‐Marrero CA , Schuyler W , Roser S , Ritzenthaler JD , Newburn SA , Roman J (2002) *M. tuberculosis* induction of matrix metalloproteinase‐9: the role of mannose and receptor‐mediated mechanisms. Am J Physiol Lung Cell Mol Physiol 282: L546–555 11839551 10.1152/ajplung.00175.2001

[embr202051678-bib-0081] Robinson RT , Orme IM , Cooper AM (2015) The onset of adaptive immunity in the mouse model of tuberculosis and the factors that compromise its expression. Immunol Rev 264: 46–59 25703551 10.1111/imr.12259

[embr202051678-bib-0082] Rock JM , Hopkins FF , Chavez A , Diallo M , Chase MR , Gerrick ER , Pritchard JR , Church GM , Rubin EJ , Sassetti CM *et al* (2017) Programmable transcriptional repression in mycobacteria using an orthogonal CRISPR interference platform. Nat Microbiol 2: 16274 28165460 10.1038/nmicrobiol.2016.274 PMC5302332

[embr202051678-bib-0083] Sabir N , Hussain T , Mangi MH , Zhao D , Zhou X (2019) Matrix metalloproteinases: expression, regulation and role in the immunopathology of tuberculosis. Cell Prolif 52: e12649 31199047 10.1111/cpr.12649 PMC6668971

[embr202051678-bib-0084] Sada‐Ovalle I , Chavez‐Galan L , Torre‐Bouscoulet L , Nava‐Gamino L , Barrera L , Jayaraman P , Torres‐Rojas M , Salazar‐Lezama MA , Behar SM (2012) The Tim3‐galectin 9 pathway induces antibacterial activity in human macrophages infected with *Mycobacterium tuberculosis* . J Immunol 189: 5896–5902 23180819 10.4049/jimmunol.1200990 PMC3516679

[embr202051678-bib-0085] Saiga H , Kitada S , Shimada Y , Kamiyama N , Okuyama M , Makino M , Yamamoto M , Takeda K (2012) Critical role of AIM2 in *Mycobacterium tuberculosis* infection. Int Immunol 24: 637–644 22695634 10.1093/intimm/dxs062

[embr202051678-bib-0086] Sakai Y , Uchida K , Nakayama H (2012) A20 and ABIN‐3 possibly promote regression of trehalose 6,6'‐dimycolate (TDM)‐induced granuloma by interacting with an NF‐kappa B signaling protein, TAK‐1. Inflamm Res 61: 245–253 22173278 10.1007/s00011-011-0406-6

[embr202051678-bib-0087] Simeone R , Sayes F , Song O , Groschel MI , Brodin P , Brosch R , Majlessi L (2015) Cytosolic access of *Mycobacterium tuberculosis*: critical impact of phagosomal acidification control and demonstration of occurrence *in vivo* . PLoS Pathog 11: e1004650 25658322 10.1371/journal.ppat.1004650 PMC4450080

[embr202051678-bib-0088] Singhirunnusorn P , Suzuki S , Kawasaki N , Saiki I , Sakurai H (2005) Critical roles of threonine 187 phosphorylation in cellular stress‐induced rapid and transient activation of transforming growth factor‐beta‐activated kinase 1 (TAK1) in a signaling complex containing TAK1‐binding protein TAB1 and TAB2. J Biol Chem 280: 7359–7368 15590691 10.1074/jbc.M407537200

[embr202051678-bib-0089] Skipper JB , McNally LR , Rosenthal EL , Wang W , Buchsbaum DJ (2009) *In vivo* efficacy of marimastat and chemoradiation in head and neck cancer xenografts. ORL J Otorhinolaryngol Relat Spec 71: 1–5 18931526 10.1159/000163217 PMC2700630

[embr202051678-bib-0090] Sousa J , Ca B , Maceiras AR , Simoes‐Costa L , Fonseca KL , Fernandes AI , Ramos A , Carvalho T , Barros L , Magalhaes C *et al* (2020) *Mycobacterium tuberculosis* associated with severe tuberculosis evades cytosolic surveillance systems and modulates IL‐1beta production. Nat Commun 11: 1949 32327653 10.1038/s41467-020-15832-6 PMC7181847

[embr202051678-bib-0091] Stamm CE , Collins AC , Shiloh MU (2015) Sensing of *Mycobacterium tuberculosis* and consequences to both host and bacillus. Immunol Rev 264: 204–219 25703561 10.1111/imr.12263 PMC4339209

[embr202051678-bib-0092] Sun X , Pan Q , Yuan C , Wang Q , Tang XL , Ding K , Zhou X , Zhang XL (2016) A Single ssDNA aptamer binding to mannose‐capped lipoarabinomannan of bacillus calmette‐guerin enhances immunoprotective effect against tuberculosis. J Am Chem Soc 138: 11680–11689 27529508 10.1021/jacs.6b05357

[embr202051678-bib-0093] Tahiri N , Fodran P , Jayaraman D , Buter J , Witte MD , Ocampo TA , Moody DB , Van Rhijn I , Minnaard AJ (2020) Total synthesis of a mycolic acid from *Mycobacterium tuberculosis* . Angew Chem Int Ed Engl 59: 7555–7560 32067294 10.1002/anie.202000523 PMC7216993

[embr202051678-bib-0094] Takaki K , Davis JM , Winglee K , Ramakrishnan L (2013) Evaluation of the pathogenesis and treatment of *Mycobacterium marinum* infection in zebrafish. Nat Protoc 8: 1114–1124 23680983 10.1038/nprot.2013.068 PMC3919459

[embr202051678-bib-0095] Tang XL , Wu SM , Xie Y , Song N , Guan Q , Yuan C , Zhou X , Zhang XL (2016) Generation and application of ssDNA aptamers against glycolipid antigen ManLAM of *Mycobacterium tuberculosis* for TB diagnosis. J Infect 72: 573–586 26850356 10.1016/j.jinf.2016.01.014

[embr202051678-bib-0096] Toyonaga K , Torigoe S , Motomura Y , Kamichi T , Hayashi JM , Morita YS , Noguchi N , Chuma Y , Kiyohara H , Matsuo K *et al* (2016) C‐Type lectin receptor DCAR recognizes mycobacterial phosphatidyl‐inositol mannosides to promote a Th1 response during infection. Immunity 45: 1245–1257 27887882 10.1016/j.immuni.2016.10.012

[embr202051678-bib-0097] Tuerk C , Gold L (1990) Systematic evolution of ligands by exponential enrichment: RNA ligands to bacteriophage T4 DNA polymerase. Science 249: 505–510 2200121 10.1126/science.2200121

[embr202051678-bib-0098] Tureci O , Schmitt H , Fadle N , Pfreundschuh M , Sahin U (1997) Molecular definition of a novel human galectin which is immunogenic in patients with Hodgkin's disease. J Biol Chem 272: 6416–6422 9045665 10.1074/jbc.272.10.6416

[embr202051678-bib-0099] Verschoor JA , Baird MS , Grooten J (2012) Towards understanding the functional diversity of cell wall mycolic acids of *Mycobacterium tuberculosis* . Prog Lipid Res 51: 325–339 22659327 10.1016/j.plipres.2012.05.002

[embr202051678-bib-0100] Volkman HE , Pozos TC , Zheng J , Davis JM , Rawls JF , Ramakrishnan L (2010) Tuberculous granuloma induction via interaction of a bacterial secreted protein with host epithelium. Science 327: 466–469 20007864 10.1126/science.1179663 PMC3125975

[embr202051678-bib-0101] Walker NF , Clark SO , Oni T , Andreu N , Tezera L , Singh S , Saraiva L , Pedersen B , Kelly DL , Tree JA *et al* (2012) Doxycycline and HIV infection suppress tuberculosis‐induced matrix metalloproteinases. Am J Respir Crit Care Med 185: 989–997 22345579 10.1164/rccm.201110-1769OC PMC3359940

[embr202051678-bib-0102] Wang H , Yang H , Shivalila CS , Dawlaty MM , Cheng AW , Zhang F , Jaenisch R (2013) One‐step generation of mice carrying mutations in multiple genes by CRISPR/Cas‐mediated genome engineering. Cell 153: 910–918 23643243 10.1016/j.cell.2013.04.025 PMC3969854

[embr202051678-bib-0103] Wang J , Li BX , Ge PP , Li J , Wang Q , Gao GF , Qiu XB , Liu CH (2015) *Mycobacterium tuberculosis* suppresses innate immunity by coopting the host ubiquitin system. Nat Immunol 16: 237–245 25642820 10.1038/ni.3096

[embr202051678-bib-0104] Wang L , Liu Z , Wang J , Liu H , Wu J , Tang T , Li H , Yang H , Qin L , Ma D *et al* (2019) Oxidization of TGFbeta‐activated kinase by MPT53 is required for immunity to *Mycobacterium tuberculosis* . Nat Microbiol 4: 1378–1388 31110366 10.1038/s41564-019-0436-3

[embr202051678-bib-0105] Wang L , Wu J , Li J , Yang H , Tang T , Liang H , Zuo M , Wang J , Liu H , Liu F *et al* (2020) Host‐mediated ubiquitination of a mycobacterial protein suppresses immunity. Nature 577: 682–688 31942069 10.1038/s41586-019-1915-7

[embr202051678-bib-0106] Wang L , Zuo M , Chen H , Liu S , Wu X , Cui Z , Yang H , Liu H , Ge B (2017) *Mycobacterium tuberculosis* lipoprotein MPT83 induces apoptosis of infected macrophages by activating the TLR2/p38/COX‐2 signaling pathway. J Immunol 198: 4772–4780 28507027 10.4049/jimmunol.1700030

[embr202051678-bib-0107] Watson RO , Bell SL , MacDuff DA , Kimmey JM , Diner EJ , Olivas J , Vance RE , Stallings CL , Virgin HW , Cox JS (2015) The cytosolic sensor cGAS detects *Mycobacterium tuberculosis* DNA to induce type I interferons and activate autophagy. Cell Host Microbe 17: 811–819 26048136 10.1016/j.chom.2015.05.004 PMC4466081

[embr202051678-bib-0108] van der Wel N , Hava D , Houben D , Fluitsma D , van Zon M , Pierson J , Brenner M , Peters PJ (2007) *M. tuberculosis* and *M. leprae* translocate from the phagolysosome to the cytosol in myeloid cells. Cell 129: 1287–1298 17604718 10.1016/j.cell.2007.05.059

[embr202051678-bib-0109] Werninghaus K , Babiak A , Gross O , Holscher C , Dietrich H , Agger EM , Mages J , Mocsai A , Schoenen H , Finger K *et al* (2009) Adjuvanticity of a synthetic cord factor analogue for subunit *Mycobacterium tuberculosis* vaccination requires FcRgamma‐Syk‐Card9‐dependent innate immune activation. J Exp Med 206: 89–97 19139169 10.1084/jem.20081445 PMC2626670

[embr202051678-bib-0110] Wheat WH , Dhouib R , Angala SK , Larrouy‐Maumus G , Dobos K , Nigou J , Spencer JS , Jackson M (2015) The presence of a galactosamine substituent on the arabinogalactan of *Mycobacterium tuberculosis* abrogates full maturation of human peripheral blood monocyte‐derived dendritic cells and increases secretion of IL‐10. Tuberculosis 95: 476–489 26048627 10.1016/j.tube.2015.04.002 PMC4475442

[embr202051678-bib-0111] Wiersma VR , de Bruyn M , Helfrich W , Bremer E (2013) Therapeutic potential of Galectin‐9 in human disease. Med Res Rev 33(Suppl 1): E102–126 21793015 10.1002/med.20249

[embr202051678-bib-0112] Wolf AJ , Reyes CN , Liang W , Becker C , Shimada K , Wheeler ML , Cho HC , Popescu NI , Coggeshall KM , Arditi M *et al* (2016) Hexokinase is an innate immune receptor for the detection of bacterial peptidoglycan. Cell 166: 624–636 27374331 10.1016/j.cell.2016.05.076 PMC5534359

[embr202051678-bib-0113] Wolf AJ , Underhill DM (2018) Peptidoglycan recognition by the innate immune system. Nat Rev Immunol 18: 243–254 29292393 10.1038/nri.2017.136

[embr202051678-bib-0114] Wu Y , Xiong DC , Chen SC , Wang YS , Ye XS (2017) Total synthesis of mycobacterial arabinogalactan containing 92 monosaccharide units. Nat Commun 8: 14851 28300074 10.1038/ncomms14851 PMC5357306

[embr202051678-bib-0115] Yarkoni E , Rapp HJ (1977) Granuloma formation in lungs of mice after intravenous administration of emulsified trehalose‐6,6'‐dimycolate (cord factor): reaction intensity depends on size distribution of the oil droplets. Infect Immun 18: 552–554 924683 10.1128/iai.18.2.552-554.1977 PMC421268

[embr202051678-bib-0116] Yonekawa A , Saijo S , Hoshino Y , Miyake Y , Ishikawa E , Suzukawa M , Inoue H , Tanaka M , Yoneyama M , Oh‐hora M *et al* (2014) Dectin‐2 is a direct receptor for mannose‐capped lipoarabinomannan of mycobacteria. Immunity 41: 402–413 25176311 10.1016/j.immuni.2014.08.005

[embr202051678-bib-0117] Zhang Lu , Zhao Y , Gao R , Li J , Yang X , Gao Y , Zhao W , Gurcha SS , Veerapen N , Batt SM *et al* (2020) Cryo‐EM snapshots of mycobacterial arabinosyltransferase complex EmbB2‐AcpM2. Protein Cell 11: 505–517 32363534 10.1007/s13238-020-00726-6 PMC7305291

[embr202051678-bib-0118] Zhang Lu , Zhao Y , Gao Y , Wu L , Gao R , Zhang Qi , Wang Y , Wu C , Wu F , Gurcha SS *et al* (2020) Structures of cell wall arabinosyltransferases with the anti‐tuberculosis drug ethambutol. Science 368: 1211–1219 32327601 10.1126/science.aba9102

[embr202051678-bib-0119] Zhang X , Feng Y , Yao Q , He F (2017) Selection of a new *Mycobacterium tuberculosis* H37Rv aptamer and its application in the construction of a SWCNT/aptamer/Au‐IDE MSPQC H37Rv sensor. Biosens Bioelectron 98: 261–266 28689112 10.1016/j.bios.2017.05.043

[embr202051678-bib-0120] Zhao XQ , Zhu LL , Chang Q , Jiang C , You Y , Luo T , Jia XM , Lin X (2014) C‐type lectin receptor dectin‐3 mediates trehalose 6,6'‐dimycolate (TDM)‐induced Mincle expression through CARD9/Bcl10/MALT1‐dependent nuclear factor (NF)‐kappaB activation. J Biol Chem 289: 30052–30062 25202022 10.1074/jbc.M114.588574 PMC4208012

[embr202051678-bib-0121] Zheng R , Li Z , He F , Liu H , Chen J , Chen J , Xie X , Zhou J , Chen H , Wu X *et al* (2018) Genome‐wide association study identifies two risk loci for tuberculosis in Han Chinese. Nat Commun 9: 4072 30287856 10.1038/s41467-018-06539-w PMC6172286

[embr202051678-bib-0122] Zheng R , Liu H , Zhou Y , Yan D , Chen J , Ma D , Feng Y , Qin L , Liu F , Huang X *et al* (2018) Notch4 negatively regulates the inflammatory response to *Mycobacterium tuberculosis* infection by inhibiting TAK1 activation. J Infect Dis 218: 312–323 29228365 10.1093/infdis/jix636

