# Supplementary material for: Sensing of mycobacterial arabinogalactan by galectin‐9 exacerbates mycobacterial infection
Source: EMBO Rep. 2021 May 13;22(7):e51678. doi: 10.15252/embr.202051678 (PMC8256295; doi:10.15252/embr.202051678)
Supplement: Supplementary file 1 — Appendix [file EMBR-22-e51678-s002.pdf]

Appendix

Sensing of mycobacterial arabinogalactan by galectin-9 exacerbates mycobacterial infection

Xiangyang Wu[1,†], Yong Wu[2,†], Ruijuan Zheng[1,†], Fen Tang[1], Lianhua Qin[1], Detian Lai[1], Lu Zhang[3], Lingming Chen[4], Bo Yan[5], Hua Yang[1], Yang Wang[1], Feifei Li[2], Jinyu Zhang[3], Fei Wang[1], Lin Wang[1], Yajuan Cao[1], Mingtong Ma[1], Zhonghua Liu[1], Jianxia Chen[1], Xiaochen Huang[1], Jie Wang[1], Ruiliang Jin[1], Peng Wang[6], Qin Sun[6], Wei Sha[6], Liangdong Lyu[7], Pedro Moura-Alves[8,10], Anca Dorhoi[8,9], Gang Pei[8], Peng Zhang[11], Jiayu Chen[12], Shaorong Gao[12], Felix Randow[13], Gucheng Zeng[4], Chang Chen[11], Xin-Shan Ye[2,*], Stefan HE Kaufmann[8,14,15*], Haipeng Liu[1,8,16,17,*], Baoxue Ge[1,16,18*]

Table of contents

Appendix Figure S1. AG induces lung damage........2

Appendix Figure S2. AG aptamer moderately enhances the survival of mycobacteria-infected mice and zebrafish........3

Appendix Figure S3. AG contributes to mycobacteria-induced MMPs expression........5

Appendix Figure S4. Diagram showing the sensing of AG by galectin-9........7

Appendix Table S1. Sequences of primers for quantitative RT-PCR........8

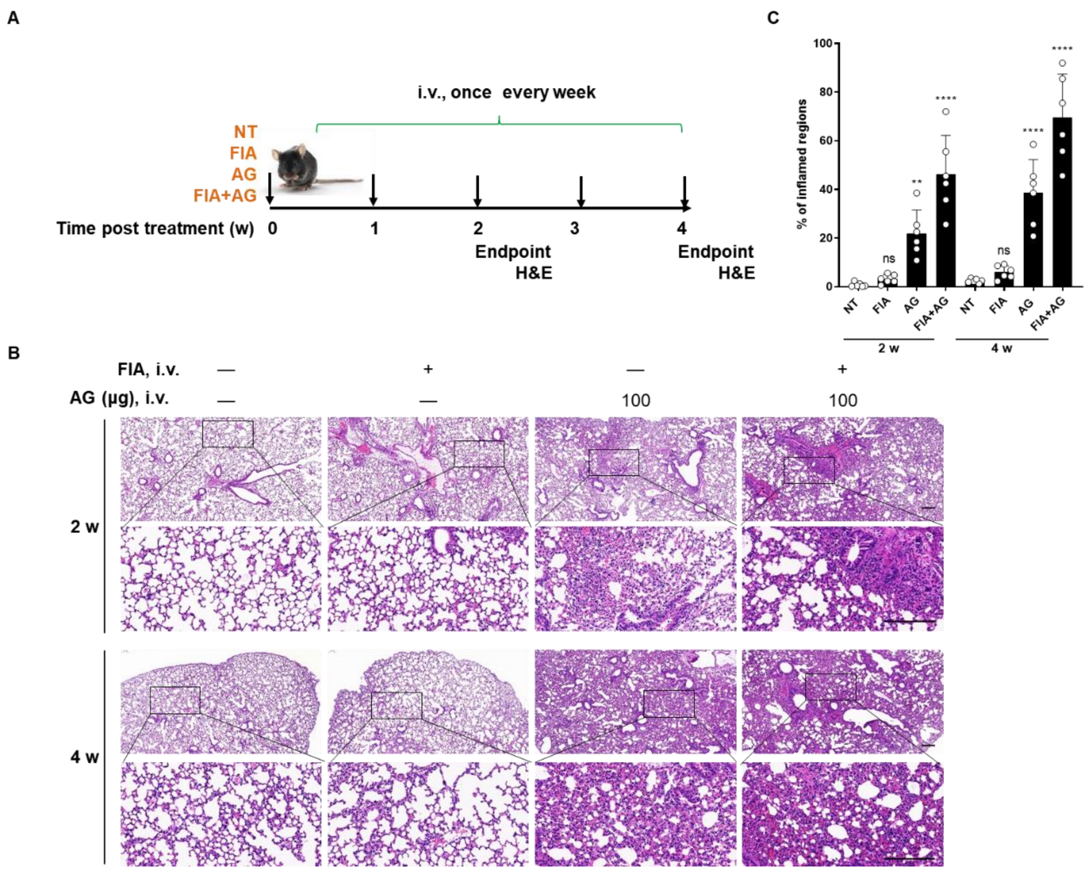

Appendix Figure S1. AG induces lung damage.

(A) Diagram showing the procedure for intravenous administration of AG, Freund's incomplete adjuvant (FIA) or emulsified AG+FIA.NT, not treated.

(B, C) H&E staining of lung sections from mice intravenously treated with AG, FIA, emulsified AG+FAI or left untreated (**B**). Quantification of lung inflamed regions shown in (**C**).

Data information: Data are representative of n=3 independent experiments. Data in (**C**) are means + SD of indicated mice from 1 of n=3 independent experiments and each symbol represents data from 1 mouse. One-way ANOVA followed by Dunnett's post hoc test (**C**) was used for statistical analysis. ns, not significant; **, p<0.01; ****, p<0.0001. Scale bar, 200 μm.

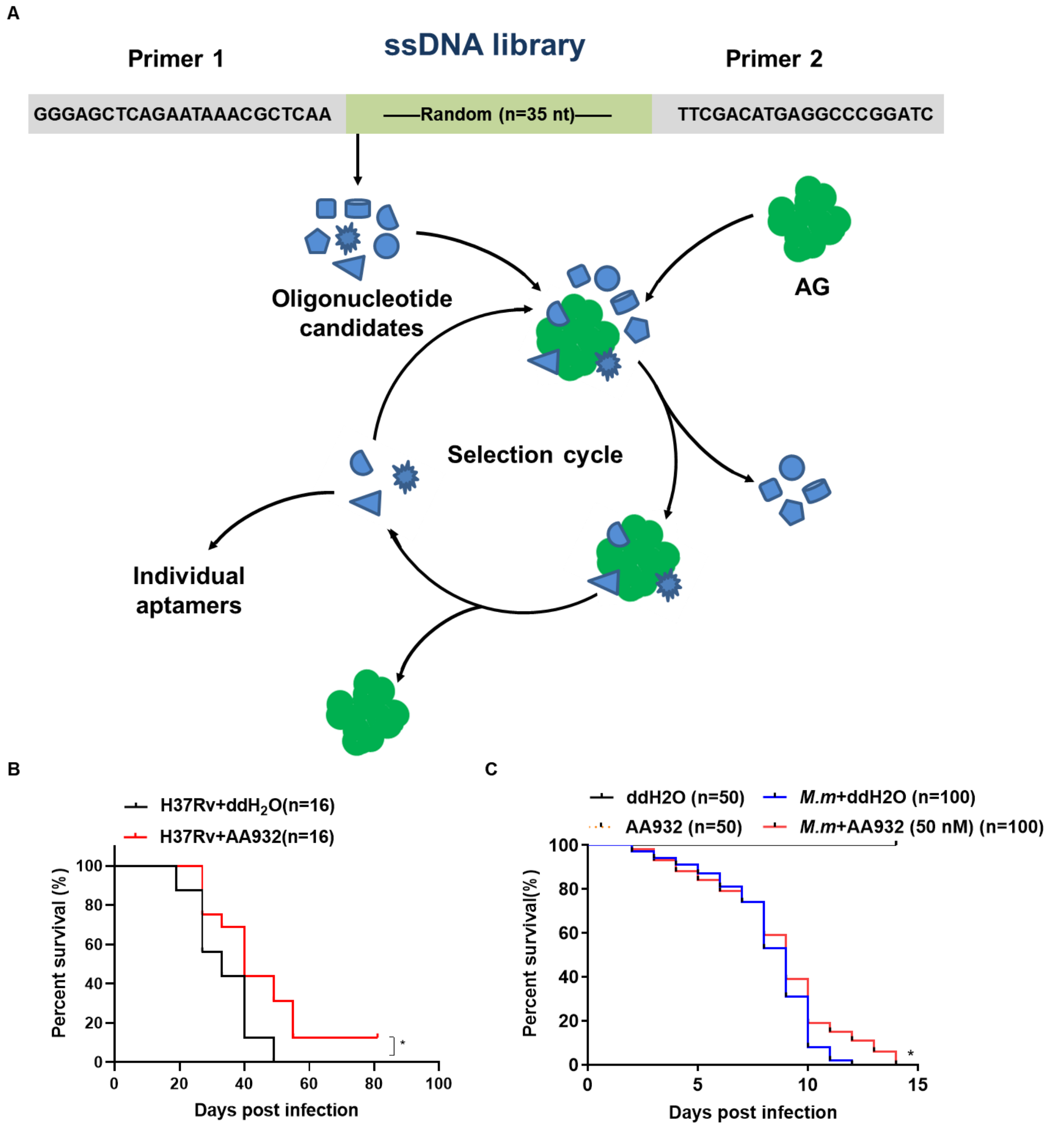

Appendix Figure S2. AG aptamer moderately enhances the survival of mycobacteria-infected mice and zebrafish

(A) Flow chart of the strategy for the screening of aptamers against AG by SELEX selection.

(B) Infected mice were monitored for survival (Kaplan–Meier curves). SCID mice infected with lethal dose of *Mtb* H37Rv in the absence or presence of intranasally administrated AG aptamers (1 µg) once at a 1 week-interval for indicated times (n=16 mice per group).

(C) Infected zebrafish were monitored for survival (Kaplan–Meier curves). Zebrafish larvae were left uninfected or infected with *M. marinum* via Duct of Cuvier (n=50 for uninfected groups and n=100 for infected groups) in the absence or presence of AG aptamer AA932 (2 ng/500 CFU).

Data information: Data are representative of n=3 independent experiments. One-way ANOVA followed by Gehan–Breslow–Wilcoxon test (**B** and **C**) were used for statistical analysis. *, $p<0.05$.

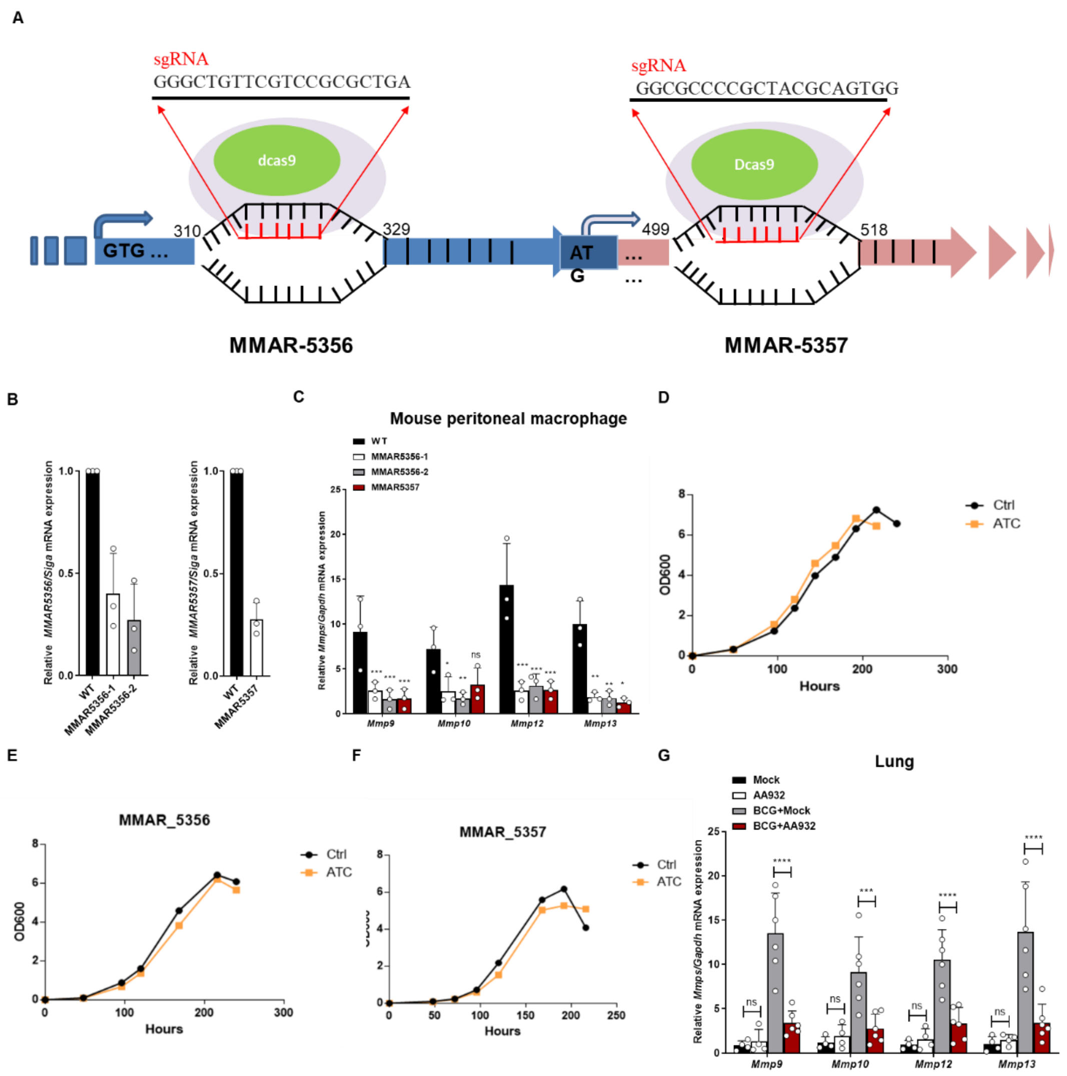

Appendix Figure S3. AG contributes to mycobacteria-induced MMPs expression.

(A) Diagram showing the sequence of sgRNA and the targeting region of *MMAR_5356* and *MMAR_5357*, respectively.

(B) qPCR analysis of *MMAR_5356* and *MMAR_5357* transcripts in the parental *M. marinum* and derivative *MMAR_5356* and *MMAR_5357* knockdown *M. marinum* cultured in 7H9 broth with 100 ng/ml ATc (anhydrotetracycline) from 0.1 OD_{600} to 0.8-1.0 OD_{600}.

(C) qPCR analysis of *Mmps* including *Mmp9*, *Mmp10*, *Mmp12* and *Mmp13* from peritoneal macrophages infected with parental *M. marinum* and derivative

MMAR-5356 and *MMAR-5357* knockdown *M. marinum* prepared as described in (**B**) in the presence of 100 ng/ml ATc for 12 h.

(D-F) Growth curve of WT *M. marinum* (**D**), *MMAR_5356* (**E**) and *MMAR_5357* (**F**) in 7H9 culture under regular condition in absence or presence of 100 ng/ml ATc.

(G) qPCR analysis of *Mmps* including *Mmp9*, *Mmp10*, *Mmp12* and *Mmp13* from the lungs of mice intranasally infected with *M. bovis* BCG for 4 weeks in absence of presence of AG aptamers. AG aptamers (1 μg/mouse) were intranasally administrated once at a 1 week-interval.

Data information: Data in (**B, C**) are means + SD averaged from 3 independent experiments performed with technical triplicates and each symbol represents the mean of technical triplicates. Data in (**G**) are means + SD of indicated numbers of mice from 1 of n=3 independent experiments and each symbol represents data from 1 mouse. One-way ANOVA followed by Dunnett's post hoc test were used for statistical analysis. ns, not significant; *, p<0.05; **, p<0.01; ***, p<0.001; ****, p<0.0001.

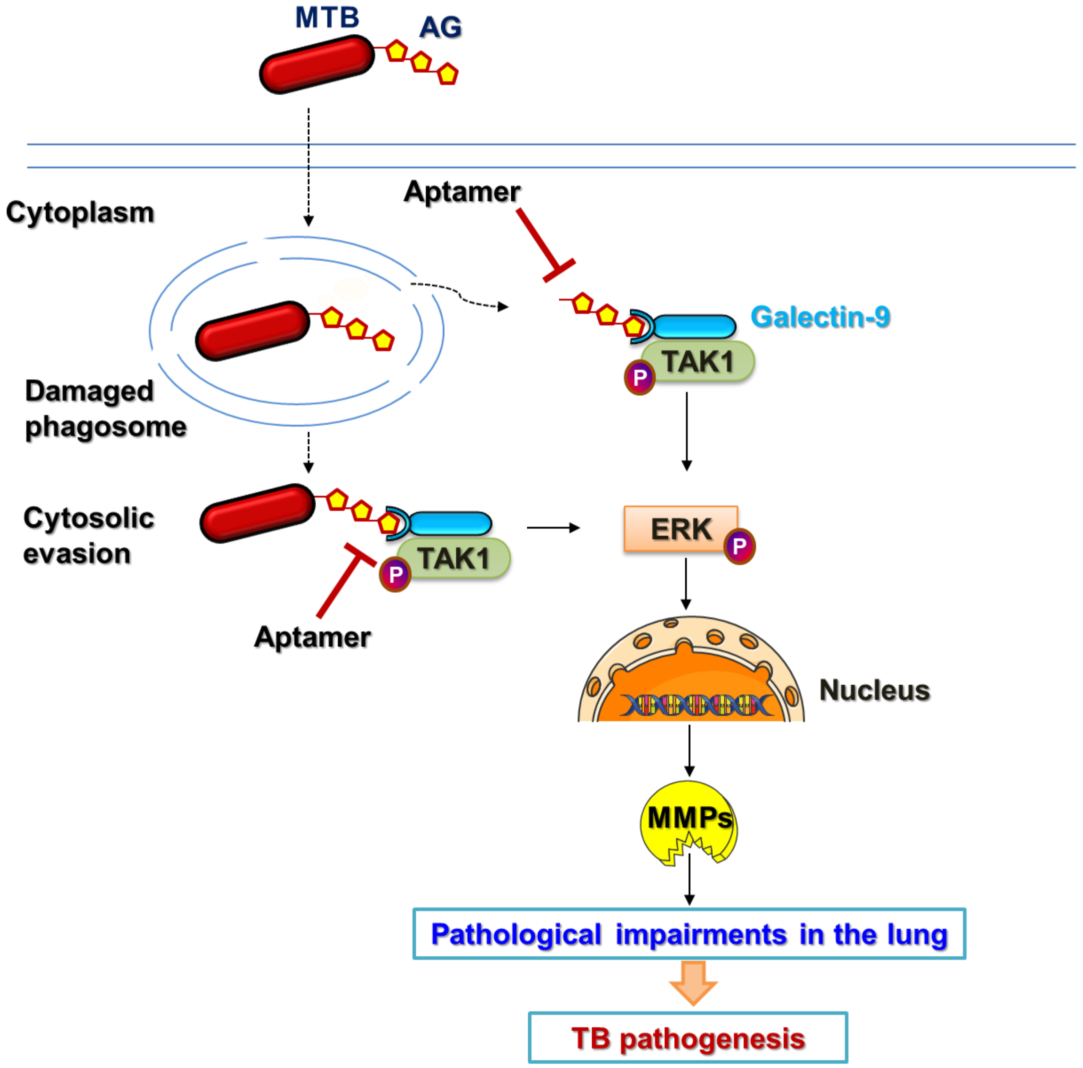

Appendix Figure S4. Diagram showing the sensing of AG by galectin-9. The harsh environment in phagosome or lysosome may lead to the shedding of AG from *Mtb* cell wall and the leakage of AG into the cytosol via the damaged membrane. *Mtb* may also escape into the cytosol and the replicating bacteria may expose the newly synthesized AG. Engagement of galectin-9 by mycobacterial AG led to the activation of TAK1-ERK-MMPs signaling axis, which is critical for the induction of pathological impairments in the lung and the exacerbation of TB pathogenesis. The development of aptamer targeting AG successfully abrogates AG-induced activation of TAK1-ERK-MMPs signaling axis and attenuates mycobacterial infection, providing the basis for the host-directed therapy of TB by targeting AG-galectin-9 interface.

Appendix Table S1. Sequences of primers for quantitative RT-PCR

**Gene name**	**Gene ID**	**Forward**	**Reverse**
m*Mmp2*	17390	CAAGTTCCCCGGCGATGTC	TTCTGGTCAAGGTCACCTGTC
m*Mmp9*	17395	CTGGACAGCCAGACACTAAAG	CTCGCGGCAAGTCTTCAGAG
m*Mmp10*	17384	GAGCCACTAGCCATCCTGG	CTGAGCAAGATCCATGCTTGG
m*Mmp12*	17381	CTGCTCCCATGAATGACAGTG	AGTTGCTTCTAGCCCAAAGAAC
m*Mmp13*	17386	CTTCTTCTTGTTGAGCTGGACTC	CTGTGGAGGTCACTGTAGACT
h*MMP9*	4318	TGGCAGAAGAGATGGTTGT	GCAGGCATAAGAGGAGTGA
h*MMP10*	4319	GGCTTCTGCTGTATCATT	AGTCAATAAGTTCGTCTATATCT
h*MMP12*	4321	AGGTGAACCAAGAACATAC	CCAGATTGACCAACAGAA
hMMP13	4322	ACTGAGAGGCTCCGAGAAATG	GAACCCCGCATCTTGGCTT
