# Supplementary material for: Sensing of mycobacterial arabinogalactan by galectin‐9 exacerbates mycobacterial infection
Source: EMBO Rep. 2021 May 13;22(7):e51678. doi: 10.15252/embr.202051678 (PMC8256295; doi:10.15252/embr.202051678)
Supplement: Supplementary file 2 — Expanded View Figures PDF [file EMBR-22-e51678-s005.pdf]

Expanded View Figures

Figure EV1. AG induces expression of MMPs.

A Scatter plots of differentially expressed genes in the mouse peritoneal macrophages stimulated with AG (1 μg/ml) for 24 h as identified by RNA-seq analysis. The RNA from the peritoneal macrophages was pooled and subjected to RNA-seq.

B GO class of gene expressions in mouse peritoneal macrophages stimulated with AG (1 μg/ml) for 24 h as identified by RNA-seq analysis.

C KEGG class of gene expressions in mouse peritoneal macrophages stimulated with AG (1 μg/ml) for 24 h as identified by RNA-seq analysis.

D, E qPCR analysis of *Mmps* including *Mmp9*, *Mmp10*, and *Mmp12* mRNA from THP-1 cells stimulated with AG (1 μg/ml) for indicated times (D) or at indicated concentrations (μg/ml) for 48 h (E).

F qPCR analysis of *Mmps* including *Mmp9*, *Mmp10*, *Mmp12*, and *Mmp13* mRNA from mouse peritoneal macrophages stimulated with AG at indicated concentrations (μg/ml) for 24 h.

G qPCR analysis of *Mmps* including *Mmp9*, *Mmp10*, *Mmp12*, and *Mmp13* from the lungs of mice at indicated concentrations (μg) for 3 days post-intraperitoneal administration of AG.

H qPCR analysis of *Mmps* including *Mmp9*, *Mmp10*, and *Mmp12* from THP-1 cells stimulated with AG (1 μg/ml) for 48 h left untreated or pretreated with AG aptamers AA932 or AA835 (0.5 μg/ml).

Data information: Data in (D–F, H) are means ± SD averaged from 3 independent experiments performed with technical triplicates, and each symbol represents the mean of technical triplicates. Data in (G) are means ± SD of indicated mice from 1 of $n = 3$ independent experiments, and each symbol represents data from 1 mouse. One-way ANOVA followed by Dunnett's *post hoc* test were used for statistical analysis, respectively. ns, not significant; $^{**}P < 0.01$; $^{***}P < 0.001$; $^{****}P < 0.0001$.

 © 2021 The Authors

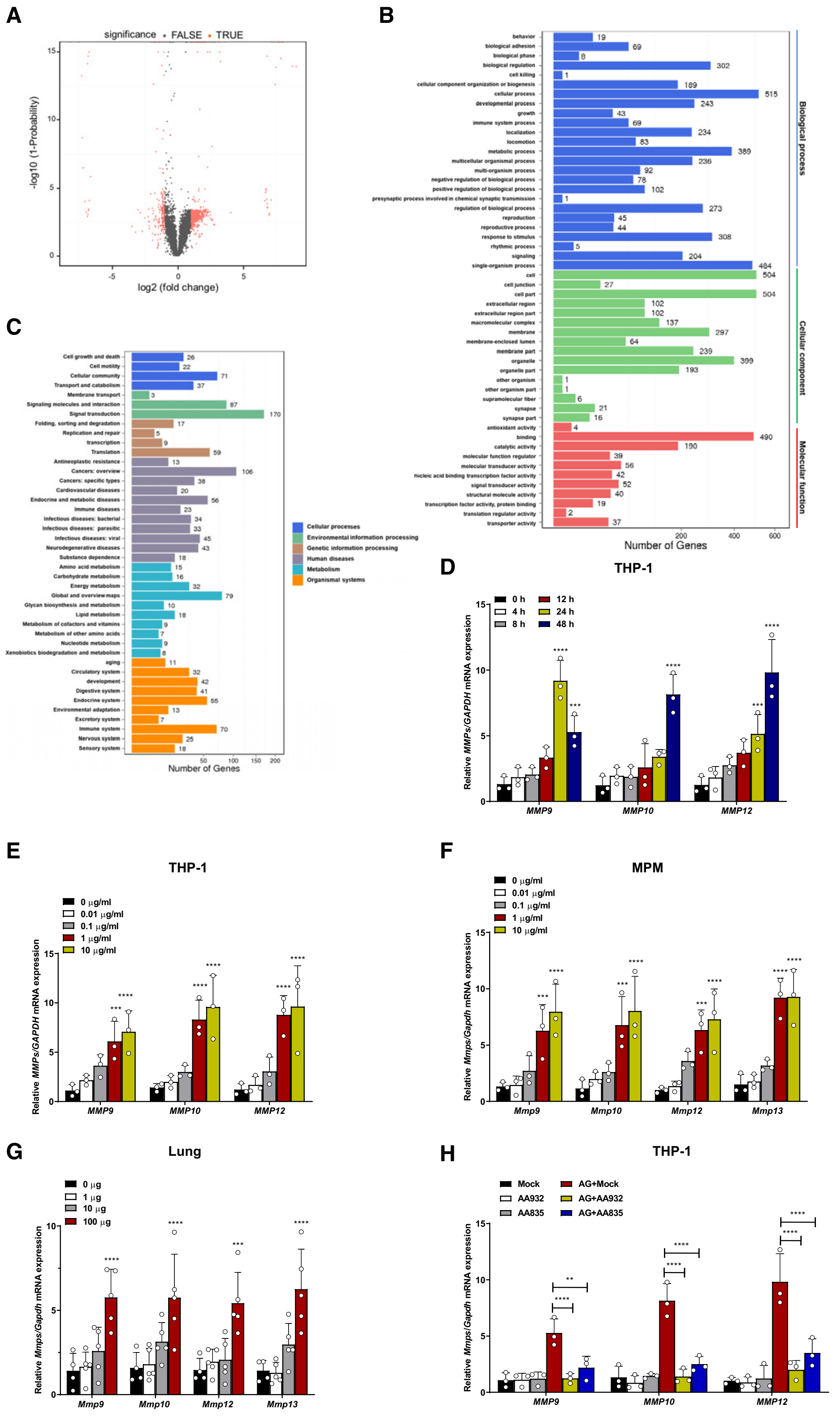
A
significance • FALSE • TRUE
-log10 (1-Probability)
log2 (fold change)
B
Number of Genes
Biological process
Cellular component
Molecular function
C
Number of Genes
Cellular processes
Environmental information processing
Genetic information processing
Human diseases
Metabolism
Organismal systems
D
THP-1
0 h
4 h
8 h
12 h
24 h
48 h
Relative MMPs/GAPDH mRNA expression
MMP9
MMP10
MMP12
E
THP-1
0 µg/ml
0.01 µg/ml
0.1 µg/ml
1 µg/ml
10 µg/ml
Relative MMPs/GAPDH mRNA expression
MMP9
MMP10
MMP12
F
MPM
0 µg/ml
0.01 µg/ml
0.1 µg/ml
1 µg/ml
10 µg/ml
Relative Mmps/Gapdh mRNA expression
Mmp9
Mmp10
Mmp12
Mmp13
G
Lung
0 µg
1 µg
10 µg
100 µg
Relative Mmps/Gapdh mRNA expression
Mmp9
Mmp10
Mmp12
Mmp13
H
THP-1
Mock
AA932
AA835
AG+Mock
AG+AA932
AG+AA835
Relative Mmps/Gapdh mRNA expression
MMP9
MMP10
MMP12

Figure EV1.

Figure EV2. Interaction of AG with galectins.

A Coomassie blue staining of galectin-1, galectin-3, galectin-7, galectin-8, galectin-9, galectin-14, and galectin-related protein (LGALSL) post–SDS–PAGE analysis.
B Chemical structures and conformations of β-galactofuranoside and β-galactopyranoside.
C–I SPR assay of interactions of AG with indicated galectins including galectin-1 (C), galectin-3 (D), galectin-7 (E), galectin-8 (F), galectin-14 (G), LGASL (H), and a summary table of KD (I). Curve fittings to a 1:1 Langmuir-binding model calculated with TraceDrawer are shown as smooth black lines. The binding affinity of galectin-9 and CRD2 to AG is highlighted in (I) in red.
J SPR assay of interactions of AG with CRD1 of galectin-9.
K Coomassie blue staining of galectin-9(1–146) and CRD2 of galectin-9 post–SDS–PAGE analysis.
L SPR assay of interactions of AG with galectin-9(1–146).

 © 2021 The Authors

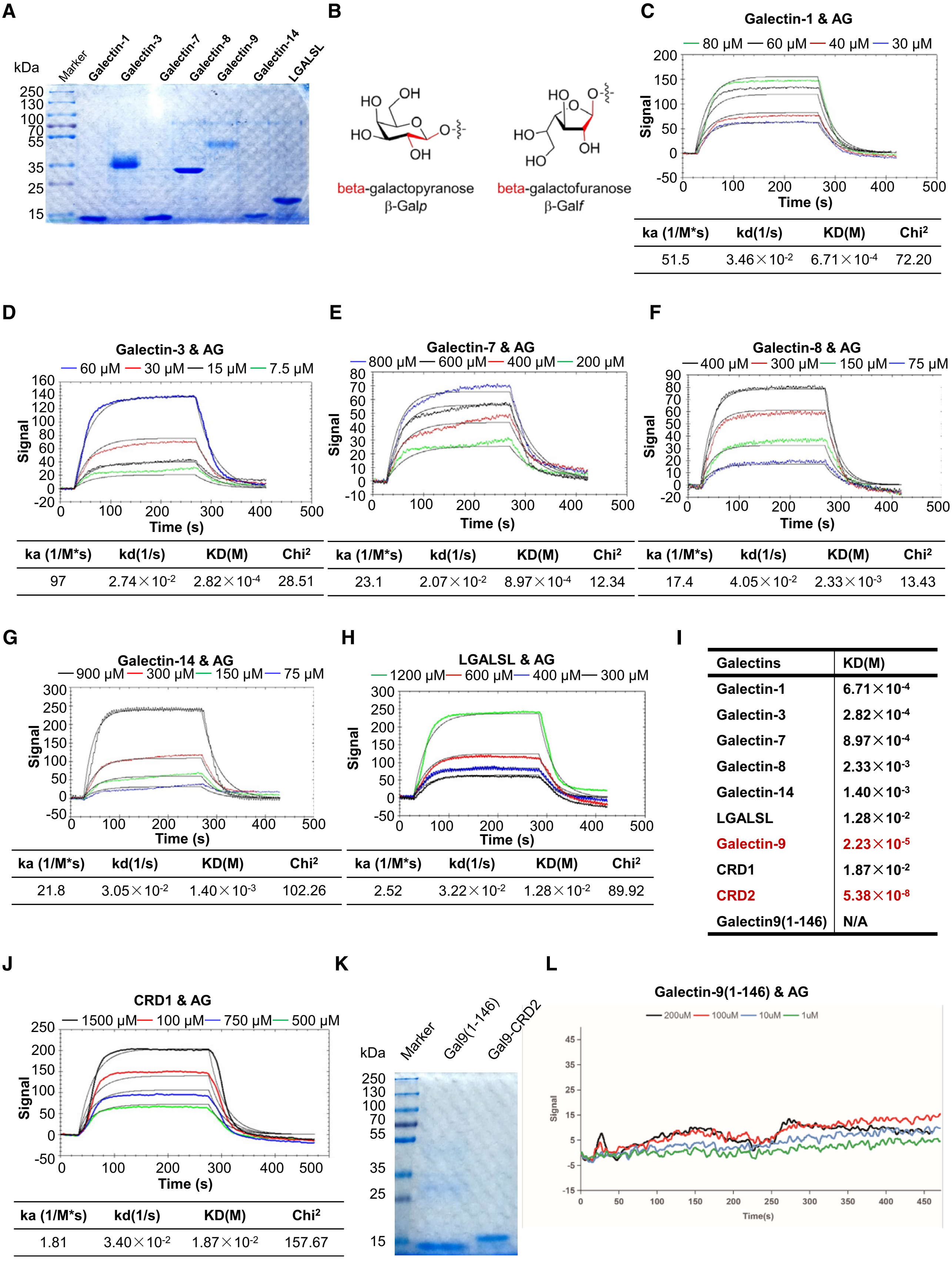

C

ka (1/M*s)	kd(1/s)	KD(M)	Chi²
51.5	3.46×10^{-2}	6.71×10^{-4}	72.20

D

ka (1/M*s)	kd(1/s)	KD(M)	Chi²
97	2.74×10^{-2}	2.82×10^{-4}	28.51

E

ka (1/M*s)	kd(1/s)	KD(M)	Chi²
23.1	2.07×10^{-2}	8.97×10^{-4}	12.34

F

ka (1/M*s)	kd(1/s)	KD(M)	Chi²
17.4	4.05×10^{-2}	2.33×10^{-3}	13.43

G

ka (1/M*s)	kd(1/s)	KD(M)	Chi²
21.8	3.05×10^{-2}	1.40×10^{-3}	102.26

H

ka (1/M*s)	kd(1/s)	KD(M)	Chi²
2.52	3.22×10^{-2}	1.28×10^{-2}	89.92

I

Galectins	KD(M)
Galectin-1	6.71×10^{-4}
Galectin-3	2.82×10^{-4}
Galectin-7	8.97×10^{-4}
Galectin-8	2.33×10^{-3}
Galectin-14	1.40×10^{-3}
LGALSL	1.28×10^{-2}
Galectin-9	2.23×10^{-5}
CRD1	1.87×10^{-2}
CRD2	5.38×10^{-8}
Galectin9(1-146)	N/A

J

ka (1/M*s)	kd(1/s)	KD(M)	Chi²
1.81	3.40×10^{-2}	1.87×10^{-2}	157.67

Figure EV2.

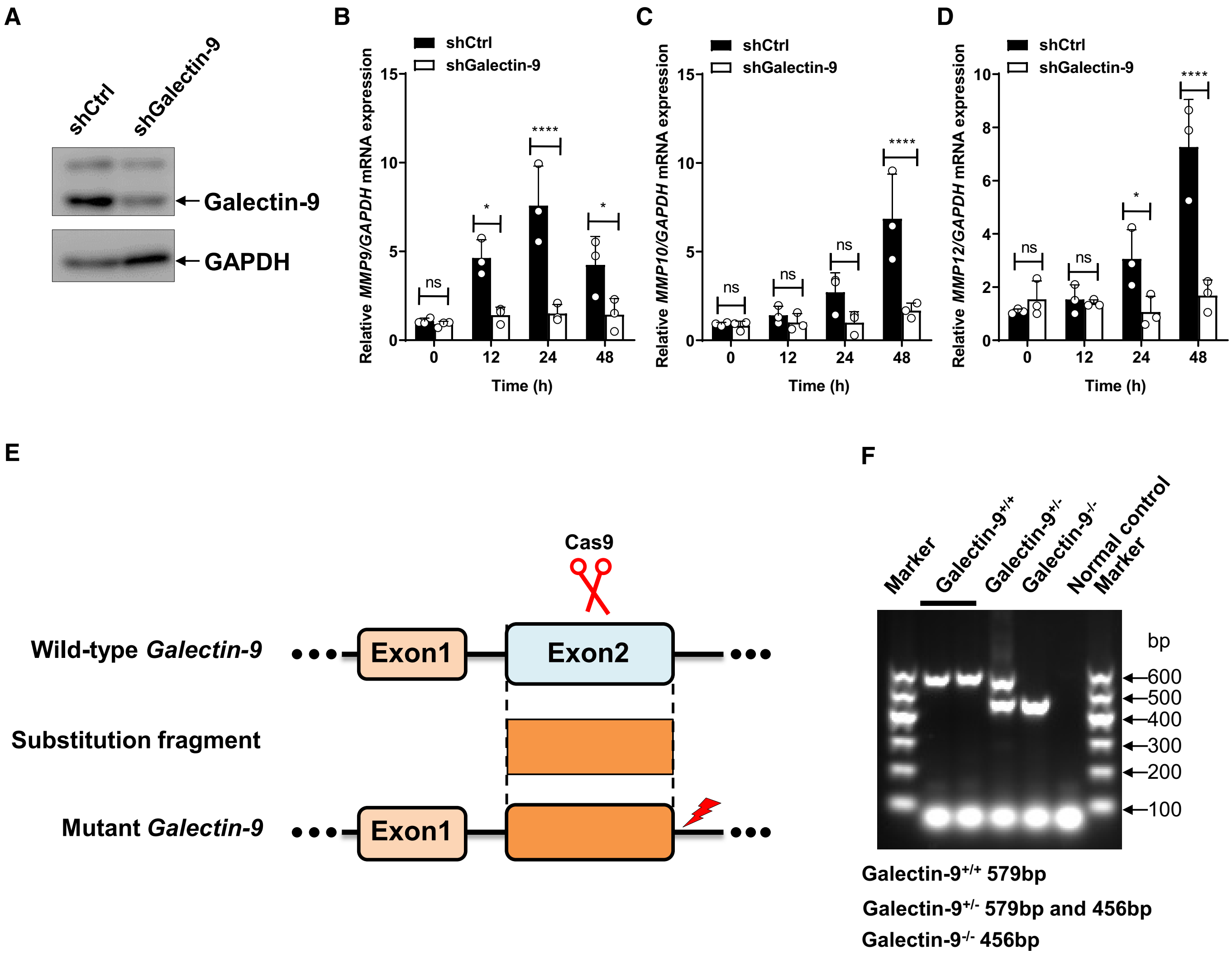

Figure EV3. Galectin-9 is essential for AG-induced expression of MMPs.

A Immunoblots of cell lysates of THP-1 cells stably transfected with scrambled shRNA or shRNA targeting galectin-9.

B–D qPCR analysis of *Mmps* including *Mmp9* (B), *Mmp10* (C), and *Mmp12* (D) mRNA from control or Galectin-9 knockdown THP-1 cells stimulated with AG (1 μg/ml) for indicated times.

E Diagram showing the gRNA-targeting genome sites.

F Identification of galectin-9 KO mice with PCR.

Data information: Data in (B–D) are means ± SD averaged from $n = 3$ independent experiments performed with technical triplicates, and each symbol represents the mean of technical triplicates. Data in (A, F) are representative of at least $n = 2$ independent experiments. Two-way ANOVA followed by Tukey's *post hoc* test (B-D) was used for statistical analysis, respectively. ns, not significant; $*P < 0.05$; $****P < 0.0001$.
Source data are available online for this figure.

 © 2021 The Authors

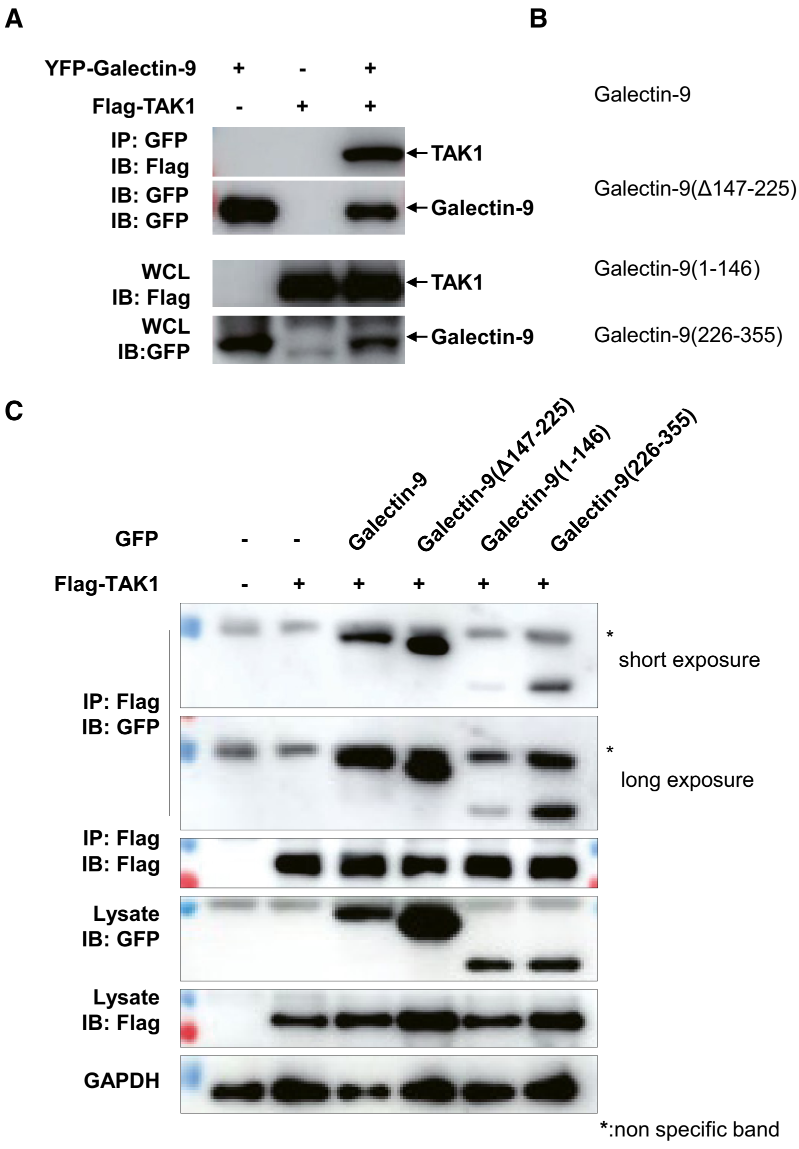

Figure EV4. Interaction of Galectin-9 with TAK1.

A Immunoblots and immunoprecipitation analysis of lysates of HEK293T cells transfected with various plasmids as indicated.
B Diagram showing various constructs of plasmids including Galectin-9, Galectin-9(Δ147–225), Galectin-9(1–146), and Galectin-9(226–355).
C Immunoblots and immunoprecipitation of lysates from HEK293T cells transfected with plasmids as indicated.

Source data are available online for this figure.

© 2021 The Authors

Figure EV5. AG induces MMPs via TAK1 activation.

A Immunoblots of cell lysates of peritoneal macrophages stimulated with AG (1 μg/ml) in the absence or presence of TAK1 inhibitor 5Z-7-OZ (1 μM) for indicated times. Data are representative of $n = 3$ independent experiments.

B qPCR analysis of *Mmps* including *Mmp9*, *Mmp10*, *Mmp12*, and *Mmp13* from peritoneal macrophages left unstimulated (NT) or stimulated with AG (1 μg/ml) in the absence or presence of TAK1 inhibitor 5Z-7-OZ (1 μM) for 24 h.

C Immunoblots of cell supernatants to analyze secreted MMP9, MMP10, MMP12, and MMP13 by mouse peritoneal macrophages stimulated with AG (1 μg/ml) for indicated times in the absence or presence of TAK1 inhibitor 5Z-7-OZ (1 μM); GADPH of cell lysates served as the loading control.

D Immunoblots of cell supernatants to analyze secreted MMP9, MMP10, MMP12, and MMP13 by mouse peritoneal macrophages infected with H37Rv for indicated times (MOI = 5) in the absence or presence of TAK1 inhibitor 5Z-7-OZ (1 μM); GADPH of cell lysates served as the loading control.

E Immunoblots of cell lysates of HEK293T cells stimulated with AG (1 μg/ml) for the indicated time to analyze p-ERK1/2 and p-TAK1(T187). GADPH of cell lysates is shown as loading control.

F Immunoblots of lysates of HEK293T cells stimulated with AG (1 μg/ml) for 3 h after transfection of the indicated plasmids for 48 h.

Data information: Data in (B) are means ± SD averaged from 3 independent experiments performed with technical triplicates and each symbol represents the mean of technical triplicates. Two-way ANOVA followed by Dunnett's *post hoc* test were used for statistical analysis. ns, not significant; $^{**}P < 0.01$; $^{****}P < 0.0001$.
Source data are available online for this figure.

© 2021 The Authors

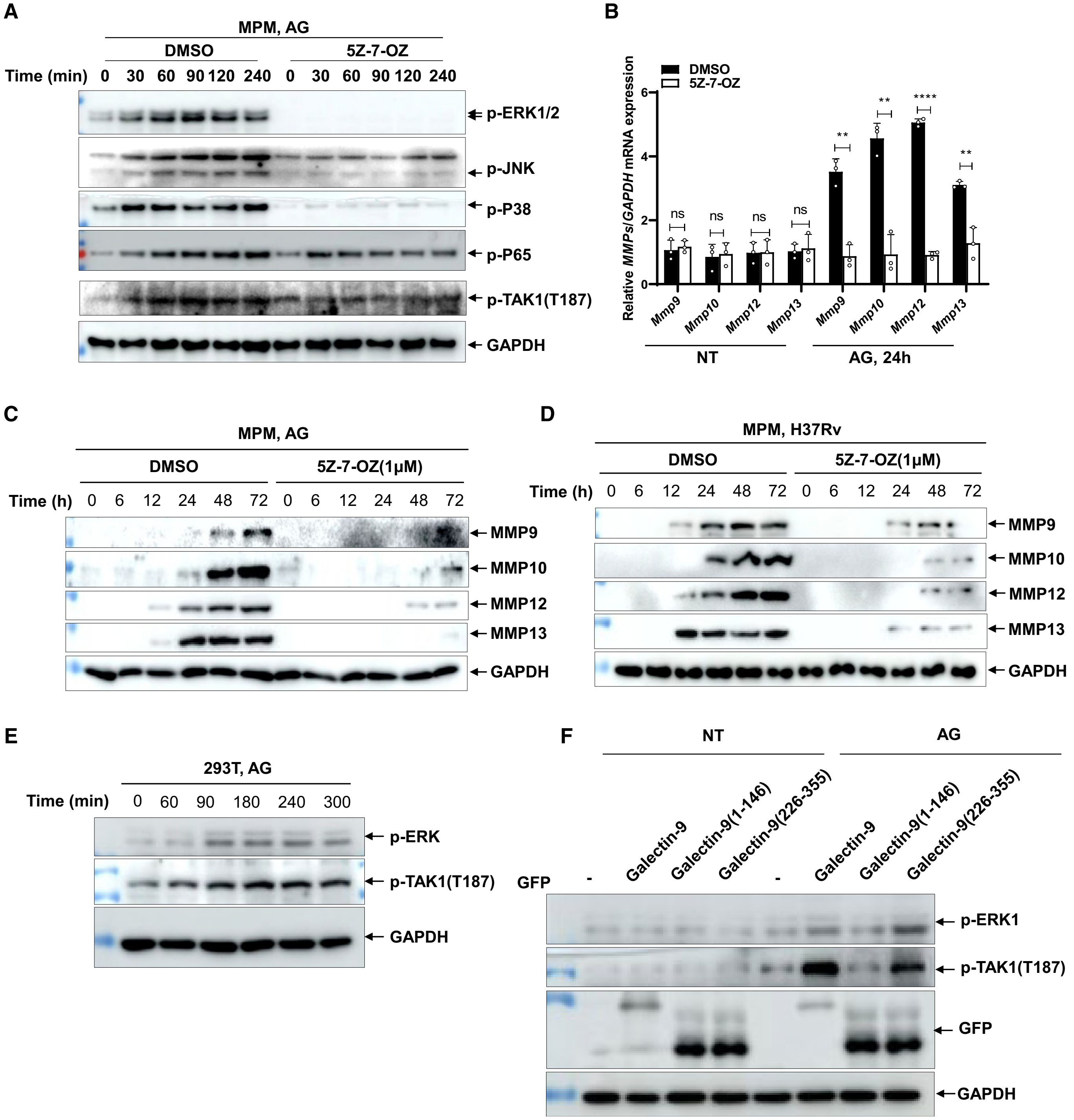
A
MPM, AG
DMSO
5Z-7-OZ
Time (min) 0 30 60 90 120 240 0 30 60 90 120 240
p-ERK1/2
p-JNK
p-P38
p-P65
p-TAK1(T187)
GAPDH
B
DMSO
5Z-7-OZ
Relative MMPs/GAPDH mRNA expression
ns
**
****
Mmp9
Mmp10
Mmp12
Mmp13
NT
AG, 24h
C
MPM, AG
DMSO
5Z-7-OZ(1μM)
Time (h) 0 6 12 24 48 72 0 6 12 24 48 72
MMP9
MMP10
MMP12
MMP13
GAPDH
D
MPM, H37Rv
DMSO
5Z-7-OZ(1μM)
Time (h) 0 6 12 24 48 72 0 6 12 24 48 72
MMP9
MMP10
MMP12
MMP13
GAPDH
E
293T, AG
Time (min) 0 60 90 180 240 300
p-ERK
p-TAK1(T187)
GAPDH
F
NT
AG
GFP
-
Galectin-9
Galectin-9(1-146)
Galectin-9(226-355)
p-ERK1
p-TAK1(T187)
GFP
GAPDH

Figure EV5.
