# Supplementary figures and images for: Sensing of mycobacterial arabinogalactan by galectin‐9 exacerbates mycobacterial infection
Source: EMBO Rep. 2021 May 13;22(7):e51678. doi: 10.15252/embr.202051678 (PMC8256295; doi:10.15252/embr.202051678)

Figure EV3

A

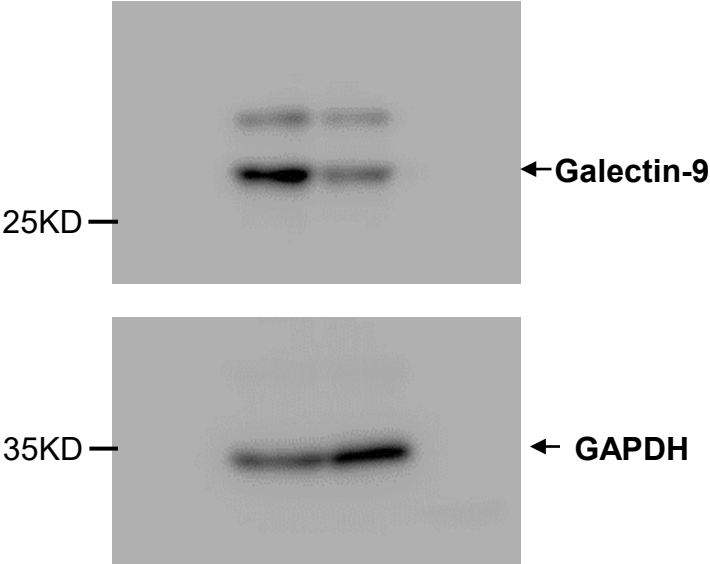

Supplement: Supplementary file 3 — Source Data for Expanded View [file EMBR-22-e51678-s001.zip › embr202051678-sup-0007-SDataEV/EV-Figure_Source_Data/Figure_EV3_Source_Data.pdf]

Figure EV4

A

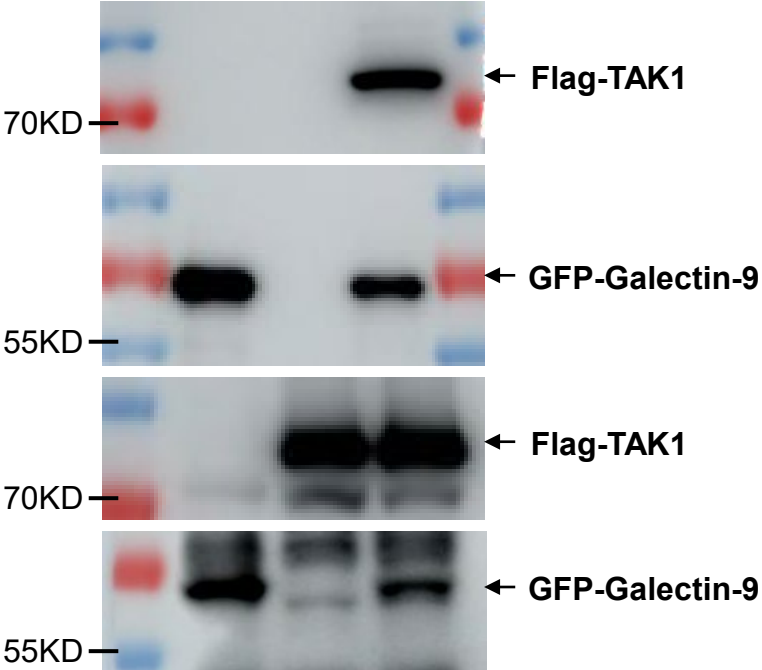

C

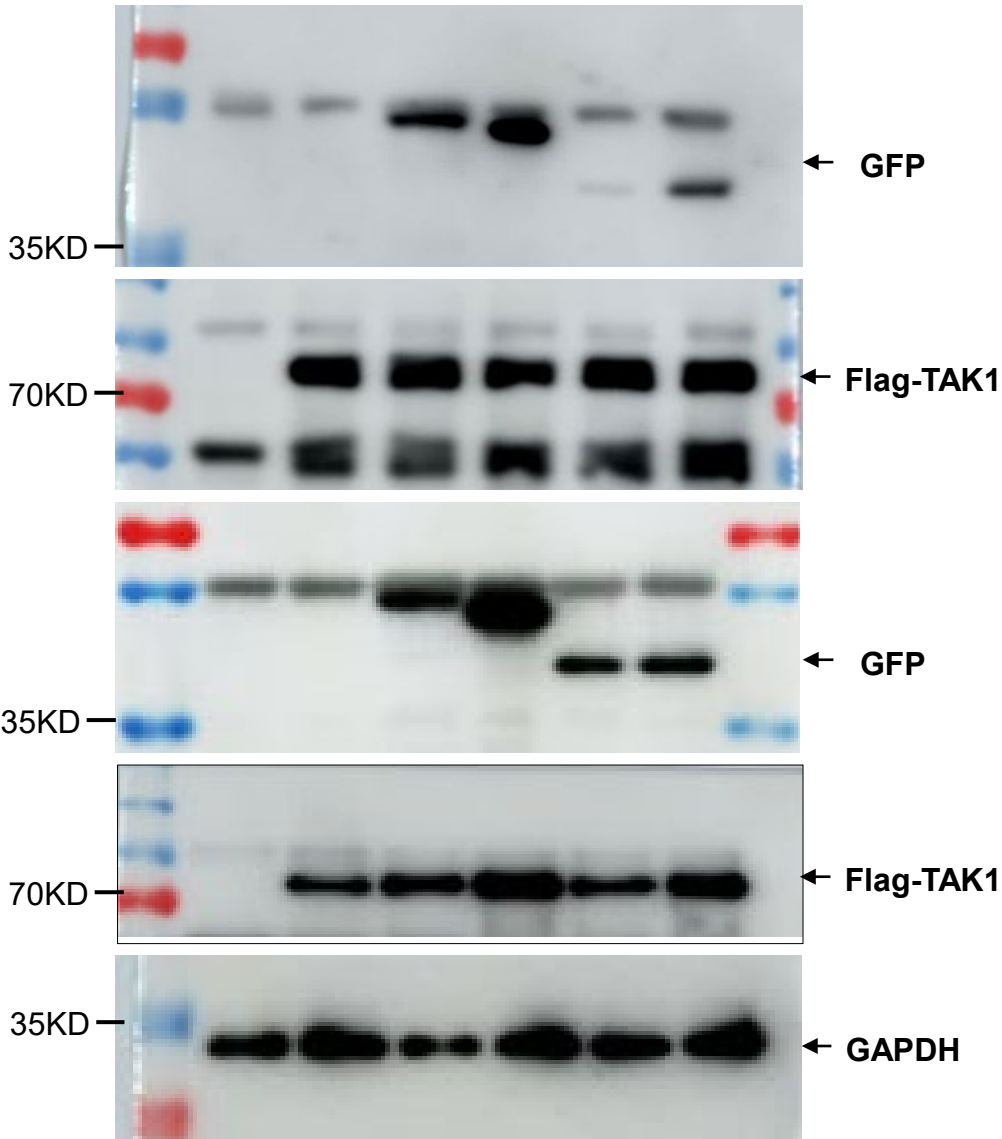

Supplement: Supplementary file 3 — Source Data for Expanded View [file EMBR-22-e51678-s001.zip › embr202051678-sup-0007-SDataEV/EV-Figure_Source_Data/Figure_EV4_Source_Data.pdf]

Figure EV5

A

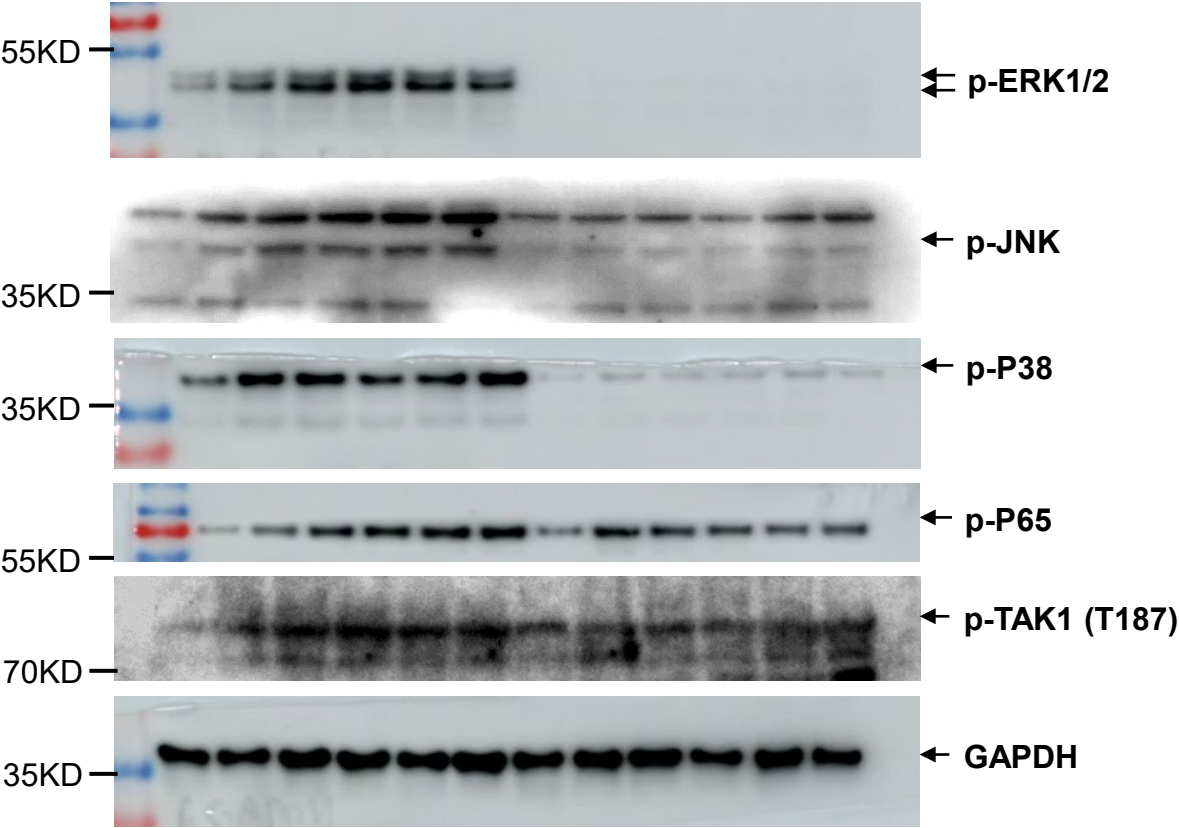

C

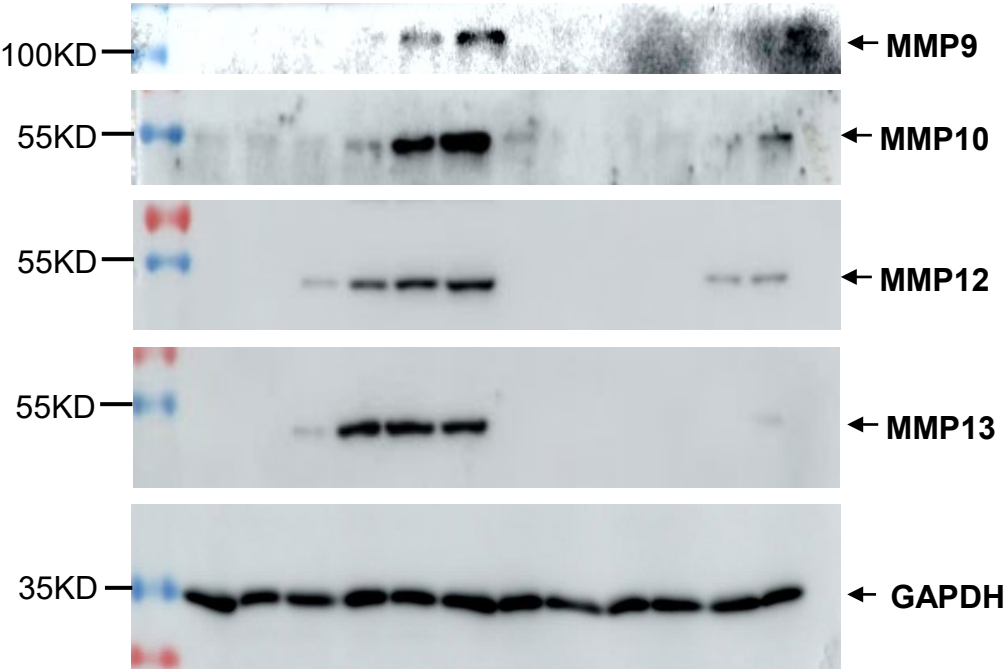

Figure EV5

D

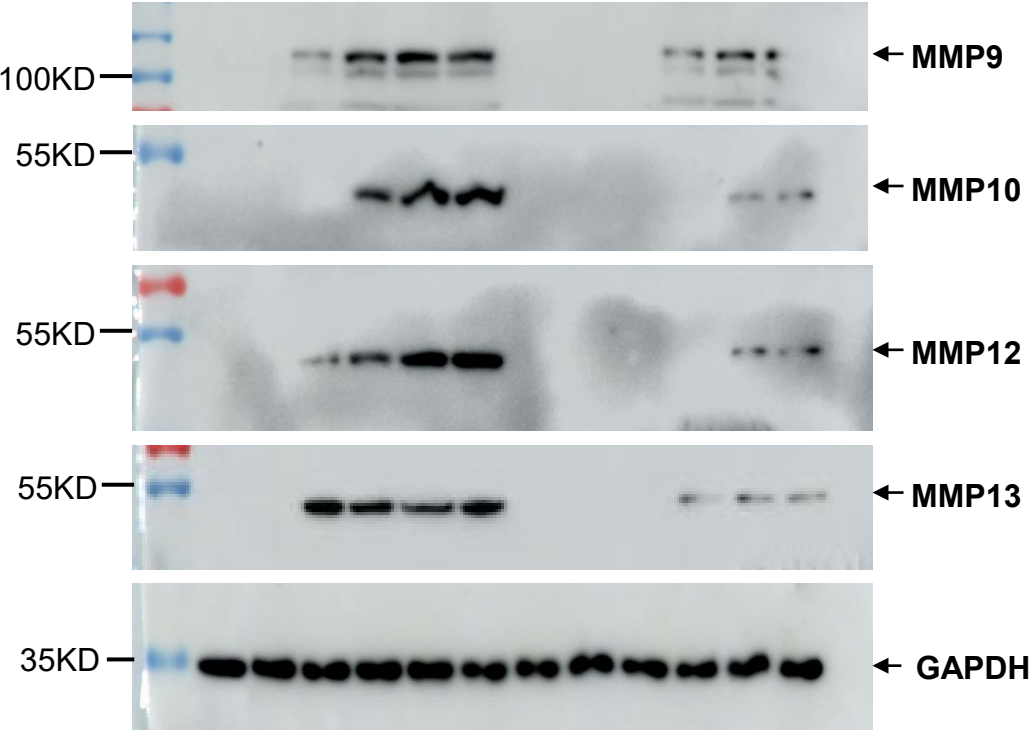

E

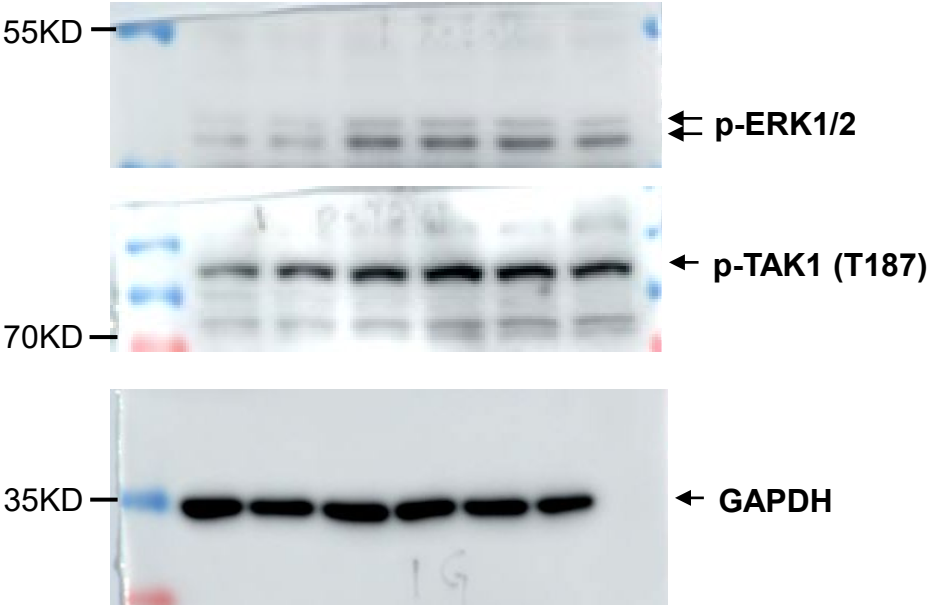

Figure EV5

F

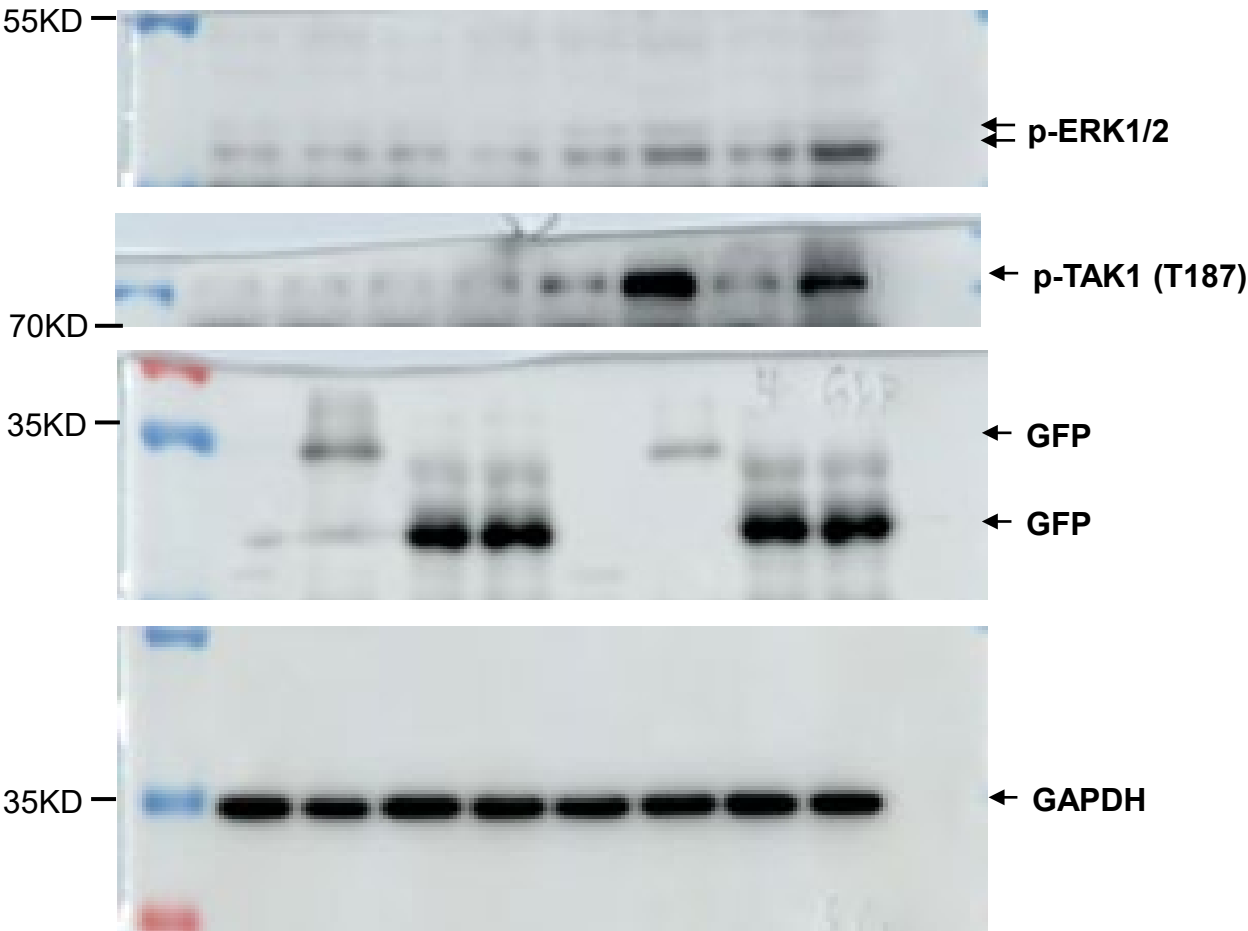

Supplement: Supplementary file 3 — Source Data for Expanded View [file EMBR-22-e51678-s001.zip › embr202051678-sup-0007-SDataEV/EV-Figure_Source_Data/Figure_EV5_Source_Data.pdf]

Figure 2

C

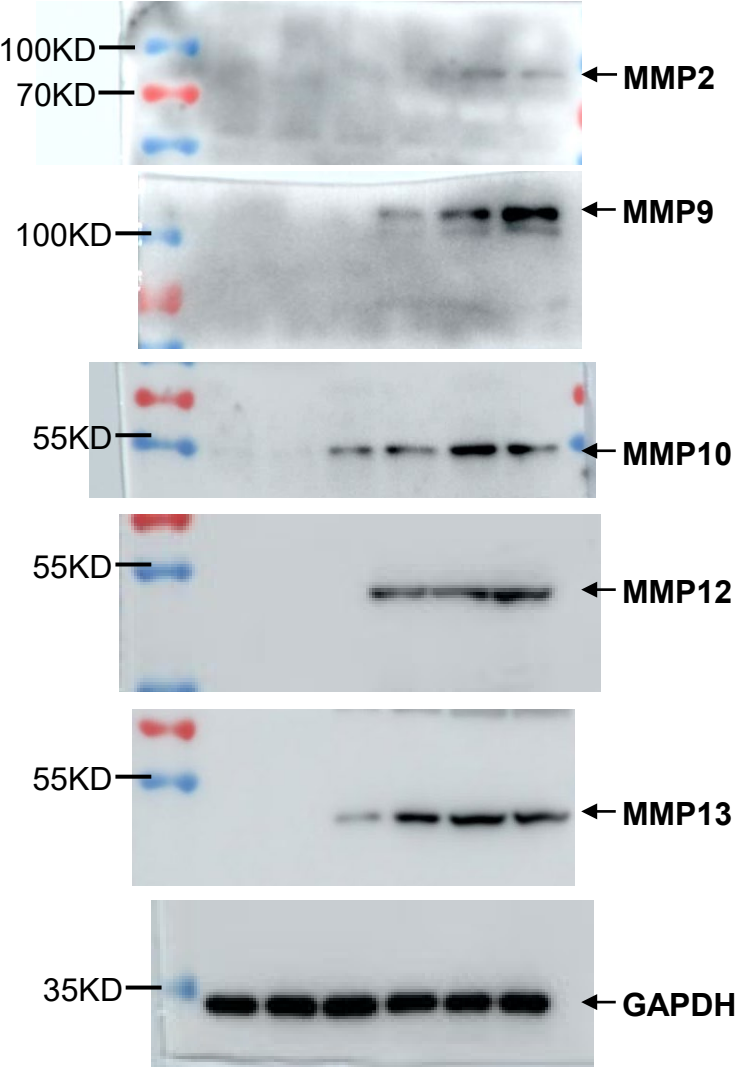

F

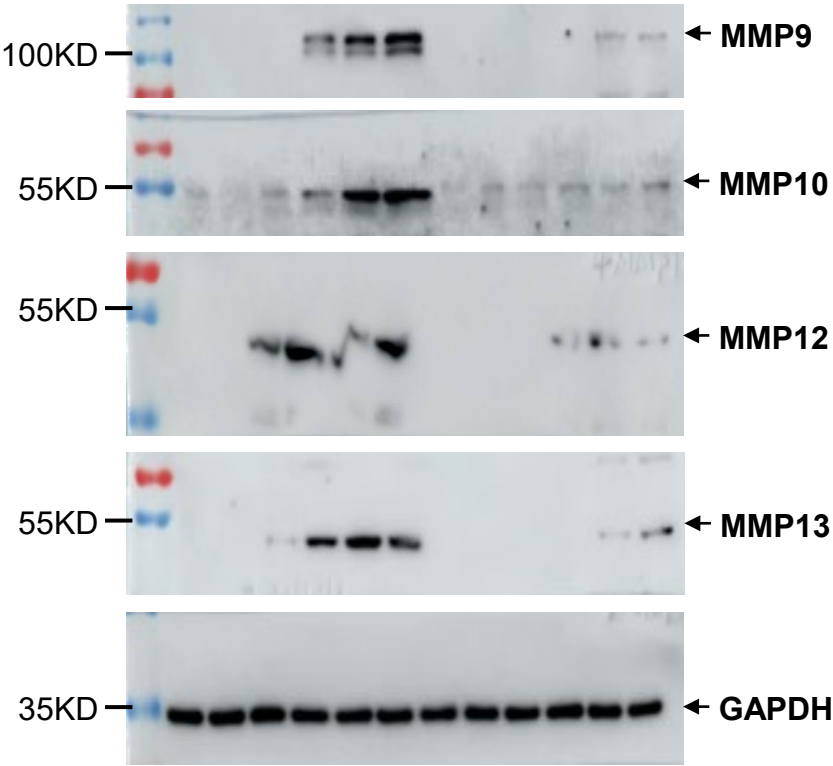

I

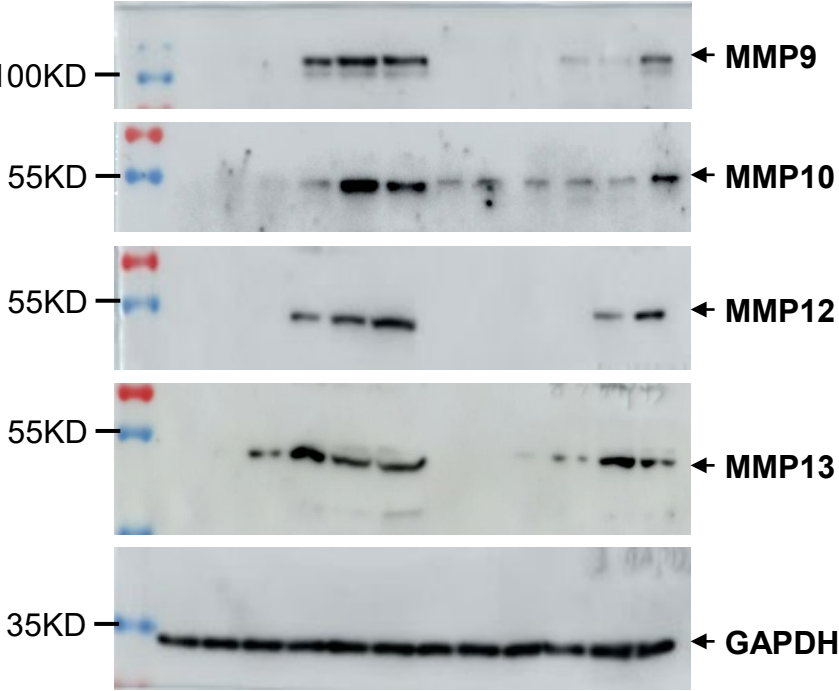

Supplement: Supplementary file 4 — Source Data for Figure 2 [file EMBR-22-e51678-s007.pdf]

Figure 3

**A**

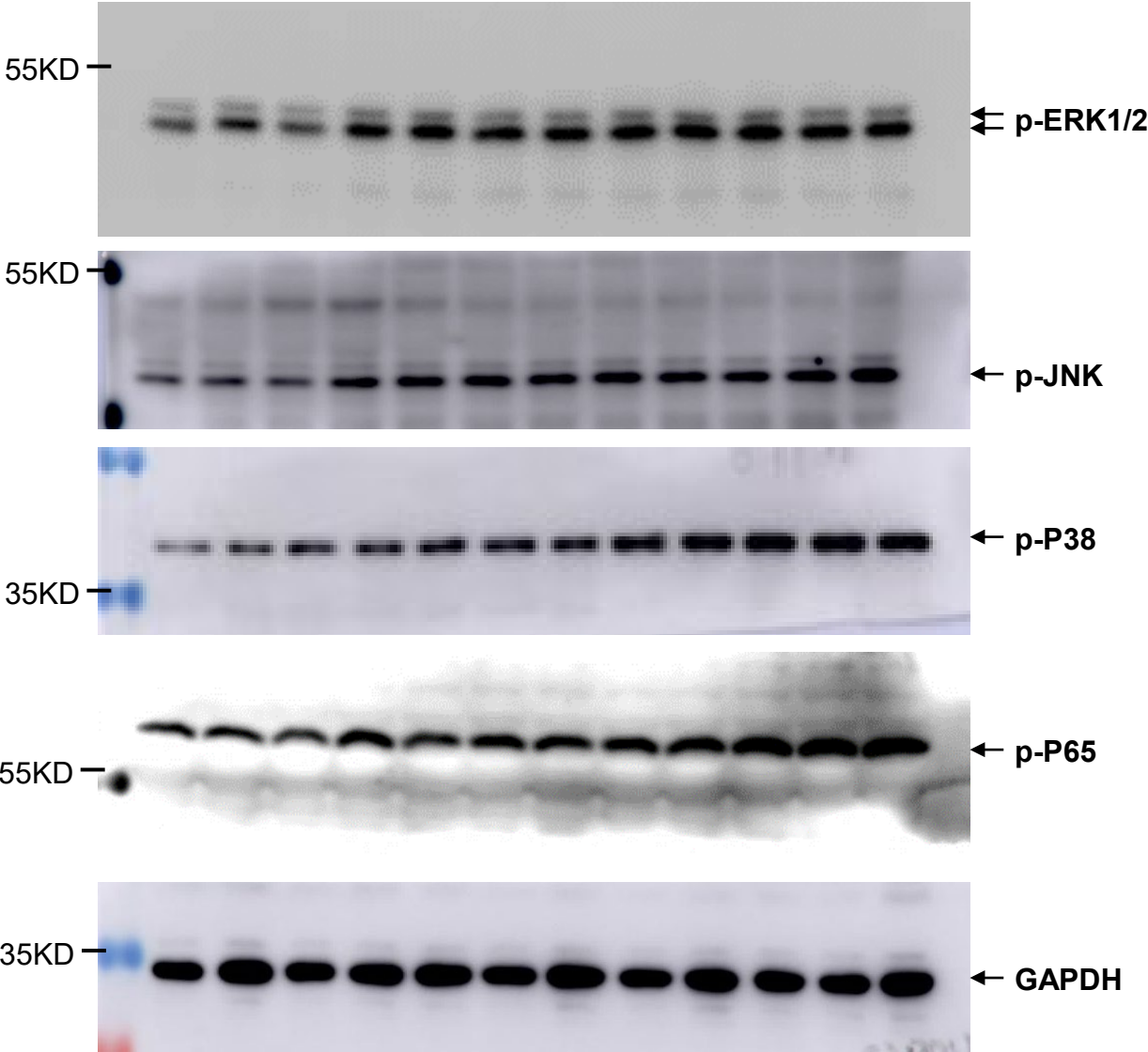

**B**

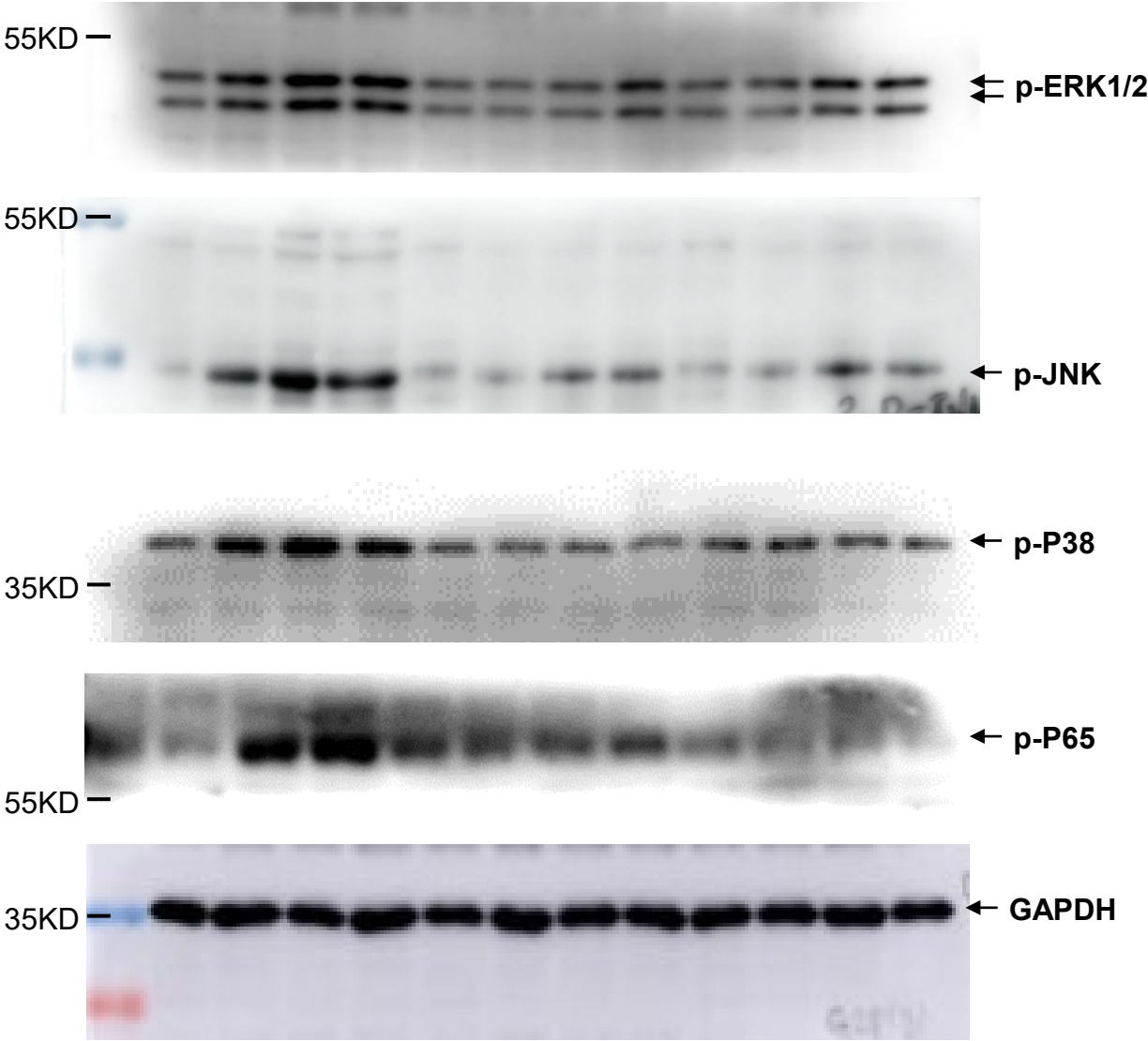

### Figure 3

**F**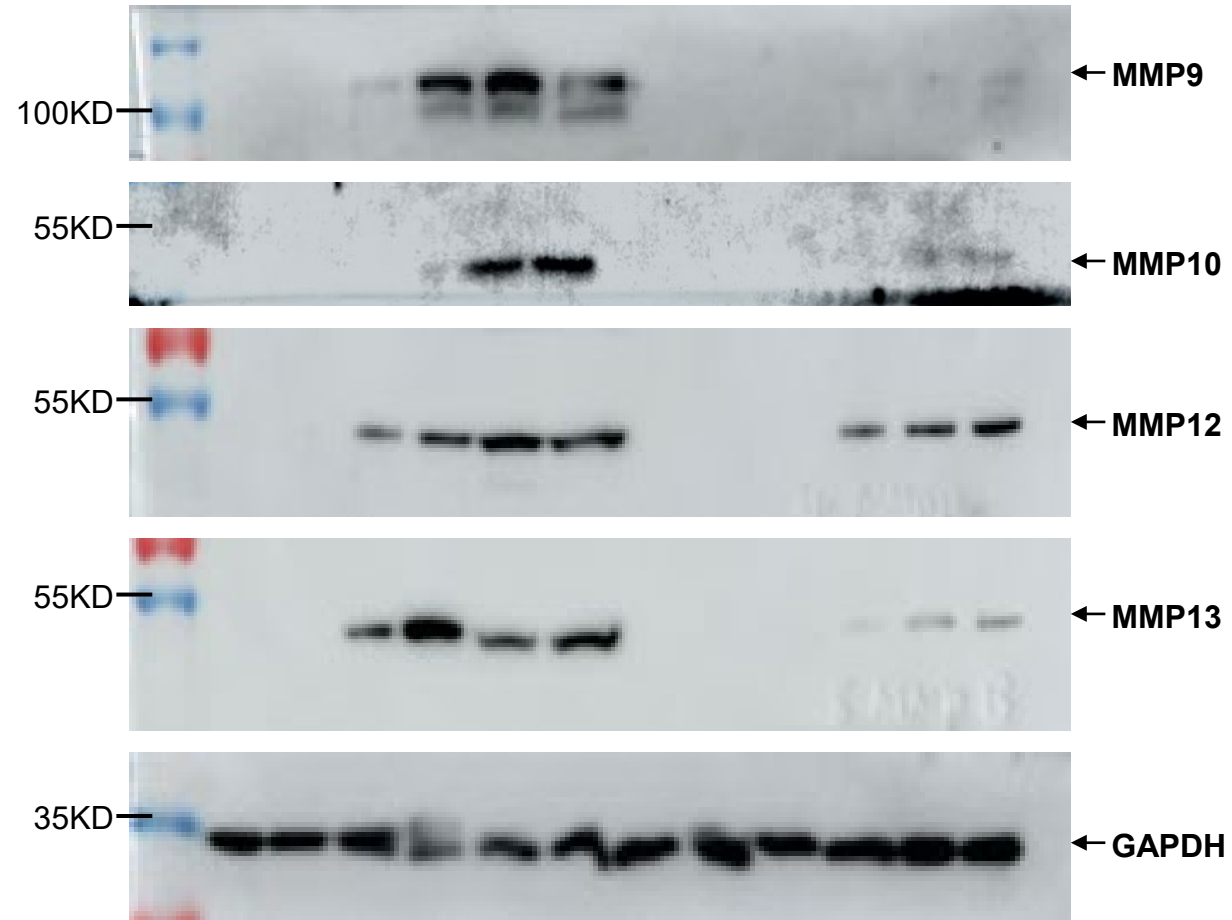

## G

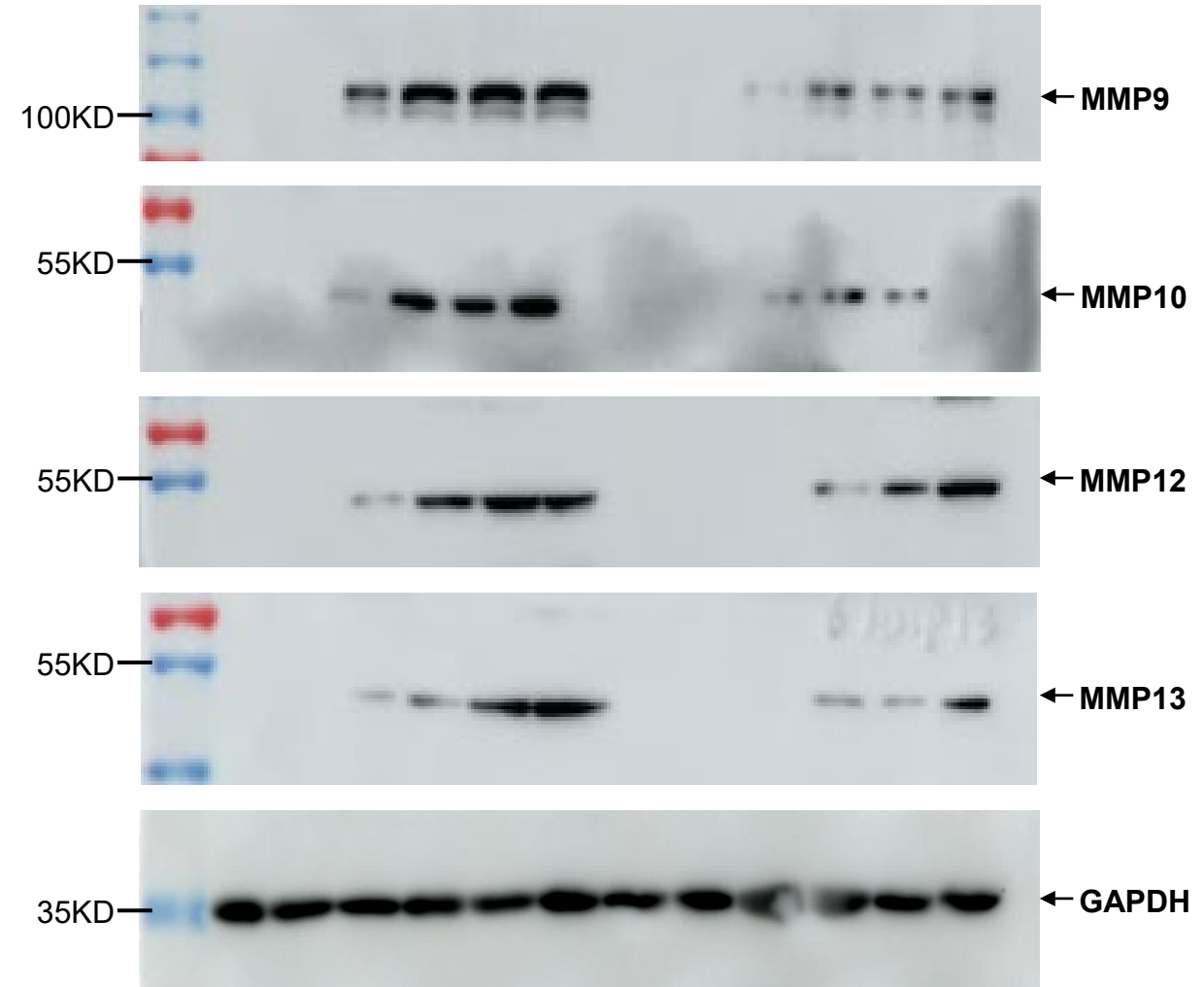

Supplement: Supplementary file 5 — Source Data for Figure 3 [file EMBR-22-e51678-s004.pdf]

Figure 6

**A**

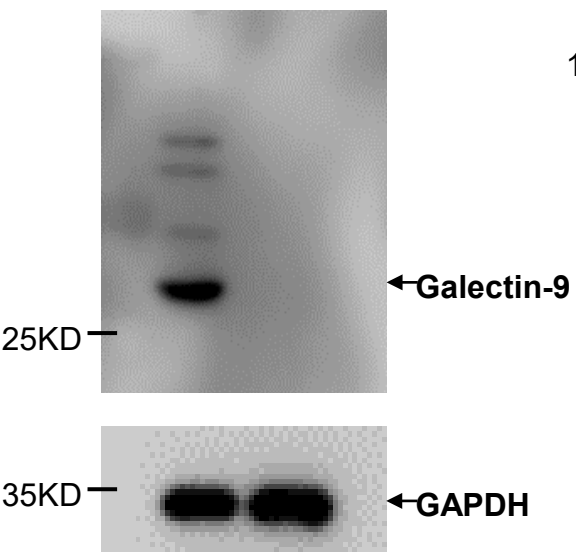

**F**

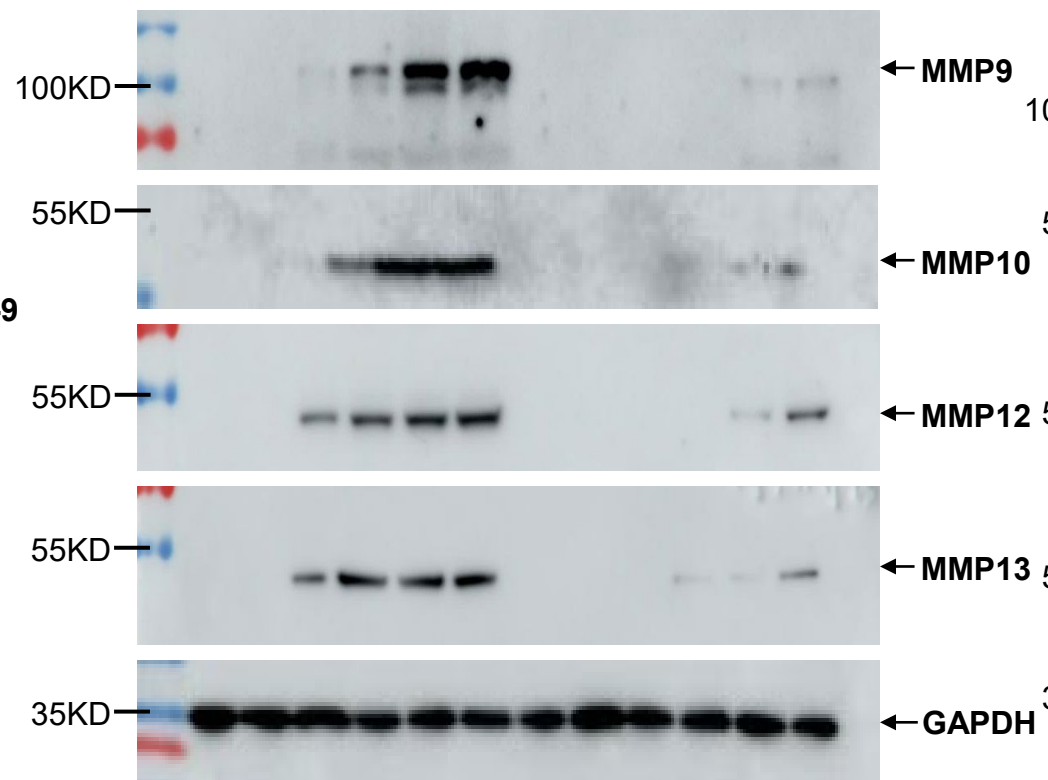

**G**

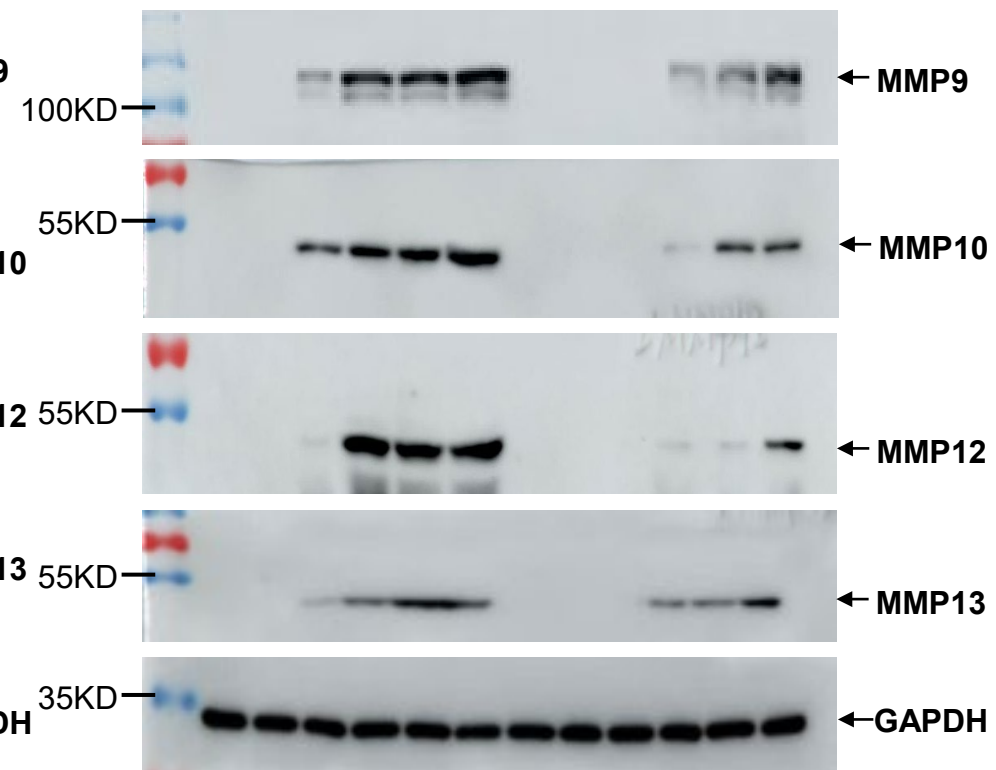

Figure 6

H

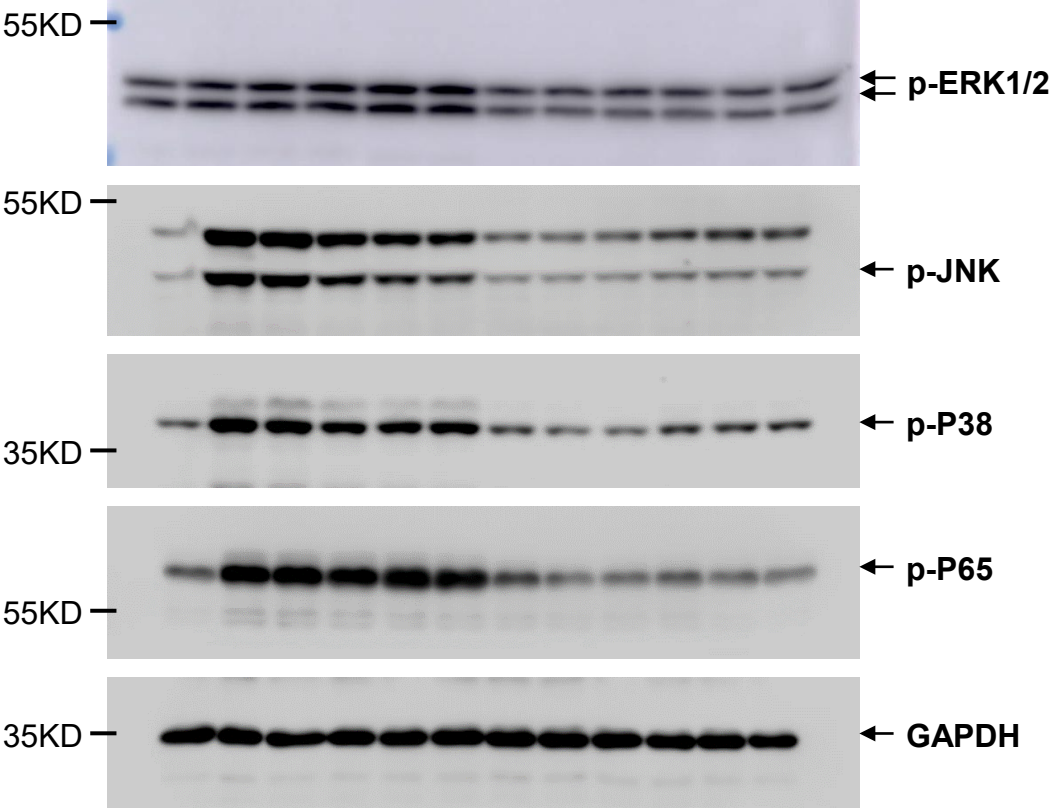

I

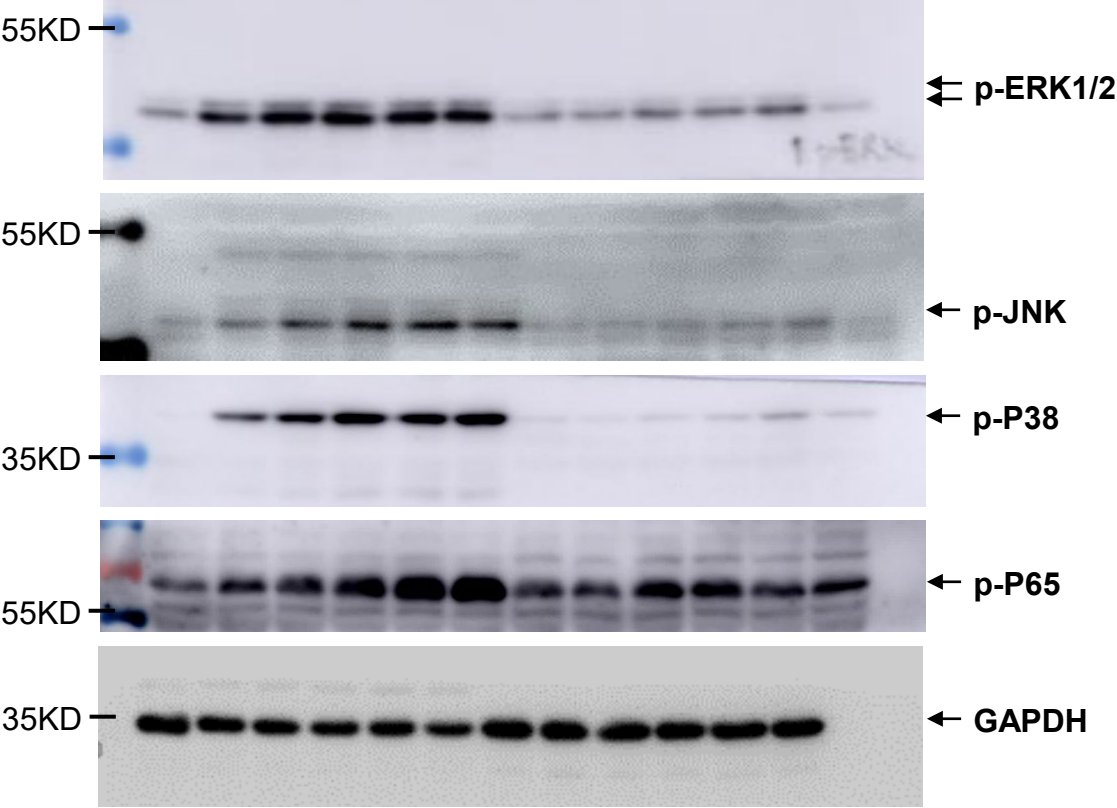

Supplement: Supplementary file 6 — Source Data for Figure 6 [file EMBR-22-e51678-s006.pdf]

Figure 7

A

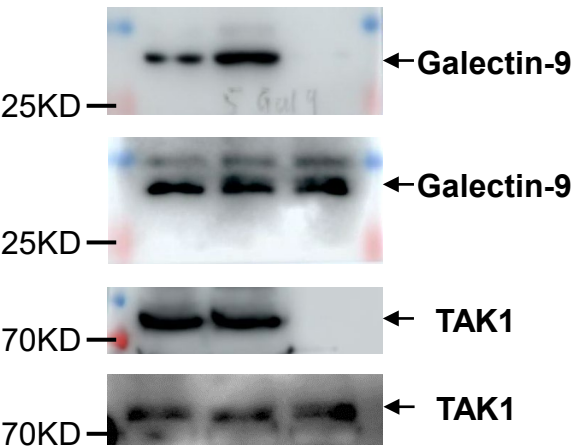

B

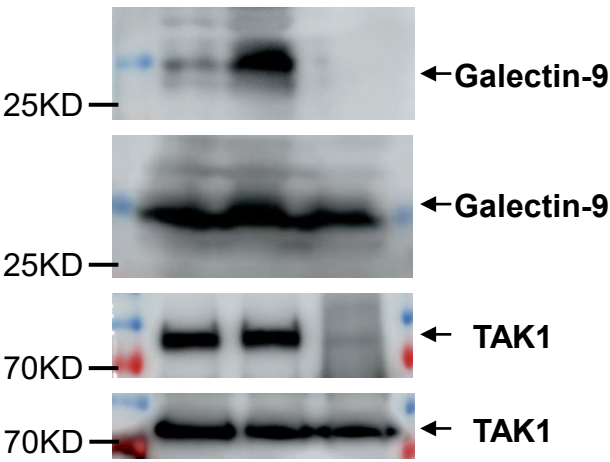

C

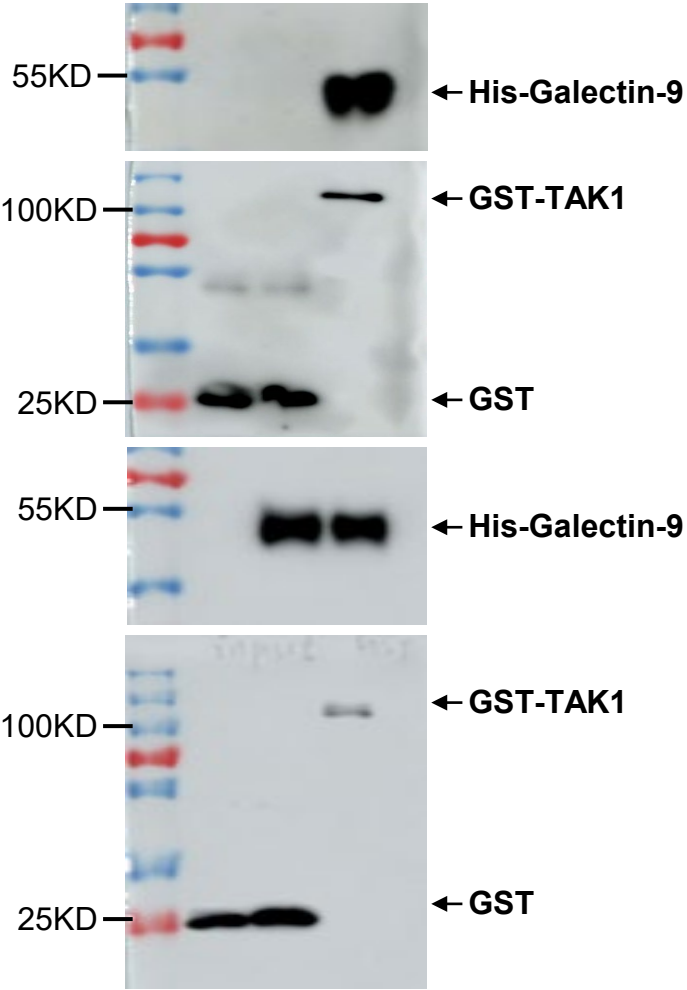

Figure 7

E

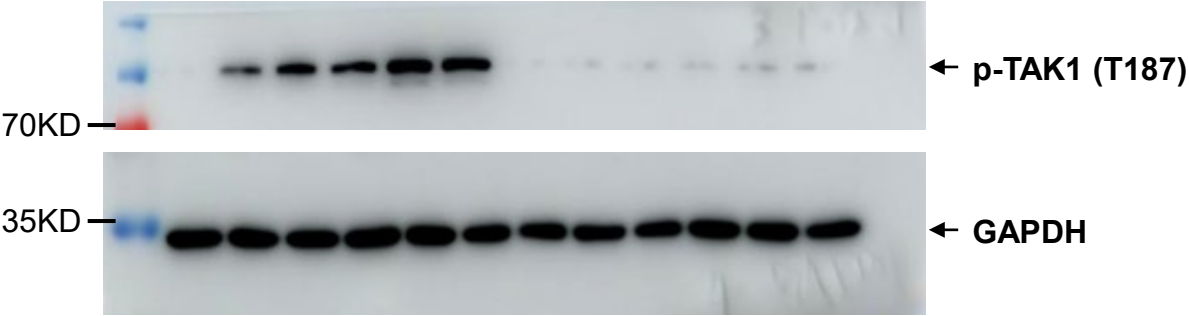

F

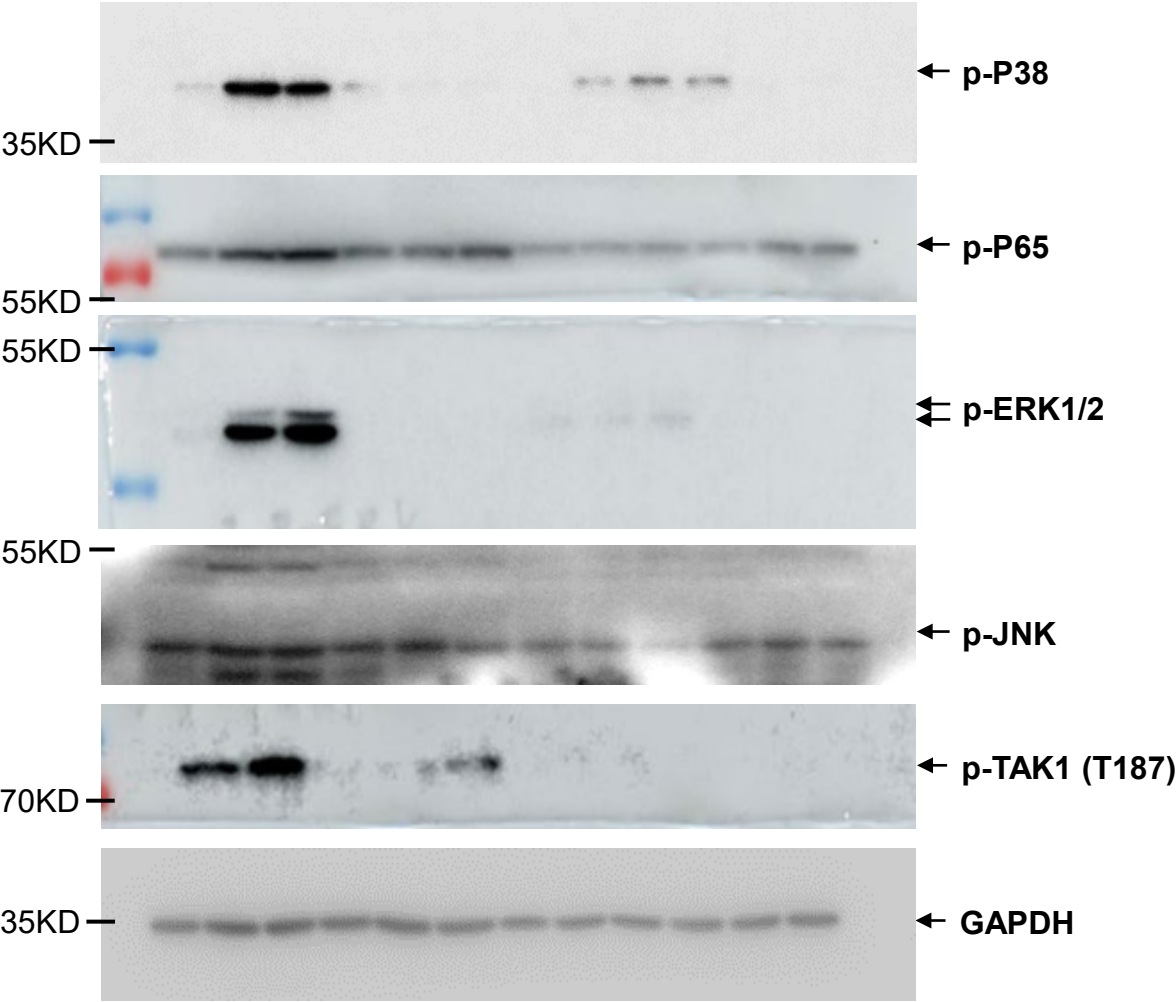

Supplement: Supplementary file 7 — Source Data for Figure 7 [file EMBR-22-e51678-s003.pdf]
