# Supplementary material for: Sensing of mycobacterial arabinogalactan by galectin‐9 exacerbates mycobacterial infection
Source: EMBO Rep. 2021 May 13;22(7):e51678. doi: 10.15252/embr.202051678 (PMC8256295; doi:10.15252/embr.202051678)
Supplement: Supplementary file 8 [file EMBR-22-e51678-s008.pdf]

EMBO reports

Sensing of arabinogalactan by galectin-9 exacerbates mycobacterial infection

Xiangyang Wu, Yong Wu, Ruijuan Zheng, Fen Tang, Lianhua Qin, Detian Lai, Lu Zhang, Lingming Chen, Bo Yan, Hua Yang, Yang Wang, Feifei Li, Jinyu Zhang, Fei Wang, Lin Wang, Yajuan Cao, Mingtong Ma, Zhonghua Liu, Jianxia Chen, Xiaochen Huang, Jie Wang, Ruiliang Jin, Peng Wang, Qin Sun, Wei Sha, Liangdong Lyu, Pedro Moura-Alves, Anca Dorhoi, Gang Pei, Peng Zhang, Jiayu Chen, Shaorong Gao, Felix Randow, Gucheng Zeng, Chang Chen, Xin-shan Ye, Stefan Kaufmann, Haipeng Liu, and Baoxue Ge
DOI: 10.15252/embr.202051678

Corresponding author(s): Haipeng Liu (haipengliu@tongji.edu.cn) , Stefan Kaufmann (kaufmann@mpiib-berlin.mpg.de), Haipeng Liu (haipengliu@tongji.edu.cn), Baoxue Ge (baoxue_ge@tongji.edu.cn), Xin-shan Ye (xinshan@bjmu.edu.cn)

Review Timeline:

Submission Date:	5th Sep 20
Editorial Decision:	15th Oct 20
Revision Received:	15th Feb 21
Editorial Decision:	24th Mar 21
Revision Received:	10th Apr 21
Accepted:	16th Apr 21

Editor: Achim Breiling

Transaction Report:

(Note: With the exception of the correction of typographical or spelling errors that could be a source of ambiguity, letters and reports are not edited. Depending on transfer agreements, referee reports obtained elsewhere may or may not be included in this compilation. Referee reports are anonymous unless the Referee chooses to sign their reports.)

1st Editorial Decision **15th Oct 2020**

Dear Dr. Liu,

Thank you for the submission of your research manuscript to EMBO reports. We have now received reports from the three referees that were asked to evaluate your study, which can be found at the end of this email.

As you will see, all referees think that the findings are of interest, but they also have several comments, concerns and suggestions, indicating that a major revision of the manuscript is necessary to allow publication in EMBO reports. As the reports are below, and I think all points need to be addressed, I will not detail them here.

Given the constructive referee comments, we would like to invite you to revise your manuscript with the understanding that all referee concerns must be addressed in the revised manuscript and in a detailed point-by-point response. Acceptance of your manuscript will depend on a positive outcome of a second round of review. It is EMBO reports policy to allow a single round of revision only and acceptance of the manuscript will therefore depend on the completeness of your responses included in the next, final version of the manuscript.

Revised manuscripts should be submitted within three months of a request for revision. We are aware that many laboratories cannot function at full efficiency during the current COVID-19/SARS-CoV-2 pandemic and we have therefore extended our 'scooping protection policy' to cover the period required for full revision. Please contact me to discuss the revision should you need additional time, and also if you see a paper with related content published elsewhere.

When submitting your revised manuscript, please also carefully review the instructions that follow below.

PLEASE NOTE THAT upon resubmission revised manuscripts are subjected to an initial quality control prior to exposition to re-review. Upon failure in the initial quality control, the manuscripts are sent back to the authors, which may lead to delays. Frequent reasons for such a failure are the lack of the data availability section (please see below) and the presence of statistics based on n=2 (the authors are then asked to present scatter plots or provide more data points).

When submitting your revised manuscript, we will require:

1) a .docx formatted version of the final manuscript text (including legends for main figures, EV figures and tables), but without the figures included. Please make sure that changes are highlighted to be clearly visible. Figure legends should be compiled at the end of the manuscript text.

2) individual production quality figure files as .eps, .tif, .jpg (one file per figure), of main figures and EV figures. Please upload these as separate, individual files upon re-submission.

The Expanded View format, which will be displayed in the main HTML of the paper in a collapsible format, has replaced the Supplementary information. You can submit up to 5 images as Expanded View. Please follow the nomenclature Figure EV1, Figure EV2 etc. The figure legend for these should be included in the main manuscript document file in a section called Expanded View Figure Legends after the main Figure Legends section. Additional Supplementary material should be supplied as a single pdf file labeled Appendix. The Appendix should have page numbers and needs

to include a table of content on the first page (with page numbers) and legends for all content. Please follow the nomenclature Appendix Figure Sx, Appendix Table Sx etc. throughout the text, and also label the figures and tables according to this nomenclature.

For more details please refer to our guide to authors:
http://www.embopress.org/page/journal/14693178/authorguide#manuscriptpreparation

See also our guide for figure preparation:
http://wol-prod-cdn.literatumonline.com/pb-assets/embo-site/EMBOPress_Figure_Guidelines_061115-1561436025777.pdf

3) a .docx formatted letter INCLUDING the reviewers' reports and your detailed point-by-point responses to their comments. As part of the EMBO Press transparent editorial process, the point-by-point response is part of the Review Process File (RPF), which will be published alongside your paper.

4) a complete author checklist, which you can download from our author guidelines (https://www.embopress.org/page/journal/14693178/authorguide). Please insert page numbers in the checklist to indicate where the requested information can be found in the manuscript. The completed author checklist will also be part of the RPF.

Please also follow our guidelines for the use of living organisms, and the respective reporting guidelines: http://www.embopress.org/page/journal/14693178/authorguide#livingorganisms

5) that primary datasets produced in this study (e.g. RNA-seq, ChIP-seq and array data) are deposited in an appropriate public database. This is now mandatory. If no primary datasets have been deposited in any database, please also state this (e.g. 'No primary datasets have been generated and deposited') in this section (see below).

See also: http://embor.embopress.org/authorguide#datadeposition

Please remember to provide a reviewer password if the datasets are not yet public.

The accession numbers and database should be listed in a formal "Data Availability " section (placed after Materials & Methods) that follows the model below. Please note that the Data Availability Section is restricted to new primary data that are part of this study.

Data availability

The datasets produced in this study are available in the following databases:

- RNA-Seq data: Gene Expression Omnibus GSE46843 (https://www.ncbi.nlm.nih.gov/geo/query/acc.cgi?acc=GSE46843)
- [data type]: [name of the resource] [accession number/identifier/doi] ([URL or identifiers.org/DATABASE:ACCESSION])

*** Note - All links should resolve to a page where the data can be accessed. ***

Moreover, I have these editorial requests:

6) We strongly encourage the publication of original source data with the aim of making primary data more accessible and transparent to the reader. The source data will be published in a separate source data file online along with the accepted manuscript and will be linked to the relevant figure. If you would like to use this opportunity, please submit the source data (for example scans of entire gels or blots, data points of graphs in an excel sheet, additional images, etc.) of your key experiments together with the revised manuscript. If you want to provide source data, please include size markers for scans of entire gels, label the scans with figure and panel number, and send one PDF file per figure.

7) Our journal encourages inclusion of *data citations in the reference list* to directly cite datasets that were re-used and obtained from public databases. Data citations in the article text are distinct from normal bibliographical citations and should directly link to the database records from which the data can be accessed. In the main text, data citations are formatted as follows: "Data ref: Smith et al, 2001" or "Data ref: NCBI Sequence Read Archive PRJNA342805, 2017". In the Reference list, data citations must be labeled with "[DATASET]". A data reference must provide the database name, accession number/identifiers and a resolvable link to the landing page from which the data can be accessed at the end of the reference. Further instructions are available at:
http://www.embopress.org/page/journal/14693178/authorguide#referencesformat

8) Regarding data quantification and statistics, can you please specify, where applicable, the number "n" for how many independent experiments (biological replicates) were performed, the bars and error bars (e.g. SEM, SD) and the test used to calculate p-values in the respective figure legends. Please provide statistical testing where applicable, and also add a paragraph detailing this to the methods section. See:
http://www.embopress.org/page/journal/14693178/authorguide#statisticalanalysis

9) Please also note our new reference format:
http://www.embopress.org/page/journal/14693178/authorguide#referencesformat

10) Please add up to five key words to the title page of the manuscript.

I look forward to seeing a revised version of your manuscript when it is ready. Please let me know if you have questions or comments regarding the revision.

Yours sincerely,

Achim Breiling
Editor
EMBO Reports

Referee #1:

The manuscript by Wu et al. demonstrates that mycobacterial arabinogalactan (AG) is a virulence factor in mycobacteria and is recognized by galectin-9. This interaction leads to the activation of the TAK1-ERK pathway, ultimately leading to the induction of the expression of matrix metalloproteinases (MMPs), which participate in the destruction of lung tissues during active tuberculosis.

The overall study is of high biological significance as it identifies a new biological function of AG in

the pathogenesis of tuberculosis, acting as a PAMP. It also convincingly demonstrates, through a wide panel of techniques (siRNA, galectin KO mice, specific inhibitors...), that galactine-9 acts at the pattern recognition receptor of AG. As such, the study is not only of interest for the mycobacterial community but also for a broader readership interested in host-pathogen interactions in general and in signaling pathways leading to pathology in infectious diseases. It also opens the way for future developments of a novel type of host-directed therapy (as elegantly demonstrated through the use of AG aptamers) targeting galactin-9 in adjunct to classical chemotherapy in TB.

In general, the manuscript is well-written and experiments well conducted. The conclusions raised are supported by compelling data and robustly documented using independent lines of experimental evidence. The manuscript appears particularly suited for a long-format article.

However, the impact of the study may be improved and additional controls/epxeriments are required prior to publication:
1.Very little information is given regarding how the 3 aptamers were selected. While AA932 clearly showed reduced inflammation in the lungs in mice and increased survival in TB-infected mice, the corresponding data with AA618 and AA835 are missing. Perhaps it would be interesting to show in the supplementary files the effect of these aptamers in reducing the lung pathology in mice.

2.The authors convincingly demonstrate that AG significantly increases gene expression of various MMPs in mouse peritoneal macrophages, human THP1 cells and lungs. However, since all the conclusions were based on qRT-PCR analyses, it remains important to demonstrate that this translates also into increased expression of the MMP proteins/activity. Western blotting using specific MMP antibodies and/or gelatin zymography may be helpful. This is particularly important to consider knowing that MMPs are secreted matrix-degrading enzymes.

3.Fig. 2A, presents only a restricted view of the genes up- or down-regulated by AG. It would be helpful for the reader to have access the complete transcriptome in AG-treated macrophages. One would anticipate that many more genes are regulated under these conditions. This would also help to understand why the authors focused specifically on the mmp genes.

4.Fig 2 B-G concentrate on MMP2, 9, 10, 12 and 13 while expression of mmp12 is not shown in Fig 2A. Perhaps Fig 2A could be completed.

5.Fig S2A. More details should be provided regarding the MMAR_5356 and 5357 genes in M. marinum. What are the functions of these genes in AG biosynthesis? If they play an important role in AG biosynthesis, and considering that AG is essential for mycobacterial growth, one would expect these knock-down mutants to be sick in the presence of tetracycline treatment, associated with important changes in the cell wall composition/architecture, which in turn may explain, at least partly, the reduced mmp expression in mouse peritoneal macrophages (Fig S2C). In other terms, the observed effect may not be a direct effect of AG down-regulation but rather deeper cell wall/growth defect. It thus appears necessary to provide the growth curves of the knock-down mutants.

6.Since AG is hidden and surrounded by the outermost mycomembrane layer in mycobacteria, it is hard to understand how the galactan chain of AG can be exposed the mycobacterial cell surface and can interact with galactan-9 (Fig. 5E-F). This should be better discussed in the Discussion section. In addition, the authors should also consider testing the binding of Galectin-9 to the MMAR_5356 and 5357 knock-down mutants.

7.Why did the authors infect the zebrafish embryos in the Duct of Cuvier? This is a very unusual

route of infection in zebrafish. They should consider injecting in the caudal vein (100 CFU) for systemic infection. Were the aptamers maintain in fish water during the whole experiment (14 days ?). Did the authors check for toxicity of the aptamers in zebrafish ? This is an important control to show.

Referee #2:

The manuscript entitled "Sensing of Arabinogalactan by galectin exacerbates mycobacterial infection through inducing matrix metalloproteinases " describes that Arabinogalactan (AG) in mycobacterial cell wall qualifies for being called as pathogen associated molecular pattern (PAMP) which is sensed by pathogen recognition receptor (PRR) galectin-9 and aggravates mycobacterial infection by inducing membrane metallic proteinases. The authors report that AG induces Galectin-9 association with TAK1 and subsequent activation of MAPKs as well as MMP production.

1) Galectin-9 is a soluble secreted molecule - and not a transmembrane protein as shown the model by the authors. A signaling domain has not been described. Galectin-3 primarily acts as a cytosolic sensor. How to the authors propose that galectin-9 leads to the activation of TAK1 pathway?
2) When Mtb infects, Gal9 could possibly bind to Mtb, As such, there might be Gal9 bound to Mtb in the phagosome. How then, does Gal9 signal via TAK1, which is in the cytosol?
3) Is there any evidence that Mtb infection of macrophages stimulates TAK1?
4) As galectin-9 binds carbohydrates, it's binding it promiscuous and there are many different proteins it can bind and has important interactions with host proteins. The finding that a Gal9 ko mouse differs in disease following infection or treatment with AG does not lead to the conclusion that the disease requires AG/Gal9 interaction. Certainly, macrophage infection and intracellular growth is normal in the absence of galectin-9 as has been previously published.
5) The finding described in the manuscript are not novel in terms of AG sensing by Galectins. The studies by Erminia Barboni et al in the manuscript "The binding of mycolic acids to galectin-3: A novel interaction between a host soluble lectin and trafficking mycobacterial lipids? has already shown direct interaction of mycolic acids (that contains and is bound to AG) with galectin-3. The authors should cite and discuss this paper.
6) The term AG as a PAMP is a misnomer. As per the results in the manuscript intraperitoneal or intravenous administration of AG cause lung injury and induces MMPs. Moreover, AG-specific aptamers extended the survival of Mtb- infected SCID mice or M. marinum-infected zebrafish. The data presented demonstrate AG as virulence factor and not PAMP. There is no evidence of pro-inflammatory and protective immune response by host presented in the manuscript that makes AG qualifies for PAMP. The author mentions in line 331 to 334 in discussion AG is a virulence factor. It is confusing. AG seems to be a virulence factor deploying galectin-9 to establish infection by Mtb rather than providing anti-microbial immunity (refer reference 60).
7) The authors mentioned that treatment of AG leads to Galentin-9 upregulation also doesn't seems to convince the fact that Galectin-9 is a PRR and AG as PAMP. The Galectins expression fluctuates in many diseases and their expression is upregulated in many of diseases including cancer and bacterial infection.
8) The infection model used in this paper is inappropriate. This is a model of lung injury and not Mtb pathogenesis
9) The results presented in the manuscript does for the first time demonstrates the Galectin -9 - TAK1 pathway utilized by AG as a Mtb virulence factor that causes lung injury by inducing MMPs . Using a combination of SPR for demonstrating interaction of AG and Galectin-9 and immune-

precipitation of Galectin-9 and TAK1 demonstrates the pathway utilized.
10) Why in the galectin 9 ko is inflammation going down while CFU is going up?
Minor points:
11) The survival analysis in Fig.1H, I is incorrect (the statistical test is overaggressive and clearly there is not survival advantage). There is clearly no different in MST.
12) In figure 3A, a lot of heterogeneity is seen in the GAPDH controls. For eg. comparing pERK in 0 time point versus 5 there is an increase in GAPDH and so is pERK, while in 0 and 15 the GAPDH seems to be equal and thus the pERK levels doesn't increase. Same is the case in time point 120, 240, and 600. A normalized quantitative bargraph of pERK with GAPDH alongside would be more convincing for the results presented. It could be possible that pERK levels do increase.
13) In Fig.5 E/F, why is only a small portion of the molecules binding
14) Fig 6F likely have labelling mistake, YFP-galectin 9 confused with GFP and IP and IB labels in the subsequent blots.
15) the authors don't mention the immunoprecipitation protocol in material and methods and its difficult to figure out the experiment for the reader. It needs to be corrected.
16) The high dose IN Mtb infection model should be abandoned. With the pivot towards lower dose infection, it is unlikely to be telling us anything about the host-pathogen response.

Referee #3:

Major comments:
(1) The authors state that galectin-9 shows specific interaction with AG based by SPR assays (Figure 5 and EV3). However, this reviewer raised serious concerns about the quality of biochemical analyses. First, the sensorgram of galectin-9 and AG (Fig. 5A) shows very weak signals compared with the others (Fig. EV3). In addition, the fitting curves are significantly deviated from actual signals. Furthermore, the chi2 values of fitting curves should be clearly shown. Overall, this reviewer recommend the re-examination of these series of experiments under the apporpriate conditions. Second, the authors expressed CRD1 and 2 by using bacterial expression systems. Although the N-terminus of CRD1 starts at 16, the beta-sandwich fold of CRD1 originally starts at Ser6 in crystal structure. Weak sugar affinity of CRD1 might be derived from misfolding due to invalid construct design and it would not be appropriate to compare binding affinity using such construc. Third, the authors showed the purity of CRD1 and 2 in Fig. 5B. However, there is no description regarding the purity of recombinant full-length galectin-1~14. The authors should show the purity of these commercial proteins as well.
(2) Considering well-studied galectin-9 structure and its galectin-binding mode, it is difficult to imagine that Galectin-9 can bind garactofranoside. Galectins specifically recognize beta-galactoside via highly conserved sugar binding sites. Within this binding sites, the OH4 and OH6 of beta-galactoside tightly interact with galectins. The authors described that galectin-9 binds to AG via beta-galactofuranose, though it lacks both OH4 and OH6 at the equivalent positions. The authors would be better to show feasible explanation with experimental data together with structural insight.
(3) The authors did not address the localization of this protein. How does the Galectin-9 bind to AG outside of the cells and thereafter associate with TAK in the cytoplasm. The author should provide reasonable experimental evidence to support the authors' conclusion in terms of protein trafficking.

Minor comments:
(1) References were not appropriately cited.
(2) The authors interpreted that the administration of aptamer against AG prolonged the survival

upon Mtb infection. However, the merginal difference would be difficult to prove the authors' conclusion.
(3) page 11, line 224, the authors' description "Therefore, our results suggest that galectin-9 acts as a specific PRR for AG." is overstated.
(4) It would be better to show reliable data that demonstrate the feasible mode of the interaction of Galectin-9 and TAK.

Point to point responses

We are very grateful for the reviewers' constructive comments. We have revised the manuscript thoroughly (highlighted in blue) and hope to address each of the concerns from the reviewers.

Referee #1:
The manuscript by Wu et al. demonstrates that mycobacterial arabinogalactan (AG) is a virulence factor in mycobacteria and is recognized by galectin-9. This interaction leads to the activation of the TAK1-ERK pathway, ultimately leading to the induction of the expression of matrix metalloproteinases (MMPs), which participate in the destruction of lung tissues during active tuberculosis.

The overall study is of high biological significance as it identifies a new biological function of AG in the pathogenesis of tuberculosis, acting as a PAMP. It also convincingly demonstrates, through a wide panel of techniques (siRNA, galectin KO mice, specific inhibitors...), that galactine-9 acts at the pattern recognition receptor of AG. As such, the study is not only of interest for the mycobacterial community but also for a broader readership interested in host-pathogen interactions in general and in signaling pathways leading to pathology in infectious diseases. It also opens the way for future developments of a novel type of host-directed therapy (as elegantly demonstrated through the use of AG aptamers) targeting galactin-9 in adjunct to classical chemotherapy in TB.

In general, the manuscript is well-written and experiments well conducted. The conclusions raised are supported by compelling data and robustly documented using independent lines of experimental evidence. The manuscript appears particularly suited for a long-format article.
We thank the reviewer for the encouraging comments on our work.

However, the impact of the study may be improved and additional controls/experiments are required prior to publication:

1.Very little information is given regarding how the 3 aptamers were selected. While AA932 clearly showed reduced inflammation in the lungs in mice and increased survival in TB-infected mice, the corresponding data with AA618 and AA835 are missing. Perhaps it would be interesting to show in the supplementary files the effect of these aptamers in reducing the lung pathology in mice.
We appreciate the reviewer's concern. In this study, we obtained aptamers against chemically synthesized mycobacterial AG by systematic evolution of ligands by exponential enrichment (SELEX) process (Bock, Griffin et al., 1992, Tuerk & Gold, 1990), which has been previously successfully applied for the screening of aptamers targeting *Mtb* (Aimaiti, Qin et al., 2015, Golichenari, Nosrati et al., 2018, Qin, Liu et al., 2014, Sun, Pan et al., 2016, Tang, Wu et al., 2016, Zhang, Feng et al., 2017).

Moreover, we used enzyme-linked immunosorbent assay (ELISA) to determine their respective affinities against AG. Using enzyme-linked plates, we systematically monitored the selection process to obtain additional aptamers against AG and to better understand the dynamic evolution of aptamers against AG. After nine rounds of selection the maximum level of affinity was obtained and three aptamers (AA618, AA835, AA932) of different structures with high affinity were selected. Among them, the frequency of aptamer AA932 sequence was highest, which was considered as the preponderant aptamer **(line 159-line 171).**

Moreover, by performing a functional assay, we observed that both AA932 and AA835 showed inhibitory effects on the induction of MMPs in response to AG stimulation **(Fig 2E)**, while AA618 had no inhibitory effects on the induction of MMPs **(data not shown)**. According to our data, AA932 potently inhibits AG-induced MMPs, and consistently slightly better than AA835 (**Fig 2E**), indicating that AA932 is best-suited targeting AG-galectin-9 interface. We, therefore, performed the animal experiments with AA932 only rather than other aptamers **(line 199-line 203)**.

2.The authors convincingly demonstrate that AG significantly increases gene expression of various MMPs in mouse peritoneal macrophages, human THP1 cells and lungs. However, since all the conclusions were based on qRT-PCR analyses, it remains important to demonstrate that this translates also into increased expression of the MMP proteins/activity. Western blotting using specific MMP antibodies and/or gelatin zymography may be helpful. This is particularly important to consider knowing that MMPs are secreted matrix-degrading enzymes.

We appreciate the reviewer's comments. As per the reviewer's suggestion, we have performed additional experiments to detect MMPs at protein level using specific MMP antibodies by western blotting. The data demonstrated that AG stimulation increased the secretion of MMPs **(new Fig 2C; line 196-line 197)** and that AG aptamers (e.g. AA932) inhibited the secretion of MMPs protein, both in response to AG stimulation **(new Fig 2F; line 202-line 203)** or *Mtb* infection **(new Fig 2I; line 219-line 220)**. The secretion of MMPs proteins in response to AG stimulation or *Mtb* infection was markedly impaired when cells were treated with ERK or TAK1 inhibitors **(Fig 3F and G, Fig EV5C and D; line 234-line 239)**. Moreover, the deletion of galectin-9 markedly impacted the secretion of MMPs by macrophages upon stimulation with AG or infection with *Mtb* **(Fig 6F and G; line 296-line 300)**. These data further consolidate our previous findings that engagement of galectin-9 activates the TAK1-ERK-MMPs signaling axis. We have included the new data in the revised manuscript.

3.Fig. 2A, presents only a restricted view of the genes up- or down-regulated by AG. It would be helpful for the reader to have access the complete transcriptome in AG-treated macrophages. One would anticipate that many more genes are regulated under these conditions. This would also help to understand why the authors focused specifically on the mmp genes.

We appreciate the reviewer's concern. As per the reviewer's suggestion, we provide the information of complete transcriptome in AG-treated macrophages in the revised manuscript. The RNA-Seq data from this publication have been deposited to the GEO database [https://www.ncbi.nlm.nih.gov/geo/query/acc.cgi?acc=GSE166850] and assigned the identifier [GSE166850]). GO pathway analysis revealed an enrichment of multiple MMPs **(new Fig EV1A-C)**. Considering the important role of MMPs in the pathogenesis of TB (Kathamuthu, Kumar et al., 2020, Ong, Elkington et al., 2014, Parasa, Muvva et al., 2017, Sabir, Hussain et al., 2019, Volkman, Pozos et al., 2010), we decided to specifically focus on these genes in our study. The function and mechanisms of AG-induced genes other than MMPs expression warrant further investigation in the future **(line 187-line 193)**.

4.Fig 2 B-G concentrate on MMP2, 9, 10, 12 and 13 while expression of mmp12 is not shown in Fig 2A. Perhaps Fig 2A could be completed.
We appreciate the reviewer's concern. RNA-seq analysis did not reveal the upregulation of mmp12 expression in response to AG stimulation. However, quantitative RT-PCR measurement of the transcripts of a panel of mmps demonstrated AG induced the transcription of mmp12.

5. Fig S2A. More details should be provided regarding the MMAR_5356 and 5357 genes in M. marinum. What are the functions of these genes in AG biosynthesis? If they play an important role in AG biosynthesis, and considering that AG is essential for mycobacterial growth, one would expect these knock-down mutants to be sick in the presence of tetracycline treatment, associated with important changes in the cell wall composition/architecture, which in turn may explain, at least partly, the reduced mmp expression in mouse peritoneal macrophages (Fig S2C). In other terms, the observed effect may not be a direct effect of AG down-regulation but rather deeper cell wall/growth defect. It thus appears necessary to provide the growth curves of the knock-down mutants.
We appreciate the reviewer's concern. MMAR_5356 and 5357 genes in *M. marinum* are homologs of embA and embB in *Mycobacterium tuberculosis*, respectively, which are important for the biosynthesis of AG (Alderwick, Harrison et al., 2015, Dulberger, Rubin et al., 2020, Jankute, Cox et al., 2015, Zhang, Zhao et al., 2020). To further clarify whether these mutants regulate *Mmps* expression by affecting bacterial growth, we have performed extra experiments to measure the growth curves of the wild type and knock-down mutants of *M. marinum*. The data demonstrate that tetracycline-mediated knockdown of these genes did not significantly affect the growth of *M. marinum* in 7H10 culture medium **(new Appendix Fig S3D-F)**. Therefore, the AG down-regulation in the presence of tetracycline treatment is likely responsible for the reduced *Mmp*s expression in mouse peritoneal macrophages **(Appendix Fig S3C) (line 208-line 218)**.

6.Since AG is hidden and surrounded by the outermost mycomembrane layer in mycobacteria, it is hard to understand how the galactan chain of AG can be

exposed the mycobacterial cell surface and can interact with galactan-9 (Fig. 5E-F). This should be better discussed in the Discussion section. In addition, the authors should also consider testing the binding of Galectin-9 to the MMAR_5356 and 5357 knock-down mutants.

We appreciate the reviewer's concern. As the reviewer pointed out, *Mtb* possesses an unusual cell wall: the inner layer is peptidoglycan, the middle layer is highly branched AG and the outer layer is long-chain mycolic acid(Jankute et al., 2015) Both peptidoglycan and mycolic acid are known pathogen-associated molecular patterns (PAMPs) which trigger a cascade of innate immune responses(Bastos, Wheeler et al., 2021, Korf, Stoltz et al., 2005, Lu, Zheng et al., 2019, Tahiri, Fodran et al., 2020, Verschoor, Baird et al., 2012, Wolf, Reyes et al., 2016, Wolf & Underhill, 2018). In **Fig. 5E-F (revised Fig 5D and E)**, to clarify whether galectin-9 binds to mycobacteria directly, we harvested mycobacteria from a log phase culture in the presence of Tween 80 and incubated these bacteria with galectins followed by FACS analysis. The detergent in the culture may disrupt the out layer of the mycobacteria cell wall and expose AG which binds to galectin-9. Moreover, it has been reported that during replication, the bacteria expose newly synthesized AG (Meniche, Otten et al., 2014) which may in turn be recognized by galectin-9 **(line 458-line 481)**.

We thank the reviewer for the insightful suggestion. We analyzed the binding of galectin-9 to wild type and *MMAR_5356* or *MMAR_5357* knock-down mutants of *M. marinum*. The data demonstrate that tetracycline-mediated knockdown of both genes reduced galectin-9 binding **(new Fig 5F) (line 280-line 285)**, further indicating the binding of galectin-9 to AG.

7.Why did the authors infect the zebrafish embryos in the Duct of Cuvier? This is a very unusual route of infection in zebrafish. They should consider injecting in the caudal vein (100 CFU) for systemic infection. Were the aptamers maintain in fish water during the whole experiment (14 days ?). Did the authors check for toxicity of the aptamers in zebrafish ? This is an important control to show.

We appreciate the reviewer's concern. For the Duct of Cuvier infection, we improved the zebrafish mounting by embedding them on a glass slide between two layers of low melting agarose, which greatly improved the efficacy and consistency for successful systemic infection (Benard, van der Sar et al., 2012, Hou, Sheng et al., 2016, Niu, Wang et al., 2019).

The aptamers were maintained in fish water during the whole experiment for 14 days. We detected the toxicity of the aptamers in zebrafish and found that the incubation with AG aptamers did not affect the survival of zebrafish embryos, indicating that AG aptamers alone are not toxic to zebrafish embryos. The data has been included in the revised manuscript **(Appendix Fig S2C) (line 177-line 181)**.

Referee #2:

The manuscript entitled "Sensing of Arabinogalactan by galectin exacerbates mycobacterial infection through inducing matrix metalloproteinases " describes

that Arabinogalactan (AG) in mycobacterial cell wall qualifies for being called as pathogen associated molecular pattern (PAMP) which is sensed by pathogen recognition receptor (PRR) galectin-9 and aggravates mycobacterial infection by inducing membrane metallic proteinases. The authors report that AG induces Galectin-9 association with TAK1 and subsequent activation of MAPKs as well as MMP production.
We thank the reviewer for the pertinent comments.

1) Galectin-9 is a soluble secreted molecule - and not a transmembrane protein as shown the model by the authors. A signaling domain has not been described. Galectin-3 primarily acts as a cytosolic sensor. How to the authors propose that galectin-9 leads to the activation of TAK1 pathway?
We appreciate the reviewer's insightful comments. As the reviewer pointed out, it has been reported that galectin-9 can be secreted or exert its function in the nucleus or cytoplasm (Heusschen, Freitag et al., 2013, Kanwar, Carone et al., 1997). Of note, galectin-9 has also been demonstrated to sense lysosomal damage signaling and to form a complex with TAK1(Jia, Abudu et al., 2018, Jia, Abudu et al., 2019, Jia, Bissa et al., 2020). Increasing evidence demonstrated that galectins are important for the formation of signalosomes and subsequent signaling activation (Laderach, Compagno et al., 2010). By performing confocal microscopy assays, we observed colocalization of galectin-9 with TAK1 in the cytosol in response to AG stimulation or *Mtb* infection **(Fig 7D)**, indicating that galectin-9 is a cytosolic receptor of AG. Moreover, by generating truncated mutants of galectin-9, co-immunoprecipitation assay demonstrated that CRD2, but not CRD1 or the linker region of galectin-9 mediated its interaction with TAK1 **(new Fig EV4B and C)**. In combination with the finding that AG stimulation or *Mtb* infection enhanced the interaction of galectin-9 with TAK1 **(Fig 7A, B and D)**, we, therefore, propose that binding of AG to galectin-9 CRD2 domain led to conformational changes of CRD2, which in turn enhanced the recruitment of TAK1 and the formation of a galetin-9/TAK1 signaling complex, leading to subsequent activation of ERK/MMPs signaling axis **(line 321-line 331)**.

2) When Mtb infects, Gal9 could possibly bind to Mtb, As such, there might be Gal9 bound to Mtb in the phagosome. How then, does Gal9 signal via TAK1, which is in the cytosol?
We appreciate the reviewer's concern. *Mtb* is an intracellular bacterium and principally can be delivered to the lysosome for clearance (Franco, Nair et al., 2017, Manzanillo, Ayres et al., 2013, Watson, Manzanillo et al., 2012). However, *Mtb* develops multiple strategies to prevent the maturation of phagosomes or the fusion with lysosomes and thereby it can persist in the phagosome (Carranza & Chavez-Galan, 2019, Chai, Wang et al., 2020, Levitte, Adams et al., 2016). The harsh environment in phagosome or lysosome may lead to shedding of AG from *Mtb* cell wall and leakage of AG into the cytosol via the damaged membrane, which leads to the engagement of galectin-9 and the activation of TAK1 in the cytosol **(line 464-line 472)**.

3) Is there any evidence that Mtb infection of macrophages stimulates TAK1?
We thank the reviewer for the question. TAK1 plays a key role in cellular responses to a variety of stimuli by triggering the activation of downstream effectors (Ajibade, Wang et al., 2013). Our very recent work demonstrated that TAK1 is important for the mounting of protective immunity against *Mtb* infection by inducing both Il6 and Il12b (Zheng, Liu et al., 2018). Moreover, TAK1 can be activated by *Mtb* infection through oxidation by *Mtb* secreted protein MPT53 (Wang, Liu et al., 2019). Our work further demonstrates that the AG-galectin-9 axis is critical for the activation of TAK1, adding a novel layer to the complexity of TAK1 regulation. Moreover, we demonstrated that the galectin-9-TAK1 pathway is utilized by AG (as an *Mtb* virulence factor that causes lung injury) by inducing MMPs, indicating a detrimental role of TAK1 in the process of *Mtb* infection **(line 427-line 436)**.

4) As galectin-9 binds carbohydrates, it's binding it promiscuous and there are many different proteins it can bind and has important interactions with host proteins. The finding that a Gal9 ko mouse differs in disease following infection or treatment with AG does not lead to the conclusion that the disease requires AG/Gal9 interaction. Certainly, macrophage infection and intracellular growth is normal in the absence of galectin-9 as has been previously published.
We appreciate the reviewer's concern. Indeed, our data demonstrate that galectin-9 is critical for AG-induced TAK1-ERK-MMPs signaling activation and lung damage. Moreover, we provide solid evidence supporting the interaction of AG with galectin-9. Mapping the binding sites of galectin-9 with AG and the generation of corresponding galectin-9 mutant knockin mice will help to address whether AG-Galectin-9 interaction is critical for the observed phenotypes in galectin-9 knockout mice following infection or treatment with AG. Our preliminary work demonstrated that CRD2 is critical for mediating AG-galectin-9 interaction, which also mediates TAK1 interaction. However, it still warrants further investigation to determine the amino acid residues located in CRD2 of galectin-9 that is essential for mediating its interaction with AG, which is beyond the scope of our current study **(line 324-line 327)**.

5) The finding described in the manuscript are not novel in terms of AG sensing by Galectins. The studies by Erminia Barboni et al in the manuscript "The binding of mycolic acids to galectin-3: A novel interaction between a host soluble lectin and trafficking mycobacterial lipids? has already shown direct interaction of mycolic acids (that contains and is bound to AG) with galectin-3. The authors should cite and discuss this paper.
We appreciate the reviewer's concern. As the reviewer pointed out, mycolic acid is constituted by long-chain α-alkyl-β-hydroxy fatty acids (C_{70-90}) and forms an integral component of the mycolyl-arabinogalactan-peptidoglycan (mAGP) complex (Liu, Barry et al., 1996). Although it has been reported that mycolic acids bind to galectin-3 (Barboni, Coade et al., 2005), there is no evidence demonstrating the direct interaction of galectin with AG itself. In this study, we took advantage of the first complete synthesis of a mycobacterial AG composed of 92 mono-saccharide units(Wu, Xiong et

al., 2017), and harnessed the chemically synthesized AG for the evaluation of its biological functions. Our work demonstrates that AG interacted with galectin-9 directly, which for the first time provides solid evidence for the interaction of AG with the host lectin galectin-9. We have included related information in the discussion part **(line 470-line 478)**.

6) The term AG as a PAMP is a misnomer. As per the results in the manuscript intraperitoneal or intravenous administration of AG cause lung injury and induces MMPs. Moreover, AG-specific aptamers extended the survival of Mtb-infected SCID mice or M. marinum-infected zebrafish. The data presented demonstrate AG as virulence factor and not PAMP. There is no evidence of pro-inflammatory and protective immune response by host presented in the manuscript that makes AG qualifies for PAMP. The author mentions in line 331 to 334 in discussion AG is a virulence factor. It is confusing. AG seems to be a virulence factor deploying galectin-9 to establish infection by Mtb rather than providing anti-microbial immunity (refer reference 60).

We appreciate the reviewer's insightful comments. We fully agree that AG should be termed virulence factor rather than PAMP. Our work demonstrated that the engagement of galectin-9 by AG induces lung injury to establish infection rather than mounting protective anti-microbial immunity. We have changed our claim accordingly to the revised manuscript **(line 134-line 135; line 181-line 182; line 383-line 385; line 415-line 418)**.

7) The authors mentioned that treatment of AG leads to Galentin-9 upregulation also doesn't seems to convince the fact that Galectin-9 is a PRR and AG as PAMP. The Galectins expression fluctuates in many diseases and their expression is upregulated in many of diseases including cancer and bacterial infection.

We agree. As the reviewer points out, galectins expression fluctuates in many diseases, and their expression is upregulated in many diseases including cancer and bacterial infection (Chou, Chen et al., 2018, Moar & Tandon, 2021)**.** Besides, we agree that the upregulation of a protein in response to a specific stimulation does not qualify the protein as a PRR and the stimuli as a PAMP. In fact, AG did not significantly induce the upregulation of galectin-9 **(Fig 7A and B)**. Our work demonstrated that the engagement of galectin-9 by AG induces lung injury to establish infection rather than mounting protective anti-microbial immunity, and AG is, therefore, a virulence factor rather than PAMP **(line 318-line 321)**.

8) The infection model used in this paper is inappropriate. This is a model of lung injury and not Mtb pathogenesis

We appreciate the reviewer's concern. We fully agree that mouse *Mtb* infection by aerosol route using inhalation exposure system is more relevant to natural infection (Frenkel, Ackart et al., 2020, Plumlee, Duffy et al., 2021). However, due to the inaccessibility to the aerosol infection model, here we employed an intranasal infection model which has been reported previously (Biketov, Potapov et al., 2007, Wang, Zhong

et al., 2015, Yang, Sha et al., 2018). Moreover, our finding that AG is a virulence factor of *Mtb* that induces lung injury renders the intranasal infection model reasonable.

9) The results presented in the manuscript does for the first time demonstrates the Galectin -9 -TAK1 pathway utilized by AG as a Mtb virulence factor that causes lung injury by inducing MMPs. Using a combination of SPR for demonstrating interaction of AG and Galectin-9 and immune-precipitation of Galectin-9 and TAK1 demonstrates the pathway utilized.
We thank the reviewer for the appreciation of the novelty of our work.

10) Why in the galectin 9 ko is inflammation going down while CFU is going up?
We appreciate the reviewer's concern. Consistent with a previous report that galectin 9 KO slightly enhanced mycobacterial survival in macrophages (Jayaraman, Sada-Ovalle et al., 2010), our data demonstrate that deletion of *galectin-9* resulted in a moderate increase of bacterial burden in the lung **(Fig 8F)**. However, we found that the deletion of galectin-9 led to less inflammation as demonstrated by H&E staining **(Fig 8D and E)**. Therefore, we think it is reasonable to speculate that the reduced inflammatory responses in galectin-9 KO mice affect control of mycobacterial replication since multiple proinflammatory cytokines such as IL-6 and IL-1β have been well documented to be important for the control of mycobacterial growth(Ladel, Blum et al., 1997, Mayer-Barber, Andrade et al., 2014, Sousa, Ca et al., 2020, Wang, Wu et al., 2020). However, we cannot rule out the possibility that galectin-9 modulates inflammatory responses and bactericidal effect in an uncoupled manner. We discuss this issue in the revised manuscript **(line 496-line 507)**.

Minor points:

11) The survival analysis in Fig.1H, I is incorrect (the statistical test is overaggressive and clearly there is not survival advantage). There is clearly no different in MST.
We appreciate the reviewer's concern. As stated in the figure legends, we performed statistical analysis by One-way ANOVA followed by Gehan–Breslow–Wilcoxon test with Graphpad Prism8. According to the data, the differences between the groups in Fig.1H and Fig. 1I are statistically different. However, we agree that the survival advantage under AG aptamer treatment is moderate. We, therefore, have moved the data to the supplementary Figures **(revised Appendix Fig S2B and C)** and rephrase the conclusion accordingly.

12) In figure 3A, a lot of heterogeneity is seen in the GAPDH controls. For eg. comparing pERK in 0 time point versus 5 there is an increase in GAPDH and so is pERK, while in 0 and 15 the GAPDH seems to be equal and thus the pERK levels doesn't increase. Same is the case in time point 120, 240, and 600. A normalized quantitative bargraph of pERK with GAPDH alongside would be more convincing for the results presented. It could be possible that pERK levels

do increase.

We appreciate the reviewer's concern. Following the reviewer's suggestion, we have quantified the gray density of the blots. The quantification data revealed that AG stimulation led to an increase in pERK levels **(Fig R1)**.

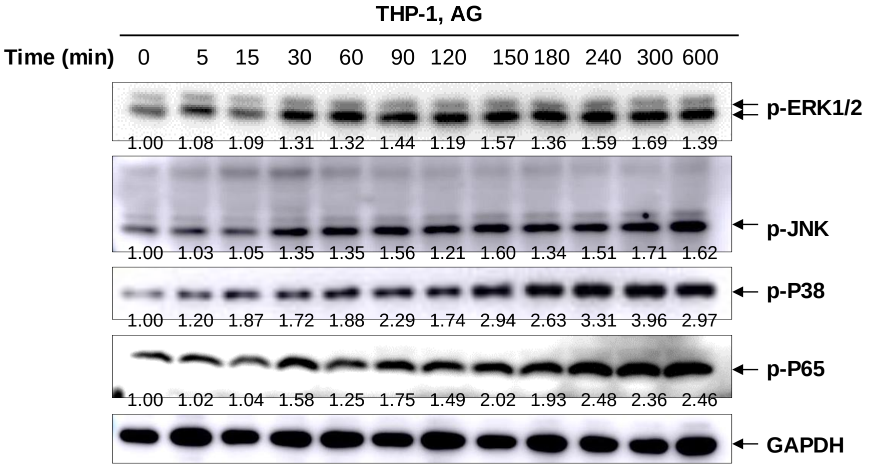

Figure R1. AG induces the activation of MAPKs and NF-κB. Immunoblotting of indicated proteins in the lysates of human THP-1 cells stimulated with AG for indicated times. The quantification data are shown as indicated.

13) In Fig.5 E/F, why is only a small portion of the molecules binding

We appreciate the reviewer's insightful question which is similar to question #6 raised by reviewer #1. *Mtb* possesses an unusual cell wall: the inner layer is peptidoglycan, the middle layer is highly branched AG and the outer layer is long-chain mycolic acid (Jankute et al., 2015). In **Fig. 5E-F (revised Fig 5D-E)**, to clarify whether galectin-9 binds to mycobacteria directly, we harvested mycobacteria from a log phase culture in the presence of Tween 80 and incubated these bacteria with galectins followed by FACS analysis. The detergent in the culture may disrupt the outer layer of the bacterial cell wall and thereby expose the AG for galectin-9 binding. Moreover, it has been reported that during replication, the bacteria may expose the newly synthesized AG (Meniche et al., 2014), which may in turn bind to galectin-9. Therefore, galectin-9 may specifically bind to mycobacteria with exposed AG by detergents or in replicating state. We discuss this issue in the revised manuscript **(line 457-line 481)**.

14) Fig 6F likely have labelling mistake, YFP-galectin 9 confused with GFP and IP and IB labels in the subsequent blots.

We appreciate the reviewer's concern. In fact, a yellow fluorescent protein (YFP) is a point mutant of green fluorescent protein (GFP) (Miyawaki, Llopis et al., 1997, Tsien, 1998). The antibody we used for the immunoprecipitation and immunoblotting is designed for targeting GFP which also recognizes YFP.

15) the authors don't mention the immunoprecipitation protocol in material and methods and its difficult to figure out the experiment for the reader. It needs to be corrected.

We appreciate the reviewer's concern. As per the reviewer's suggestion, we have included the immunoprecipitation protocol in the material and methods in the revised manuscript **(line 784-line 802)**.

16) The high dose IN Mtb infection model should be abandoned. With the pivot towards lower dose infection, it is unlikely to be telling us anything about the host-pathogen response.
We appreciate the reviewer's concern. Due to the inaccessibility to the aerosol infection model, here we employed an intranasal injection model which has been reported previously(Biketov et al., 2007, Wang et al., 2015, Yang et al., 2018). In fact, the infection dose we used in this study resulted in around 200 CFU in the lung of infected mice which is equivalent to generally used low dose aerosol infection (Moura-Alves, Fae et al., 2014). Moreover, our finding that AG is a virulence factor of *Mtb* that induces lung injury renders the intraperitoneal infection model reasonable.

Referee #3:

Major comments:
(1) The authors state that galectin-9 shows specific interaction with AG based by SPR assays (Figure 5 and EV3). However, this reviewer raised serious concerns about the quality of biochemical analyses. First, the sensorgram of galectin-9 and AG (Fig. 5A) shows very weak signals compared with the others (Fig. EV3). In addition, the fitting curves are significantly deviated from actual signals. Furthermore, the chi2 values of fitting curves should be clearly shown. Overall, this reviewer recommends the re-examination of these series of experiments under the appropriate conditions.
We appreciate the reviewer's concern. We repeated the SPR assay to detect the interaction of AG with galectin-9, the data consistently demonstrated strong binding signaling and the binding affinity is very high with a KD of 22.3 μM. The fitting curves have been re-analyzed. Moreover, the chi2 values of fitting curves have been provided and included in the revised Figures **(Fig 5A and 5C, Fig EV2C-J and L)**.

Second, the authors expressed CRD1 and 2 by using bacterial expression systems. Although the N-terminus of CRD1 starts at 16, the beta-sandwich fold of CRD1 originally starts at Ser6 in crystal structure. Weak sugar affinity of CRD1 might be derived from misfolding due to invalid construct design and it would not be appropriate to compare binding affinity using such construct.
We appreciate the reviewer's concern. We have constructed the plasmids including the N terminus of galectin-9 including amino acid 1-146 to avoid the destruction of the beta-sandwich fold of CRD1. By using a bacterial expression system, we successfully purified the recombinant protein galectin-9(1-146) **(new Fig EV2K)**. The SPR assay reveals that recombinant galectin-9(1-146) did not bind to AG **(new Fig EV2L)**, indicating that the weak AG sugar affinity of CRD1 **(Fig EV2J)** is not due to misfolding due to invalid construct design **(line 267-line 272)**.

Third, the authors showed the purity of CRD1 and 2 in Fig. 5B. However, there is no description regarding the purity of recombinant full-length galectin-1~14. The authors should show the purity of these commercial proteins as well.
We appreciate the reviewer's concern. As per the reviewer's suggestion, we have performed extra experiments to investigate the purity of recombinant full-length galectin-1, -3, -8, -7, 14 and LGALSL by SDS-PAGE and Coomassie blue staining. The data demonstrate that the purified recombinant proteins are of high purity **(new Fig EV2A)**.

(2) Considering well-studied galectin-9 structure and its galectin-binding mode, it is difficult to imagine that Galectin-9 can bind galactofranoside. Galectins specifically recognize beta-galactoside via highly conserved sugar binding sites. Within this binding sites, the OH4 and OH6 of beta-galactoside tightly interact with galectins. The authors described that galectin-9 binds to AG via beta-galactofuranose, though it lacks both OH4 and OH6 at the equivalent positions. The authors would be better to show feasible explanation with experimental data together with structural insight.
We appreciate the reviewer's concern. Our data demonstrated that galectin-9 interacts with AG which harbors beta-galactofuranoside rather than beta-galactopyranoside **(Fig 5A)**. As the reviewer pointed out, both OH4 and OH6 of beta- galactopyranoside are critical for its interaction with galectins (Chan, Lin et al., 2018). However, beta-galactofuranose lacks both OH4 and OH6 at the equivalent positions, indicating that beta-galactofuranoside may not mediate the interaction of AG with galectin-9. Of note, the chemically synthesized AG used in this study is a highly complex polysaccharide composed of 92 sugar units. It contains not only beta-galactofuranose residues, but also numerous alpha- and beta- arabinofuranose residues. Moreover, the tertiary structure of AG may be responsible for its interaction with galectin-9. However, the precise mechanisms underlying the interactions of AG with galectin-9 await the crystal structure analysis of the AG-galectin-9 complex **(line 483-line 495)**.

(3) The authors did not address the localization of this protein. How does the Galectin-9 bind to AG outside of the cells and thereafter associate with TAK in the cytoplasm. The author should provide reasonable experimental evidence to support the authors' conclusion in terms of protein trafficking.
Similar to question #1 raised by reviewer #2, we have performed extra experiments to investigate the colocalization of galectin-9 with TAK1 in macrophages. The data revealed an increased colocalization of galectin-9 with TAK1 in the cytosol in response to AG stimulation and *Mtb* infection **(new Fig7D)**. Therefore, galectin-9 may be a cytosolic receptor for AG derived from invading mycobacteria present in the phagosome or in the cytosol (Chai et al., 2020, Houben, Demangel et al., 2012, Simeone, Sayes et al., 2015, van der Wel, Hava et al., 2007). We therefore have revised the working model as shown in the **revised Appendix Fig S4**.

Minor comments:

(1) References were not appropriately cited.

We appreciate the reviewer's advice on the reference citations and we have included these references in the revised manuscript.

(2) The authors interpreted that the administration of aptamer against AG prolonged the survival upon Mtb infection. However, the marginal difference would be difficult to prove the authors' conclusion.

We appreciate the reviewer's concern similar to question #11 raised by reviewer #2. The data demonstrate that administration of aptamer against AG moderately prolonged survival of *Mtb*-infected SCID mice and zebrafish embryos **(revised Appendix Fig S2B and C)**. Although the difference is not profound, it is statistically significant. However, we agree that the advantages in the survival from AG aptamer are moderate. To avoid any overstatement, we have moved the data into the supplementary Figures and rephrased the conclusion accordingly.

(3) page 11, line 224, the authors' description "Therefore, our results suggest that galectin-9 acts as a specific PRR for AG." is overstated.

We appreciate the reviewer's concern. We have changed our claim: "… our results suggest that galectin-9 plays an important role in sensing of AG". This has been included in the revised manuscript **(line 284-line 285)**.

(4) It would be better to show reliable data that demonstrate the feasible mode of the interaction of Galectin-9 and TAK1.

We appreciate the reviewer's insightful comment. By generating the truncated mutants of galectin-9, co-immunoprecipitation assay demonstrated that CRD2, but not CRD1 or the linker region of galectin-9 mediated its interaction with TAK1 **(new Fig EV4B-C)**. In combination with the finding that AG stimulation or Mtb infection enhanced the interaction of galectin-9 with TAK1, we proposed that the binding of AG to galectin-9 CRD2 domain led to the conformational change of CRD2, which may, in turn, enhanced recruitment of TAK1 and subsequent activation of ERK/MMPs **(line 321-line 331)**.

References

Aimaiti R, Qin L, Cao T, Yang H, Wang J, Lu J, Huang X, Hu Z (2015) Identification and application of ssDNA aptamers against H(3)(7)Rv in the detection of Mycobacterium tuberculosis. *Appl Microbiol Biotechnol* 99: 9073-83

Ajibade AA, Wang HY, Wang RF (2013) Cell type-specific function of TAK1 in innate immune signaling. *Trends Immunol* 34: 307-16

Alderwick LJ, Harrison J, Lloyd GS, Birch HL (2015) The Mycobacterial Cell Wall--Peptidoglycan and Arabinogalactan. *Cold Spring Harb Perspect Med* 5: a021113

Barboni E, Coade S, Fiori A (2005) The binding of mycolic acids to galectin-3: a novel interaction between a host soluble lectin and trafficking mycobacterial lipids? *FEBS*

Lett 579: 6749-55
Bastos PAD, Wheeler R, Boneca IG (2021) Uptake, recognition and responses to peptidoglycan in the mammalian host. *FEMS Microbiol Rev* 45
Benard EL, van der Sar AM, Ellett F, Lieschke GJ, Spaink HP, Meijer AH (2012) Infection of zebrafish embryos with intracellular bacterial pathogens. *J Vis Exp*
Biketov S, Potapov V, Ganina E, Downing K, Kana BD, Kaprelyants A (2007) The role of resuscitation promoting factors in pathogenesis and reactivation of Mycobacterium tuberculosis during intra-peritoneal infection in mice. *BMC Infect Dis* 7: 146
Bock LC, Griffin LC, Latham JA, Vermaas EH, Toole JJ (1992) Selection of single-stranded DNA molecules that bind and inhibit human thrombin. *Nature* 355: 564-6
Carranza C, Chavez-Galan L (2019) Several Routes to the Same Destination: Inhibition of Phagosome-Lysosome Fusion by Mycobacterium tuberculosis. *Am J Med Sci* 357: 184-194
Chai Q, Wang L, Liu CH, Ge B (2020) New insights into the evasion of host innate immunity by Mycobacterium tuberculosis. *Cell Mol Immunol* 17: 901-913
Chan YC, Lin HY, Tu Z, Kuo YH, Hsu SD, Lin CH (2018) Dissecting the Structure-Activity Relationship of Galectin-Ligand Interactions. *Int J Mol Sci* 19
Chou FC, Chen HY, Kuo CC, Sytwu HK (2018) Role of Galectins in Tumors and in Clinical Immunotherapy. *Int J Mol Sci* 19
Dulberger CL, Rubin EJ, Boutte CC (2020) The mycobacterial cell envelope - a moving target. *Nat Rev Microbiol* 18: 47-59
Franco LH, Nair VR, Scharn CR, Xavier RJ, Torrealba JR, Shiloh MU, Levine B (2017) The Ubiquitin Ligase Smurf1 Functions in Selective Autophagy of Mycobacterium tuberculosis and Anti-tuberculous Host Defense. *Cell Host Microbe* 21: 59-72
Frenkel JDH, Ackart DF, Todd AK, DiLisio JE, Hoffman S, Tanner S, Kiran D, Murray M, Chicco A, Obregon-Henao A, Podell BK, Basaraba RJ (2020) Metformin enhances protection in guinea pigs chronically infected with Mycobacterium tuberculosis. *Sci Rep* 10: 16257
Golichenari B, Nosrati R, Farokhi-Fard A, Abnous K, Vaziri F, Behravan J (2018) Nano-biosensing approaches on tuberculosis: Defy of aptamers. *Biosens Bioelectron* 117: 319-331
Heusschen R, Freitag N, Tirado-Gonzalez I, Barrientos G, Moschansky P, Munoz-Fernandez R, Leno-Duran E, Klapp BF, Thijssen VL, Blois SM (2013) Profiling Lgals9 splice variant expression at the fetal-maternal interface: implications in normal and pathological human pregnancy. *Biol Reprod* 88: 22
Hou Y, Sheng Z, Mao X, Li C, Chen J, Zhang J, Huang H, Ruan H, Luo L, Li L (2016) Systemic inoculation of Escherichia coli causes emergency myelopoiesis in zebrafish larval caudal hematopoietic tissue. *Sci Rep* 6: 36853
Houben D, Demangel C, van Ingen J, Perez J, Baldeon L, Abdallah AM, Caleechurn L, Bottai D, van Zon M, de Punder K, van der Laan T, Kant A, Bossers-de Vries R, Willemsen P, Bitter W, van Soolingen D, Brosch R, van der Wel N, Peters PJ (2012) ESX-1-mediated translocation to the cytosol controls virulence of mycobacteria. *Cell Microbiol* 14: 1287-98
Jankute M, Cox JA, Harrison J, Besra GS (2015) Assembly of the Mycobacterial Cell

Wall. *Annu Rev Microbiol* 69: 405-23
Jayaraman P, Sada-Ovalle I, Beladi S, Anderson AC, Dardalhon V, Hotta C, Kuchroo VK, Behar SM (2010) Tim3 binding to galectin-9 stimulates antimicrobial immunity. *J Exp Med* 207: 2343-54
Jia J, Abudu YP, Claude-Taupin A, Gu Y, Kumar S, Choi SW, Peters R, Mudd MH, Allers L, Salemi M, Phinney B, Johansen T, Deretic V (2018) Galectins Control mTOR in Response to Endomembrane Damage. *Mol Cell* 70: 120-135 e8
Jia J, Abudu YP, Claude-Taupin A, Gu Y, Kumar S, Choi SW, Peters R, Mudd MH, Allers L, Salemi M, Phinney B, Johansen T, Deretic V (2019) Galectins control MTOR and AMPK in response to lysosomal damage to induce autophagy. *Autophagy* 15: 169-171
Jia J, Bissa B, Brecht L, Allers L, Choi SW, Gu Y, Zbinden M, Burge MR, Timmins G, Hallows K, Behrends C, Deretic V (2020) AMPK, a Regulator of Metabolism and Autophagy, Is Activated by Lysosomal Damage via a Novel Galectin-Directed Ubiquitin Signal Transduction System. *Mol Cell* 77: 951-969 e9
Kanwar YS, Carone FA, Kumar A, Wada J, Ota K, Wallner EI (1997) Role of extracellular matrix, growth factors and proto-oncogenes in metanephric development. *Kidney Int* 52: 589-606
Kathamuthu GR, Kumar NP, Moideen K, Nair D, Banurekha VV, Sridhar R, Baskaran D, Babu S (2020) Matrix Metalloproteinases and Tissue Inhibitors of Metalloproteinases Are Potential Biomarkers of Pulmonary and Extra-Pulmonary Tuberculosis. *Front Immunol* 11: 419
Korf J, Stoltz A, Verschoor J, De Baetselier P, Grooten J (2005) The Mycobacterium tuberculosis cell wall component mycolic acid elicits pathogen-associated host innate immune responses. *Eur J Immunol* 35: 890-900
Ladel CH, Blum C, Dreher A, Reifenberg K, Kopf M, Kaufmann SH (1997) Lethal tuberculosis in interleukin-6-deficient mutant mice. *Infect Immun* 65: 4843-9
Laderach DJ, Compagno D, Toscano MA, Croci DO, Dergan-Dylon S, Salatino M, Rabinovich GA (2010) Dissecting the signal transduction pathways triggered by galectin-glycan interactions in physiological and pathological settings. *IUBMB Life* 62: 1-13
Levitte S, Adams KN, Berg RD, Cosma CL, Urdahl KB, Ramakrishnan L (2016) Mycobacterial Acid Tolerance Enables Phagolysosomal Survival and Establishment of Tuberculous Infection In Vivo. *Cell Host Microbe* 20: 250-8
Liu J, Barry CE, 3rd, Besra GS, Nikaido H (1996) Mycolic acid structure determines the fluidity of the mycobacterial cell wall. *J Biol Chem* 271: 29545-51
Lu Y, Zheng Y, Coyaud E, Zhang C, Selvabaskaran A, Yu Y, Xu Z, Weng X, Chen JS, Meng Y, Warner N, Cheng X, Liu Y, Yao B, Hu H, Xia Z, Muise AM, Klip A, Brumell JH, Girardin SE et al. (2019) Palmitoylation of NOD1 and NOD2 is required for bacterial sensing. *Science* 366: 460-467
Manzanillo PS, Ayres JS, Watson RO, Collins AC, Souza G, Rae CS, Schneider DS, Nakamura K, Shiloh MU, Cox JS (2013) The ubiquitin ligase parkin mediates resistance to intracellular pathogens. *Nature* 501: 512-6
Mayer-Barber KD, Andrade BB, Oland SD, Amaral EP, Barber DL, Gonzales J, Derrick

SC, Shi R, Kumar NP, Wei W, Yuan X, Zhang G, Cai Y, Babu S, Catalfamo M, Salazar AM, Via LE, Barry CE, 3rd, Sher A (2014) Host-directed therapy of tuberculosis based on interleukin-1 and type I interferon crosstalk. *Nature* 511: 99-103

Meniche X, Otten R, Siegrist MS, Baer CE, Murphy KC, Bertozzi CR, Sassetti CM (2014) Subpolar addition of new cell wall is directed by DivIVA in mycobacteria. *Proc Natl Acad Sci U S A* 111: E3243-51

Miyawaki A, Llopis J, Heim R, McCaffery JM, Adams JA, Ikura M, Tsien RY (1997) Fluorescent indicators for Ca2+ based on green fluorescent proteins and calmodulin. *Nature* 388: 882-7

Moar P, Tandon R (2021) Galectin-9 as a biomarker of disease severity. *Cell Immunol* 361: 104287

Moura-Alves P, Fae K, Houthuys E, Dorhoi A, Kreuchwig A, Furkert J, Barison N, Diehl A, Munder A, Constant P, Skrahina T, Guhlich-Bornhof U, Klemm M, Koehler AB, Bandermann S, Goosmann C, Mollenkopf HJ, Hurwitz R, Brinkmann V, Fillatreau S et al. (2014) AhR sensing of bacterial pigments regulates antibacterial defence. *Nature* 512: 387-92

Niu L, Wang C, Zhang K, Kang M, Liang R, Zhang X, Yan B (2019) Visualization of Macrophage Lytic Cell Death During Mycobacterial Infection in Zebrafish Embryos via Intravital Microscopy. *J Vis Exp*

Ong CW, Elkington PT, Friedland JS (2014) Tuberculosis, pulmonary cavitation, and matrix metalloproteinases. *Am J Respir Crit Care Med* 190: 9-18

Parasa VR, Muvva JR, Rose JF, Braian C, Brighenti S, Lerm M (2017) Inhibition of Tissue Matrix Metalloproteinases Interferes with Mycobacterium tuberculosis-Induced Granuloma Formation and Reduces Bacterial Load in a Human Lung Tissue Model. *Front Microbiol* 8: 2370

Plumlee CR, Duffy FJ, Gern BH, Delahaye JL, Cohen SB, Stoltzfus CR, Rustad TR, Hansen SG, Axthelm MK, Picker LJ, Aitchison JD, Sherman DR, Ganusov VV, Gerner MY, Zak DE, Urdahl KB (2021) Ultra-low Dose Aerosol Infection of Mice with Mycobacterium tuberculosis More Closely Models Human Tuberculosis. *Cell Host Microbe* 29: 68-82 e5

Qin LH, Liu ZH, Yang H, Cai JL, Bai WJ, Wang J, Liu JM, Hu ZY (2014) Dynamic evolution and immunoreactivity of aptamers binding to polyclonal antibodies against MPT64 antigen of Mycobacterium tuberculosis. *Eur J Clin Microbiol Infect Dis* 33: 1199-209

Sabir N, Hussain T, Mangi MH, Zhao D, Zhou X (2019) Matrix metalloproteinases: Expression, regulation and role in the immunopathology of tuberculosis. *Cell Prolif* 52: e12649

Simeone R, Sayes F, Song O, Groschel MI, Brodin P, Brosch R, Majlessi L (2015) Cytosolic access of Mycobacterium tuberculosis: critical impact of phagosomal acidification control and demonstration of occurrence in vivo. *PLoS Pathog* 11: e1004650

Sousa J, Ca B, Maceiras AR, Simoes-Costa L, Fonseca KL, Fernandes AI, Ramos A, Carvalho T, Barros L, Magalhaes C, Chiner-Oms A, Machado H, Veiga MI, Singh A, Pereira R, Amorim A, Vieira J, Vieira CP, Bhatt A, Rodrigues F et al. (2020)

Mycobacterium tuberculosis associated with severe tuberculosis evades cytosolic surveillance systems and modulates IL-1beta production. *Nat Commun* 11: 1949

Sun X, Pan Q, Yuan C, Wang Q, Tang XL, Ding K, Zhou X, Zhang XL (2016) A Single ssDNA Aptamer Binding to Mannose-Capped Lipoarabinomannan of Bacillus Calmette-Guerin Enhances Immunoprotective Effect against Tuberculosis. *J Am Chem Soc* 138: 11680-9

Tahiri N, Fodran P, Jayaraman D, Buter J, Witte MD, Ocampo TA, Moody DB, Van Rhijn I, Minnaard AJ (2020) Total Synthesis of a Mycolic Acid from Mycobacterium tuberculosis. *Angew Chem Int Ed Engl* 59: 7555-7560

Tang XL, Wu SM, Xie Y, Song N, Guan Q, Yuan C, Zhou X, Zhang XL (2016) Generation and application of ssDNA aptamers against glycolipid antigen ManLAM of Mycobacterium tuberculosis for TB diagnosis. *J Infect* 72: 573-86

Tsien RY (1998) The green fluorescent protein. *Annu Rev Biochem* 67: 509-44

Tuerk C, Gold L (1990) Systematic evolution of ligands by exponential enrichment: RNA ligands to bacteriophage T4 DNA polymerase. *Science* 249: 505-10

van der Wel N, Hava D, Houben D, Fluitsma D, van Zon M, Pierson J, Brenner M, Peters PJ (2007) M. tuberculosis and M. leprae translocate from the phagolysosome to the cytosol in myeloid cells. *Cell* 129: 1287-98

Verschoor JA, Baird MS, Grooten J (2012) Towards understanding the functional diversity of cell wall mycolic acids of Mycobacterium tuberculosis. *Prog Lipid Res* 51: 325-39

Volkman HE, Pozos TC, Zheng J, Davis JM, Rawls JF, Ramakrishnan L (2010) Tuberculous granuloma induction via interaction of a bacterial secreted protein with host epithelium. *Science* 327: 466-9

Wang L, Liu Z, Wang J, Liu H, Wu J, Tang T, Li H, Yang H, Qin L, Ma D, Chen J, Liu F, Wang P, Zheng R, Song P, Zhou Y, Cui Z, Wu X, Huang X, Liang H et al. (2019) Oxidization of TGFbeta-activated kinase by MPT53 is required for immunity to Mycobacterium tuberculosis. *Nat Microbiol* 4: 1378-1388

Wang L, Wu J, Li J, Yang H, Tang T, Liang H, Zuo M, Wang J, Liu H, Liu F, Chen J, Liu Z, Wang Y, Peng C, Wu X, Zheng R, Huang X, Ran Y, Rao Z, Ge B (2020) Host-mediated ubiquitination of a mycobacterial protein suppresses immunity. *Nature* 577: 682-688

Wang Y, Zhong H, Xie X, Chen CY, Huang D, Shen L, Zhang H, Chen ZW, Zeng G (2015) Long noncoding RNA derived from CD244 signaling epigenetically controls CD8+ T-cell immune responses in tuberculosis infection. *Proc Natl Acad Sci U S A* 112: E3883-92

Watson RO, Manzanillo PS, Cox JS (2012) Extracellular M. tuberculosis DNA targets bacteria for autophagy by activating the host DNA-sensing pathway. *Cell* 150: 803-15

Wolf AJ, Reyes CN, Liang W, Becker C, Shimada K, Wheeler ML, Cho HC, Popescu NI, Coggeshall KM, Arditi M, Underhill DM (2016) Hexokinase Is an Innate Immune Receptor for the Detection of Bacterial Peptidoglycan. *Cell* 166: 624-636

Wolf AJ, Underhill DM (2018) Peptidoglycan recognition by the innate immune system. *Nat Rev Immunol* 18: 243-254

Wu Y, Xiong DC, Chen SC, Wang YS, Ye XS (2017) Total synthesis of mycobacterial

arabinogalactan containing 92 monosaccharide units. *Nat Commun* 8: 14851
Yang H, Sha W, Liu Z, Tang T, Liu H, Qin L, Cui Z, Chen J, Liu F, Zheng R, Huang X, Wang J, Feng Y, Ge B (2018) Lysine acetylation of DosR regulates the hypoxia response of Mycobacterium tuberculosis. *Emerg Microbes Infect* 7: 34
Zhang L, Zhao Y, Gao Y, Wu L, Gao R, Zhang Q, Wang Y, Wu C, Wu F, Gurcha SS, Veerapen N, Batt SM, Zhao W, Qin L, Yang X, Wang M, Zhu Y, Zhang B, Bi L, Zhang X et al. (2020) Structures of cell wall arabinosyltransferases with the anti-tuberculosis drug ethambutol. *Science* 368: 1211-1219
Zhang X, Feng Y, Yao Q, He F (2017) Selection of a new Mycobacterium tuberculosis H37Rv aptamer and its application in the construction of a SWCNT/aptamer/Au-IDE MSPQC H37Rv sensor. *Biosens Bioelectron* 98: 261-266
Zheng R, Liu H, Zhou Y, Yan D, Chen J, Ma D, Feng Y, Qin L, Liu F, Huang X, Wang J, Ge B (2018) Notch4 Negatively Regulates the Inflammatory Response to Mycobacterium tuberculosis Infection by Inhibiting TAK1 Activation. *J Infect Dis* 218: 312-323

1st Revision - Editorial Decision 24th Mar 2021

Dear Dr. Liu,

Thank you for the submission of your revised manuscript to our editorial offices. We have now received the reports from the three referees that were asked to re-evaluate your study, you will find below. As you will see, the referees #1 and #2 now support the publication of your study in EMBO reports. Nevertheless, referee #3 has remaining concerns and suggestions to improve the manuscript, we ask you to address in a final revised manuscript and provide more data regarding the relationship between AG binding and phosphorylation of TAK1. Please also provide a point-by-point-response addressing the remaining concerns of referee #3.

Moreover, I have these editorial requests I also ask you to address in a final revised manuscript:

- Please shorten the title to not more than 100 characters.

- Please provide the abstract written in present tense and with not more than 175 words.

- Please call the 'competing interest statement' 'Conflict of interest statement'.

- Please add page numbers and a table of contents (TOC) including page numbers to the Appendix.

- Please remove the statement 'Expanded View for this article is available online' from the manuscript text.

- Please add a proper table of contents (TOC) to the Appendix file. The present one is incomprehensible.

- It seems authors Fen Tang, Hua Yang and Yajuan Cao are missing from the author contributions. There are 2 JCs present. Please distinguish these, e.g. using JianC and JiayC.

- Please format the references according to our new reference format (if more than 10 authors use et al.):
http://www.embopress.org/page/journal/14693178/authorguide#referencesformat

- Thank you for submitting the source data. Please submit the source data as one PDF file per figure (main and EV figures).

- Please make sure that all the funding information is entered also into the online submission system and is complete and similar to the one in the manuscript text file.

- Finally, please find attached a word file of the manuscript text (provided by our publisher) with changes we ask you to include in your final manuscript text, and some queries, we ask you to address. Please provide your final manuscript file with track changes, in order that we can see any modifications done.

Please note that all corresponding authors are required to supply an ORCID ID for their name upon submission of a revised manuscript. Please do that for co-corresponding author Baoxue Ge. Please find instructions on how to link the ORCID ID to the account in our manuscript tracking system in

our Author guidelines:
http://www.embopress.org/page/journal/14693178/authorguide#authorshipguidelines

In addition, I would need from you:
- a short, two-sentence summary of the manuscript (not more than 35 words).
- two to three bullet points highlighting the key findings of your study
- a schematic summary figure (in jpeg or tiff format with the exact width of 550 pixels and a height of not more than 400 pixels) that can be used as a visual synopsis on our website.

I look forward to seeing the final revised version of your manuscript when it is ready. Please let me know if you have questions regarding the revision.

Yours sincerely,

Achim Breiling
Editor
EMBO Reports

Referee #1:

In this new version of the manuscript, Wu et al. provide compelling evidence that arabinogalactan (AG) serves as a PAMP which is recognized by galectin-9, exacerbating the infection and pathology of TB. The results clearly indicate that this interaction requires the CRD2 domain of galectin-9, leading to the activation of the TAK-1 pathway and the induction of matrix metalloproteinases, known to participate in pulmonary tissue degradation. These processes were reduced in the presence of AG-specific aptamers, opening the way to the rational design of new host-directed therapy as adjuncts to standard chemotherapy for the treatment of TB. The manuscript is well written and the conclusions supported by a large set of finding and combining a wide panel of complementary approaches with appropriate controls. The authors have satisfactorily responded to all my previous comments.

Referee #2:

Although the authors agreed in their response that the issue of whether AG is a virulence factor or a PAMP confuses the issue, and they have modified their manuscript accordingly, is still stated in the abstract "Yet, it is unclear whether mycobacterial AG is a pathogen-associated molecular pattern (PAMP) with an elusive pattern recognition receptor (PRR)." This line should be removed.

Referee #3:

The authors responded each point and performed additional experiments as the reviewer indicated. However, the fundamental questions have still remained. For example, how the interaction between AG and galectin-9 induces downstream signaling? Does AG enter the cytosolic side? (but how?) Leastwise, the author should clarify the relationship between the binding with AG and phosphorylation of TAK1.

Point to point response

We are very grateful for the reviewers' constructive and encouraging comments. We have revised the manuscript (with tracking changes) and hope to address each of the concerns from the reviewers.

REVIEWERS' COMMENTS:

Referee #1:

In this new version of the manuscript, Wu et al. provide compelling evidence that arabinogalactan (AG) serves as a PAMP which is recognized by galectin-9, exacerbating the infection and pathology of TB. The results clearly indicate that this interaction requires the CRD2 domain of galectin-9, leading to the activation of the TAK-1 pathway and the induction of matrix metalloproteinases, known to participate in pulmonary tissue degradation. These processes were reduced in the presence of AG-specific aptamers, opening the way to the rational design of new host-directed therapy as adjuncts to standard chemotherapy for the treatment of TB. The manuscript is well written and the conclusions supported by a large set of finding and combining a wide panel of complementary approaches with appropriate controls. The authors have satisfactorily responded to all my previous comments.

We thank the reviewer for the encouraging comments.

Referee #2:

Although the authors agreed in their response that the issue of whether AG is a virulence factor or a PAMP confuses the issue, and they have modified their manuscript accordingly, is still stated in the abstract "Yet, it is unclear whether mycobacterial AG is a pathogen-associated molecular pattern (PAMP) with an elusive pattern recognition receptor (PRR)." This line should be removed.

We thank the reviewer for the correction. Those statement has been removed in the revised abstract.

Referee #3:

The authors responded each point and performed additional experiments as the reviewer indicated. However, the fundamental questions have still remained. For example, how the interaction between AG and galectin-9 induces downstream signaling? Does AG enter the cytosolic side? (but how?) Leastwise, the author should clarify the relationship between the binding with AG and phosphorylation of TAK1.

We appreciate the reviewer's concern. We found that AG stimulation or *Mtb* infection enhanced the interaction of galectin-9 with TAK1 **(Fig 7A-B and D)**. Moreover, by generating the truncated mutants of galectin-9, co-immunoprecipitation assay demonstrated that CRD2, but not CRD1 or the linker region of galectin-9 mediated its interaction with TAK1 **(Fig EV4B-C)**. We therefore proposed that the binding of AG to galectin-9 CRD2 domain led to the conformational change of CRD2, which may in turn enhanced recruitment of TAK1 and subsequent activation of ERK/MMPs **(line 356-line 365)**.

We propose that galectin-9 senses AG in the cytosol. During the process of *Mtb* infection, the harsh environment in phagosome or lysosome may lead to the shedding of AG from *Mtb* cell wall and the leakage of AG into the cytosol via the damaged membrane. *Mtb* may also escape into the cytosol and the replicating bacteria may expose the newly synthesized AG. Our data also demonstrated that the addition of AG in the culture is sufficient for the activation of TAK1 and downstream signaling in macrophages (**Fig 7E-F**) and HEK23T cells **(new Supplementary Fig EV5E)**, indicating that endocytosis of AG may be critical for the access of AG to the cytosolic side. However, this warrants further investigations.

To clarify the relationship between the binding with AG and phosphorylation of TAK1, we detected whether CRD2 of galectin-9, which is critical for its interaction with AG and TAK1 **(Fig 5C and Fig EV4C)**, is sufficient for TAK1 activation. Overexpression of galectin-9 enhanced AG-induced TAK1 and ERK activation in HEK293T cells **(new Supplementary Fig EV5F)**. Of note, overexpression of CRD2 instead of CRD1 rendered the activation of TAK1 and ERK in HEK293T cells **(new Supplementary Fig EV5F)**. Therefore, the formation of AG-galectin-9-TAK1 complex is critical for the activation of TAK1 and downstream signaling. We have included the new data in the revised manuscript.

2nd Revision - Editorial Decision 16th Apr 2021

Dr. Haipeng Liu
Shanghai Key Lab of Tuberculosis, Tongji University School of Medicine
Shanghai
China

Dear Dr. Liu,

I am very pleased to accept your manuscript for publication in the next available issue of EMBO reports. Thank you for your contribution to our journal.

At the end of this email I include important information about how to proceed. Please ensure that you take the time to read the information and complete and return the necessary forms to allow us to publish your manuscript as quickly as possible.

As part of the EMBO publication's Transparent Editorial Process, EMBO reports publishes online a Review Process File to accompany accepted manuscripts. As you are aware, this File will be published in conjunction with your paper and will include the referee reports, your point-by-point response and all pertinent correspondence relating to the manuscript.

If you do NOT want this File to be published, please inform the editorial office within 2 days, if you have not done so already, otherwise the File will be published by default [contact: emboreports@embo.org]. If you do opt out, the Review Process File link will point to the following statement: "No Review Process File is available with this article, as the authors have chosen not to make the review process public in this case."

Should you be planning a Press Release on your article, please get in contact with emboreports@wiley.com as early as possible, in order to coordinate publication and release dates.

Thank you again for your contribution to EMBO reports and congratulations on a successful publication. Please consider us again in the future for your most exciting work.

Yours sincerely,

Achim Breiling
Editor
EMBO Reports

****

THINGS TO DO NOW:

You will receive proofs by e-mail approximately 2-3 weeks after all relevant files have been sent to our Production Office; you should return your corrections within 2 days of receiving the proofs.

Please inform us if there is likely to be any difficulty in reaching you at the above address at that time. Failure to meet our deadlines may result in a delay of publication, or publication without your corrections.

All further communications concerning your paper should quote reference number EMBOR-2020-51678V3 and be addressed to emboreports@wiley.com.

Should you be planning a Press Release on your article, please get in contact with emboreports@wiley.com as early as possible, in order to coordinate publication and release dates.

EMBO PRESS

YOU MUST COMPLETE ALL CELLS WITH A PINK BACKGROUND ↓

PLEASE NOTE THAT THIS CHECKLIST WILL BE PUBLISHED ALONGSIDE YOUR PAPER

Corresponding Author Name: Baoxue Ge

Journal Submitted to: EMBO Reports
Manuscript Number:EMBOR-2020-51678V2

Reporting Checklist For Life Sciences Articles (Rev. June 2017)

This checklist is used to ensure good reporting standards and to improve the reproducibility of published results. These guidelines are consistent with the Principles and Guidelines for Reporting Preclinical Research issued by the NIH in 2014. Please follow the journal's authorship guidelines in preparing your manuscript.

A- Figures
1. Data
The data shown in figures should satisfy the following conditions:

- the data were obtained and processed according to the field's best practice and are presented to reflect the results of the experiments in an accurate and unbiased manner.
- figure panels include only data points, measurements or observations that can be compared to each other in a scientifically meaningful way.
- graphs include clearly labeled error bars for independent experiments and sample sizes. Unless justified, error bars should not be shown for technical replicates.
- if n< 5, the individual data points from each experiment should be plotted and any statistical test employed should be justified
- Source Data should be included to report the data underlying graphs. Please follow the guidelines set out in the author ship guidelines on Data Presentation.

2. Captions
Each figure caption should contain the following information, for each panel where they are relevant:

- a specification of the experimental system investigated (eg cell line, species name).
- the assay(s) and method(s) used to carry out the reported observations and measurements
- an explicit mention of the biological and chemical entity(ies) that are being measured.
- an explicit mention of the biological and chemical entity(ies) that are altered/varied/perturbed in a controlled manner.

- the exact sample size (n) for each experimental group/condition, given as a number, not a range;
- a description of the sample collection allowing the reader to understand whether the samples represent technical or biological replicates (including how many animals, litters, cultures, etc.).
- a statement of how many times the experiment shown was independently replicated in the laboratory.
- definitions of statistical methods and measures:
  - common tests, such as t-test (please specify whether paired vs. unpaired), simple χ2 tests, Wilcoxon and Mann-Whitney tests, can be unambiguously identified by name only, but more complex techniques should be described in the methods section;
  - are tests one-sided or two-sided?
  - are there adjustments for multiple comparisons?
  - exact statistical test results, e.g., P values = x but not P values < x;
  - definition of 'center values' as median or average;
  - definition of error bars as s.d. or s.e.m.

Any descriptions too long for the figure legend should be included in the methods section and/or with the source data.

In the pink boxes below, please ensure that the answers to the following questions are reported in the manuscript itself. Every question should be answered. If the question is not relevant to your research, please write NA (non applicable). We encourage you to include a specific subsection in the methods section for statistics, reagents, animal models and human subjects.

USEFUL LINKS FOR COMPLETING THIS FORM

http://www.antibodypedia.com
http://1degreebio.org
http://www.equator-network.org/reporting-guidelines/improving-bioscience-research-repor
http://grants.nih.gov/grants/olaw/olaw.htm
http://www.mrc.ac.uk/Ourresearch/Ethicsresearchguidance/Useofanimals/index.htm
http://ClinicalTrials.gov
http://www.consort-statement.org
http://www.consort-statement.org/checklists/view/32-consort/66-title
http://www.equator-network.org/reporting-guidelines/reporting-recommendations-for-tumo
http://datadryad.org
http://figshare.com
http://www.ncbi.nlm.nih.gov/gap
http://www.ebi.ac.uk/ega
http://biomodels.net/
http://biomodels.net/miriam/
http://jjj.biochem.sun.ac.za
http://oba.od.nih.gov/biosecurity/biosecurity_documents.html
http://www.selectagents.gov/

B- Statistics and general methods

Please fill out these boxes ↓ (Do not worry if you cannot see all your text once you press return)

1.a. How was the sample size chosen to ensure adequate power to detect a pre-specified effect size?	No statistical methods were used to predetermine sample sizes. Sample sizes were selected based on the previous experimental experience with similar assays, and/or on sizes generally employed in the field. At least three biological replicates per experiment had been used in our study.
1.b. For animal studies, include a statement about sample size estimate even if no statistical methods were used.	Details are mentioned in the materials and methods section.
2. Describe inclusion/exclusion criteria if samples or animals were excluded from the analysis. Were the criteria pre-established?	No samples were excluded from the analysis.
3. Were any steps taken to minimize the effects of subjective bias when allocating animals/samples to treatment (e.g. randomization procedure)? If yes, please describe.	To minimize the effects of subjective bias, Female mice (6-8 weeks old) were divided randomly into cages upon arrival and were challenged by intraperitoneal or intravenous administration of AG
For animal studies, include a statement about randomization even if no randomization was used.	Details are mentioned in the materials and methods section.
4.a. Were any steps taken to minimize the effects of subjective bias during group allocation or/and when assessing results (e.g. blinding of the investigator)? If yes please describe.	The investigators were blinded during data collection and results analysis.
4.b. For animal studies, include a statement about blinding even if no blinding was done	Details are mentioned in the materials and methods section.
5. For every figure, are statistical tests justified as appropriate?	Yes.
Do the data meet the assumptions of the tests (e.g., normal distribution)? Describe any methods used to assess it.	Yes. The detailed statstical methods are mentioned in the respective figure legends.
Is there an estimate of variation within each group of data?	Yes.

Is the variance similar between the groups that are being statistically compared?	Yes. Data were processed using standard procedures to ensure that the variance was similar between different groups

C- Reagents

6. To show that antibodies were profiled for use in the system under study (assay and species), provide a citation, catalog number and/or clone number, supplementary information or reference to an antibody validation profile. e.g., Antibodypedia (see link list at top right), 1DegreeBio (see link list at top right).	The informaiton of antibodies including catalog number and/or clone number has been provided in the Materials and Methods section of the manuscript.
7. Identify the source of cell lines and report if they were recently authenticated (e.g., by STR profiling) and tested for mycoplasma contamination.	No commonly misidentified cell lines were used in this study. All cells were mycoplasma-free with regular checks performed by a LookOut Mycoplasma PCR (i.e., polymerase chain reaction) Detection Kit (MP0035, Sigma-Aldrich).

* for all hyperlinks, please see the table at the top right of the document

D- Animal Models

8. Report species, strain, gender, age of animals and genetic modification status where applicable. Please detail housing and husbandry conditions and the source of animals.	Details are described in the materials and methods section.
9. For experiments involving live vertebrates, include a statement of compliance with ethical regulations and identify the committee(s) approving the experiments.	All mouse experiments were performed in accordance with the University Health Guide for the Care and Use of Laboratory Animals and were approved by the Biological Research Ethics Committee of Tongji University.
10. We recommend consulting the ARRIVE guidelines (see link list at top right) (PLoS Biol. 8(6), e1000412, 2010) to ensure that other relevant aspects of animal studies are adequately reported. See author guidelines, under 'Reporting Guidelines'. See also: NIH (see link list at top right) and MRC (see link list at top right) recommendations. Please confirm compliance.	All experiments were approved by Tongji University School of Medicine Animal Care and Use Committee and were conducted in accordance with the National Institutes of Health U.S.A (NIH) Guidelines for the Care and Use of Laboratory Animals.

E- Human Subjects

11. Identify the committee(s) approving the study protocol.	NA
12. Include a statement confirming that informed consent was obtained from all subjects and that the experiments conformed to the principles set out in the WMA Declaration of Helsinki and the Department of Health and Human Services Belmont Report.	NA
13. For publication of patient photos, include a statement confirming that consent to publish was obtained.	NA
14. Report any restrictions on the availability (and/or on the use) of human data or samples.	NA
15. Report the clinical trial registration number (at ClinicalTrials.gov or equivalent), where applicable.	NA
16. For phase II and III randomized controlled trials, please refer to the CONSORT flow diagram (see link list at top right) and submit the CONSORT checklist (see link list at top right) with your submission. See author guidelines, under 'Reporting Guidelines'. Please confirm you have submitted this list.	NA
17. For tumor marker prognostic studies, we recommend that you follow the REMARK reporting guidelines (see link list at top right). See author guidelines, under 'Reporting Guidelines'. Please confirm you have followed these guidelines.	NA

F- Data Accessibility

18: Provide a "Data Availability" section at the end of the Materials & Methods, listing the accession codes for data generated in this study and deposited in a public database (e.g. RNA-Seq data: Gene Expression Omnibus GSE39462, Proteomics data: PRIDE PXD000208 etc.) Please refer to our author guidelines for 'Data Deposition'.<br><br>Data deposition in a public repository is mandatory for:<br>a. Protein, DNA and RNA sequences<br>b. Macromolecular structures<br>c. Crystallographic data for small molecules<br>d. Functional genomics data<br>e. Proteomics and molecular interactions	The RNA-Seq data from this publication have been deposited to the GEO database [https://www.ncbi.nlm.nih.gov/geo/query/acc.cgi?acc=GSE166850] and assigned the identifier [GSE166850])
19. Deposition is strongly recommended for any datasets that are central and integral to the study; please consider the journal's data policy. If no structured public repository exists for a given data type, we encourage the provision of datasets in the manuscript as a Supplementary Document (see author guidelines under 'Expanded View' or in unstructured repositories such as Dryad (see link list at top right) or Figshare (see link list at top right).	NA
20. Access to human clinical and genomic datasets should be provided with as few restrictions as possible while respecting ethical obligations to the patients and relevant medical and legal issues. If practically possible and compatible with the individual consent agreement used in the study, such data should be deposited in one of the major public access-controlled repositories such as dbGAP (see link list at top right) or EGA (see link list at top right).	NA
21. Computational models that are central and integral to a study should be shared without restrictions and provided in a machine-readable form. The relevant accession numbers or links should be provided. When possible, standardized format (SBML, CellML) should be used instead of scripts (e.g. MATLAB). Authors are strongly encouraged to follow the MIRIAM guidelines (see link list at top right) and deposit their model in a public database such as Biomodels (see link list at top right) or JWS Online (see link list at top right). If computer source code is provided with the paper, it should be deposited in a public repository or included in supplementary information.	NA

G- Dual use research of concern

22. Could your study fall under dual use research restrictions? Please check biosecurity documents (see link list at top right) and list of select agents and toxins (APHIS/CDC) (see link list at top right). According to our biosecurity guidelines, provide a statement only if it could.	NA
